# Peripherally targeted analgesia via AAV-mediated sensory neuron–specific inhibition of multiple pronociceptive sodium channels

**DOI:** 10.1172/JCI170813

**Published:** 2024-05-09

**Authors:** Seung Min Shin, Brandon Itson-Zoske, Fan Fan, Yucheng Xiao, Chensheng Qiu, Theodore R. Cummins, Quinn H. Hogan, Hongwei Yu

**Affiliations:** 1 Department of Anesthesiology, Medical College of Wisconsin, Milwaukee, Wisconsin, USA.; 2 Department of Physiology, Medical College of Georgia, Augusta University, Augusta, Georgia, USA.; 3 Department of Biology, School of Science, Indiana University-Purdue University, Indianapolis, Indiana, USA.; 4 Department of Orthopedic Surgery, Qingdao Municipal Hospital, Qingdao, China.

**Keywords:** Neuroscience, Therapeutics, Gene therapy, Pain, Sodium channels

## Abstract

This study reports that targeting intrinsically disordered regions of the voltage-gated sodium channel 1.7 (Na_V_1.7) protein facilitates discovery of sodium channel inhibitory peptide aptamers (Na_V_iPA) for adeno-associated virus–mediated (AAV-mediated), sensory neuron–specific analgesia. A multipronged inhibition of I_Na1.7_, I_Na1.6_, I_Na1.3_, and I_Na1.1_ — but not I_Na1.5_ and I_Na1.8_ — was found for a prototype and named Na_V_iPA1, which was derived from the Na_V_1.7 intracellular loop 1, and is conserved among the TTXs Na_V_ subtypes. Na_V_iPA1 expression in primary sensory neurons (PSNs) of dorsal root ganglia (DRG) produced significant inhibition of TTXs I_Na_ but not TTXr I_Na_. DRG injection of AAV6-encoded Na_V_iPA1 significantly attenuated evoked and spontaneous pain behaviors in both male and female rats with neuropathic pain induced by tibial nerve injury (TNI). Whole-cell current clamp of the PSNs showed that Na_V_iPA1 expression normalized PSN excitability in TNI rats, suggesting that Na_V_iPA1 attenuated pain by reversal of injury-induced neuronal hypersensitivity. IHC revealed efficient Na_V_iPA1 expression restricted in PSNs and their central and peripheral terminals, indicating PSN-restricted AAV biodistribution. Inhibition of sodium channels by Na_V_iPA1 was replicated in the human iPSC-derived sensory neurons. These results summate that Na_V_iPA1 is a promising analgesic lead that, combined with AAV-mediated PSN-specific block of multiple TTXs Na_V_s, has potential as a peripheral nerve–restricted analgesic therapeutic.

## Abstract 2

Primary sensory neuron–specific inhibition of multiple pronociceptive sodium channels provides a strategy for peripheral analgesia.

## Introduction

Voltage-gated sodium channels (Na_V_s) are key regulators of neuronal excitability and pain sensations (1). Mammals possess 9 isoforms of Na_V_s, of which Na_V_1.7, Na_V_1.8, and Na_V_1.9 are preferentially expressed in the primary sensory neurons (PSNs) of dorsal root ganglia (DRG) (2). The prominent roles of these Na_V_ isoforms in human pain have been validated (2). Na_V_1.6, Na_V_1.1, and Na_V_1.3 are also expressed in PSNs and have been reported as possible targets for analgesics (3, 4). Currently, Na_V_1.7 is the leading target among Na_V_s for developing analgesic therapies (5).

Numerous efforts have been made over the past decades to develop selective and effective Na_V_1.7 blockers to treat pain in the clinic (6), but the success has been limited. Most of the available small-molecule Na_V_1.7 blockers tested to treat pain are insufficient in target engagement, lack targeting specificity or selective bioavailability in pain axis, and their global distribution contributes to cardiotoxicity, motor impairments, and CNS side effects (6). Development of biologics targeting Na_V_1.7 is an alternative growing-trend (7, 8) for analgesia. Na_V_1.7 neutralizing monoclonal antibodies have analgesic efficacy, but the results are inconsistent (6). Tarantula peptide Na_V_1.7 blockers are effective analgesics but have poor membrane permeability, inadequate Na_V_1.7 selectivity, and short half-lives (6). Na_V_1.7-RNAi (6) and CRISPR-dCas9 or ZEN epigenetic Na_V_1.7 suppression for analgesic gene therapy have been proposed (9), but these interventions at mRNA and epigenetic levels have a concern of lacking the specificity of direct channel intervention, reducing safety and permitting off-target effects (6, 10), and anti-Cas9 immunity creates an additional challenge for CRISPR gene therapies (11).

Small peptides derived from pronociceptive ion channels as functionally interfering peptide aptamers (iPA) are highly effective and selective, allowing block of specific nociceptive signaling (12, 13). Intrinsically disordered regions (IDRs) of ion channel proteins are commonly engaged in promiscuous interactomes, which are important players in multiple signaling regulations and are recognized as new and promising drug targets (14). We speculated that Na_V_1.7-IDRs contain short functional IDR domains that could play critical roles in modulating Na_V_1.7 functions and can be developed as Na_V_1.7iPAs (1.7iPA). Further, the high-level conservation of Na_V_ subtype sequences implies that a given 1.7iPA could interact with other Na_V_ subtypes that have homologous sequences to Na_V_1.7 and thereby enable multipronged engagement of Na_V_ subtypes. Because multiple PSN-Na_V_s contribute to nociceptive electrogenesis and pain pathogenesis, it is conceivable that AAV-mediated expression of such multipronged Na_V_iPA restricted in DRG-PSNs to inhibit several pronociceptive Na_V_s could be an analgesic advantage compared with a block of only a single Na_V_ subtype (15–17).

We here describe a strategy by which highly selective and nontoxic Na_V_iPAs were designed and developed from Na_V_s-IDRs. A prototypical Na_V_iPA1 derived from Na_V_1.7 intracellular loop 1 and conserved in Tetrodotoxin-sensitive (TTXs) Na_V_ subtypes showed multipronged inhibition of Na_V_1.7, Na_V_1.6, Na_V_1.3, and Na_V_1.1 channels. Na_V_iPA1 expression in rat PSNs rendered significant TTXs but not TTX-resistant (TTXr) I_Na_ inhibition. AAV-mediated Na_V_iPA1 expression selectively in the PSNs responsible for pain pathology in a rat pain model produced efficient analgesia while avoiding off-site biodistribution that causes side effects. Together, these results indicate that AAV-mediated PSN-specific, combined block of multiple nociceptive Na_V_s has potential for future therapeutic development.

## Results

### In silico design of 1.7iPAs from NaV1.7-IDRs

**Figure 1:**
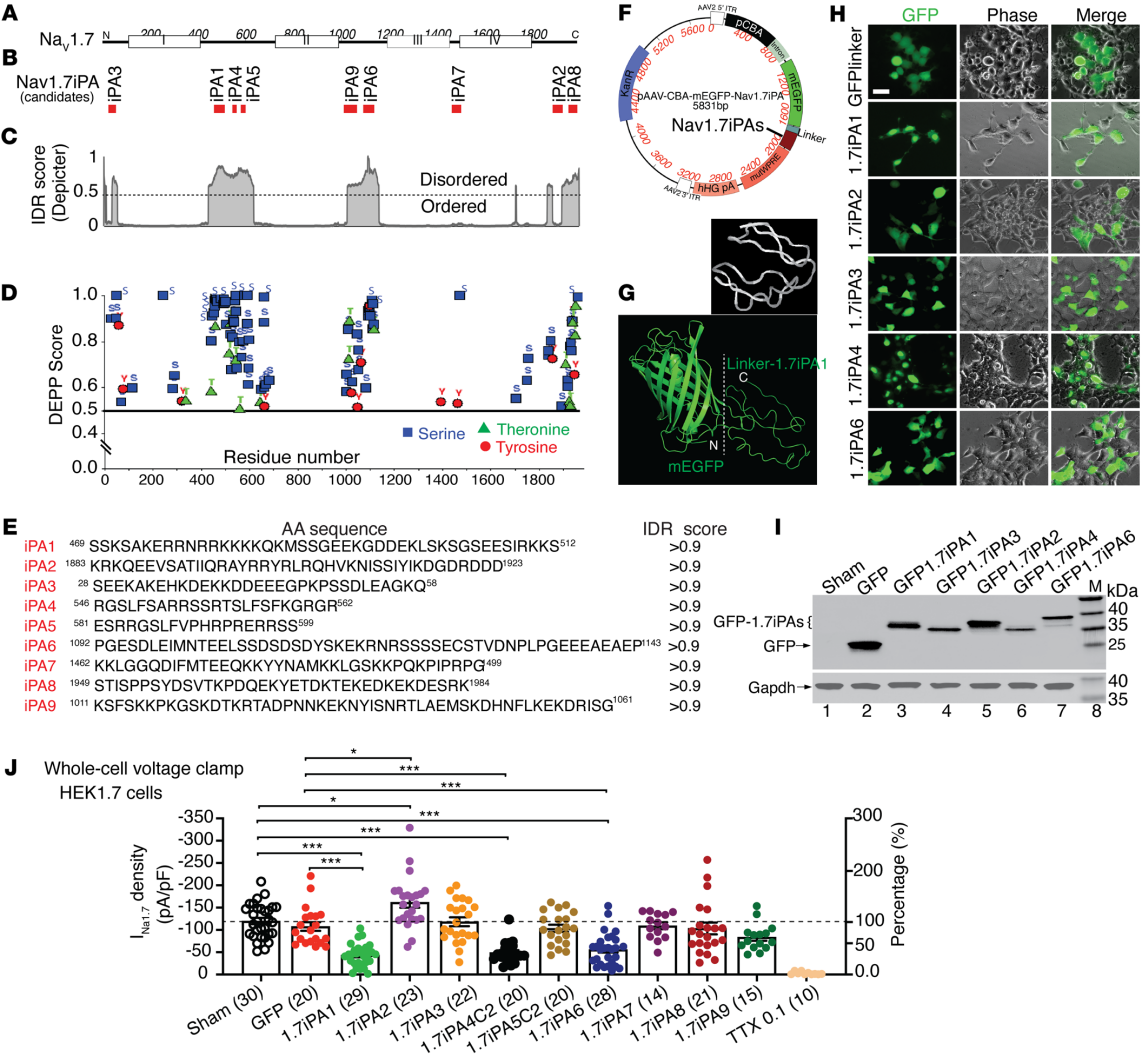
In silico prediction of Na_V_1.7 IDRs and design of candidate Na_V_1.7iPAs. (**A**) Diagram of rat Na_V_1.7 protein, with white boxes labeling DI-DIV of Na_V_1.7 and (**B**) the red bars below showing position of the predicted iPAs. (**C**) Consensus prediction of IDRs by DEPICTER. (**D**) The phosphorylation sites were predicted by DEPP. (**E**) Nine candidate iPAs with their aa sequences and position in Na_V_1.7, and IDR scores. (**F**) A map showing each component of an AAV plasmid coding GFP-iPA with a black line pointing to iPAs. (**G**) The structure analysis of GFP-fused 1.7iPA1 by I-TASSER. The top image shows structure of free 1.7iPA1. (**H**) Images (GFP, left; phase, middle; and merged pictures, right) show expression of constructs carrying 1.7iPA1–4 and 6 after transfection to HEK cells. Scale bar: 25 μm. (**I**) GFP and Gapdh Western blots of the cell lysates after transfection with 1.7iPA1-4 and 6 to HEK cells. (**J**) Initial screening of 9 iPAs on I_Na_ by whole-cell patch-clamp recording as described in methods after transfection into HEK1.7 cells. *P < 0.05 and ***P < 0.001; 1-way ANOVA and Tukey’s post hoc.

The candidate iPAs were designed through an a priori strategy aimed at defining the short linear functional disordered peptides from the intrinsically disordered domains (IDDs) (12), initially from Na_V_1.7 protein IDRs, on the hypothesis that Na_V_1.7 IDDs contain the functional sequences that modulate Na_V_1.7 channel function. We analyzed the full length of the rat Na_V_1.7 protein sequence using DisorderEd PredictIon CenTER (DEPICTER), which combines 10 popular algorithms for IDR predictions within the primary sequence based on amino acid (aa) biophysical features for the protein’s disordered ensemble (18). Results return a score between 0 and 1 for each residue, indicating the degree to which a given residue is part of an ordered or disordered region (residues with scores over 0.5 are considered as disordered). Results revealed clear order-to-disorder transitions where Na_V_1.7 transmembrane (TM) domains and intracellular portions join, and scores indicate a disordered nature of Na_V_1.7 intracellular and terminal regions (Figure 1, A–C). Specifically, the most extensive IDRs are in the intracellular loops (ICL), while protein TM domains are highly ordered.

Potential phosphorylation sites in the Na_V_1.7 sequence were identified using Disorder Enhanced Phosphorylation Predictor (DEPP) (19). Results showed that most potential phosphorylation residues (serine, threonine, and tyrosine with high DEPP scores) reside in Na_V_1.7-IDRs, particularly in the IDRs within the ICL1 and ICL2 (Figure 1D). Na_V_1.7-IDRs feature as potential protein-protein interaction (PPI) binding sites, suggesting these IDRs could contain key binding motifs or domains of the Na_V_1.7 regulatory signaling interactome (20). These observations predict that focusing on the Na_V_1.7-IDRs could be an avenue for identifying short peptides effective in modulating Na_V_1.7 channel function.

The potentially functional domains within the Na_V_1.7-IDRs (21) were further analyzed using SLiMPrints (22), which predict short linear motifs (SLiMs) based on strongly conserved primary aa sequences followed by filtering based on the prediction scores (22). The enumerated motifs predicted within Na_V_1.7-IDRs suggest many possible functional peptides as hot spots of functional IDDs, including proteolytic cleavage sites, ligand binding sites, posttranslational modification (PTM) sites, and subcellular targeting sites. Nine peptides were designed computationally based on IDR scores and phosphorylation sites and were the focus as 1.7iPA candidates for further testing (Figure 1, E and B).

### Constructs of 1.7iPAs and transfection expression

AAV expression plasmids containing transgene expression cassettes encoding various GFP-1.7iPA chimeras were constructed. Specifically, the sequences for interchangeable iPA peptides were cloned with a linker sequence (GLRSRAQASNSAVDGTAGPGS), which we have described previously (23), to form a chimeric transgene in a GFP-linker-iPA orientation transcribed by a hybrid human cytomegalovirus (CMV) enhancer/chicken β-actin (CBA) promoter. This generated pAAV-CBA-GFP-1.7iPAs (pAAV-1.7iPA) expression plasmids in which the oligonucleotide encoding the interchangeable 1.7iPAs are inserted at the 3′ end of GFP (Figure 1F). The predicted protein structure analysis of GFP1.7iPA1 by I-TASSER tool (24) shows an unfolded and extended, highly flexible structural ensemble of linker-1.7iPA1 (Figure 1G) that is compatible with a well-exposed mode for potential binding to targets. Similar structures were also identified by I-TASSER for other GFP1.7iPAs (Supplemental Figure 1; supplemental material available online with this article; https://doi.org/10.1172/JCI170813DS1).

### Inhibition of NaV1.7 current in HEK1.7 cells by 1.7iPAs

**Figure 2:**
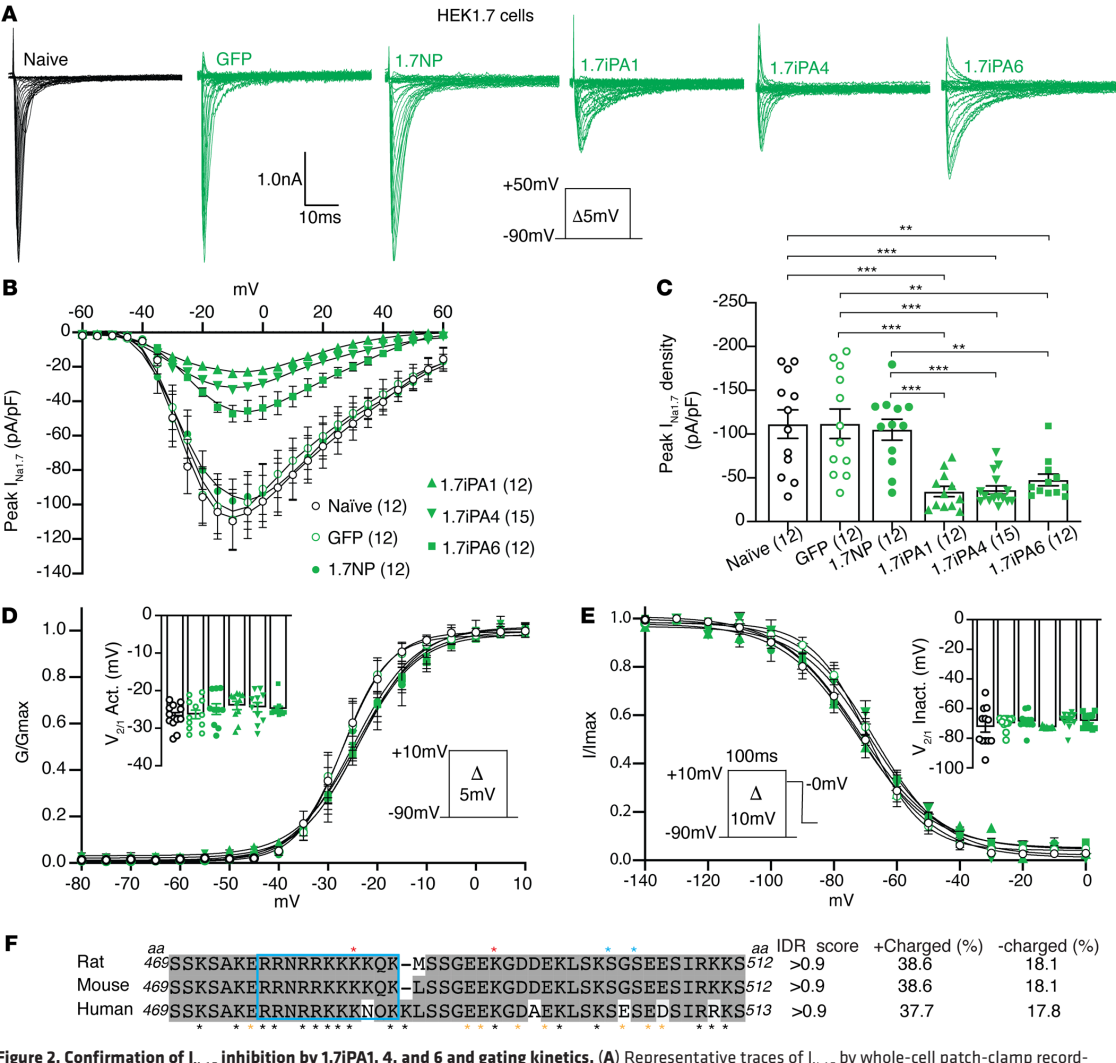
Confirmation of I_Na1.7_ inhibition by 1.7iPA1, 4, and 6 and gating kinetics. (**A**) Representative traces of I_Na1.7_ by whole-cell patch-clamp recording from naive (transfection without plasmid), GFP, 1.7iPA3 (NP), 1.7iPA1, 1.7iPA4, and 1.7iPA6-transfected HEK1.7 cells. Inserts: recording protocol and current/time scales. (**B**) Summary of the confirmation tests of candidate iPAs expression in HEK1.7 cells (**C**) in comparison with corresponding mean peak current density-voltage (I/V) relationship from different constructs, as indicated and quantitative analysis of averaged peak I_Na1.7_ density; **P < 0.01, ***P < 0.001, 1-way ANOVA and Tukey’s post hoc. (**D**)No effects of expression of GFPiPA1, GFPiPA4, and GFPiPA6 were observed on steady-state activation (inset: V1/2 activation) and (**E**) fast inactivation (inset: V1/2 inactivation), compared with naive and GFP or NP-transfected HEK1.7 cells. (**F**) Na_V_iPA1 is highly conserved in rat, mouse, and human. Black and yellow asterisks at the bottom denote positively and negatively charged aa; the red and blue asterisks on the top denote known lysine acetylation and serine phosphorylation sites, and IDR scores and percent of positively (+) and negatively (–) charged aa were shown at the right sides of the alignment.

The stable expression of each construct was verified by transfection into HEK293 cells stably expressing human WT Na_V_1.7 (HEK1.7 cells), followed by immunoblots (IBs). Representative tests for GFPlinker (GFP), 1.7iPAs (1, 2, 3, 4, 6) were shown (Figure 1, H and I). Initial screening experiments by whole-cell voltage clamp of inhibition of Na_V_1.7 current (I_Na1.7_) in HEK1.7 cells transfected with plasmids encoding 9 1.7iPAs (1.7iPA1-9) were performed to characterize the I_Na1.7_. The presence of 9 different 1.7iPAs in HEK1.7 cells on peak I_Na1.7_ density (3 days after transfection) was summarized in Figure 1F, in which the data points recorded by at least 2 replicates were combined (Figure 1J). The results showed that 1.7iPA1, 4, and 6 produced approximately 68%, 59%, and 54% reduction of peak I_Na1.7_ density, respectively, while 1.7iPA2 increased peak I_Na1.7_ density (~35%). Transfection with plasmids expressing the GFPlinker and 1.7iPA3, 5, 7, 8, and 9 showed no significant effects on peak I_Na1.7_ density, compared with sham-transfected HEK1.7 cells, which were transfected with PEI but no plasmid. These experiments thus identified 1.7iPA1 and 1.7iPA4, both of which were derived from ICL1, as well as 1.7iPA6, derived from ICL2, as effective iPAs (over 50% I_Na1.7_ inhibition). We next focused on the validation of I_Na1.7_ inhibition and channel kinetics by 1.7iPA1, 4, and 6 on HEK1.7 cells in new experiments. These results replicated the prior screening testing results of peak I_Na1.7_ densities and showed that the steady-state activation and fast inactivation kinetics of Na_V_1.7 channels were not significantly affected in the presence of 1.7iPA1, 4, and 6 (Figure 2, A–E). The 1.7iPA1 peptide is polyampholytic, enriched with 38.6 % positively charged arginine or lysine (17 of 44), 22.7% of serine (10 of 44), and 18.1% acidic residues (8 of 44) and is highly conserved between rodents and humans (Figure 2F). Searching databases revealed that 2 serine phosphorylation and 2 lysine acetylation sites were assigned in high throughput (proteomic discovery mass spectrometry) studies (25) and a nuclear localization signal was predicted by SeqNLS (26). These analyses strongly suggest that 1.7iPA1 is a functional IDD peptide. Since 1.7iPA1 revealed higher inhibition of I_Na1.7_ and was highly homologous to other TTXs Na_V_ subtypes (see further), we selected it as a prototype and named Na_V_iPA1 for further ‘hit to lead’ characterization.

### Specificity of NaViPA1 occupancy to various voltage-gated ion channels

#### Development of Na_V_1.8 stable expression system based on HEK cells.

To assess the potential of Na_V_iPA1 in affecting I_Na_ conducted by Na_V_1.8 channels, we developed stable expression of recombinant human Na_V_1.8 heterologous systems based on HEK cells (HEK1.8). Stable Na_V_1.8 expression was confirmed by IBs of Na_V_1.8α and Naβ2 in the cells after at least 10–20 rounds of G418 selection (400–800 μg/mL), followed by single-cell isolation using BIOCHIPS Single-cell Isolation Chip (Thermo Fisher Scientific). Both Na_V_1.8α and Naβ2 were found to be highly expressed in the cell membrane. Functional Na_V_1.8 expression was identified by the presence of slowly inactivating inward I_Na_ elicited by voltage steps from –140 mV to +80 mV during the whole-cell voltage-clamp recordings, and the averaged peak I_Na1.8_ density in approximately 85% of the HEK1.8 was greater than 0.5 nA; I_Na1.8_ was sensitive to a Na_V_1.8 channel blocker, A803467 (Alomone) and resistant to high concentration of TTX (5 μM, Tocris Bioscience). We used this HEK1.8 cell line for the initial screening tests of the Na_V_iPA1 on I_Na1.8_. In comparison, I_Na1.8_ amplitudes in CHO-Nav1.8 cells were generally less than 100 pA, which was insufficient for our experimental needs (Supplemental Figure 2).

#### Selectivity of Na_V_iPA1 on ion channel occupancy.

**Figure 3:**
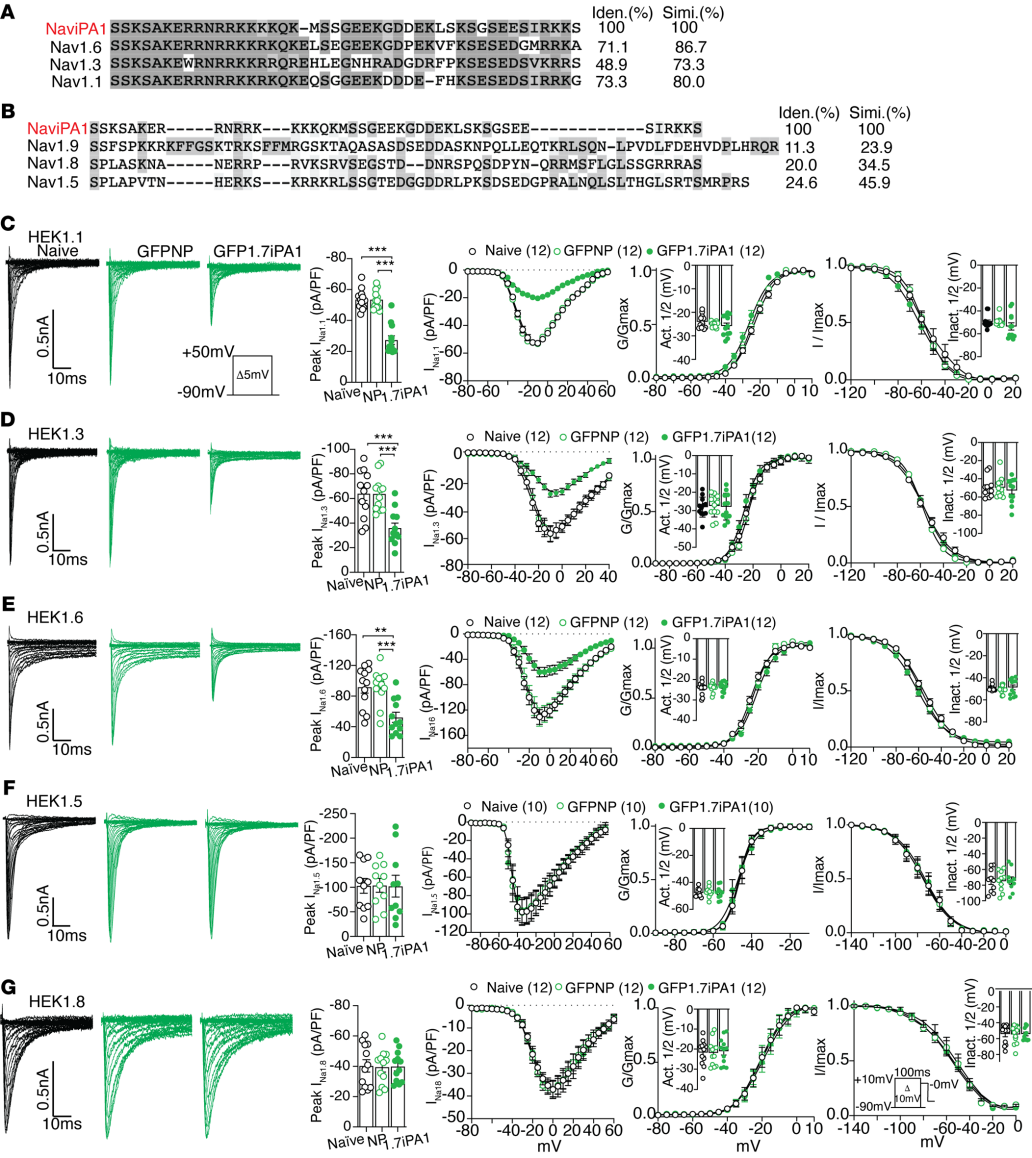
Sodium channel specificity of Na_V_iPA1 (1.7iPA1) inhibition. (**A**) The aa sequence alignment of 1.7iPA1 with the corresponding sequences of TTXs Na_V_1.6, Na_V_1.3, Na_V_1.1, (**B**) as well as TTXr Na_V_1.5, Na_V_1.8, and Na_V_1.9 of rat proteins. The homologous aa (identity and similarity) was highlighted in heavy or light black shadows and percent of identical or similar aa shown at the right sides of the alignments. (**C**–**G**) Panels from left to right show the comparisons of I_Na_ traces in presence of 1.7iPA1 in HEK1.1, 1.3, 1.6, 1.5, and 1.8 cells (insert: pulse protocol and scale); peak I_Na_ density (**P < 0.01 and ***P < 0.001, 1-way ANOVA and Turkey’s post hoc), I/V curves, steady-state activation (insert: V1/2 activation) and fast inactivation kinetics (insert: V1/2 inactivation).

Na_V_ subtype stable cell lines based on HEK cells used for this experiment included HEK1.1, 1.3, 1.6, 1.5, and 1.8. Sequence alignments identified high-level homology of Na_V_iPA1 with the corresponding sequences of TTXs Na_V_1.1, 1.3, and 1.6, but much less homologous to TTXr Na_V_1.5, 1.8, and 1.9 (Figure 3, A and B). Expression of Na_V_iPA1 (fused to GFP) resulted in a significant block of I_Na_ conducted by fast-activating and inactivating Na_V_1.1, Na_V_1.3, and 1.6 (Figure 3, C–E). No effects on I_Na1.5_ and I_Na1.8_ were observed in the presence of Na_V_iPA1 in the HEK1.5 and HEK1.8 cells (Figure 3, F and G) or in ND7/23 cells transiently transfected with Nav1.8 (Supplemental Figure 3). We did not test Na_V_iPA1 against Na_V_1.9 channels as the expression cell line is unavailable; however, I_Na1.9_ inhibition by 1.7iPA1 is not expected since there is no sequence homology of Na_V_iPA1 to Na_V_1.9. The negative effects of Na_V_iPA1 on potassium current (BK I_Kv_) were found in NG108-15 cells, which naturally express potassium channels (12), and no effects on high-voltage activated (HVA) I_Ca_ were recorded on AAV-mediated Na_V_iPA1 expression in DRG-PSNs. Potent I_Na1.7_ inhibition by Na_V_iPA1 was also confirmed in neuronal NG108-15 cells and F11 DRG-neuronal-like cells that naturally express Na_V_1.7. These experiments showed no pleiotropic effects of Na_V_iPA1 on either BK potassium channels or HVA I_Ca_ (Supplemental Figure 4).

#### AAV6-mediated Na_V_iPA1 expression in DRG-PSNs inhibits TTXs I_Na_ but not TTXr I_Na._

**Figure 4:**
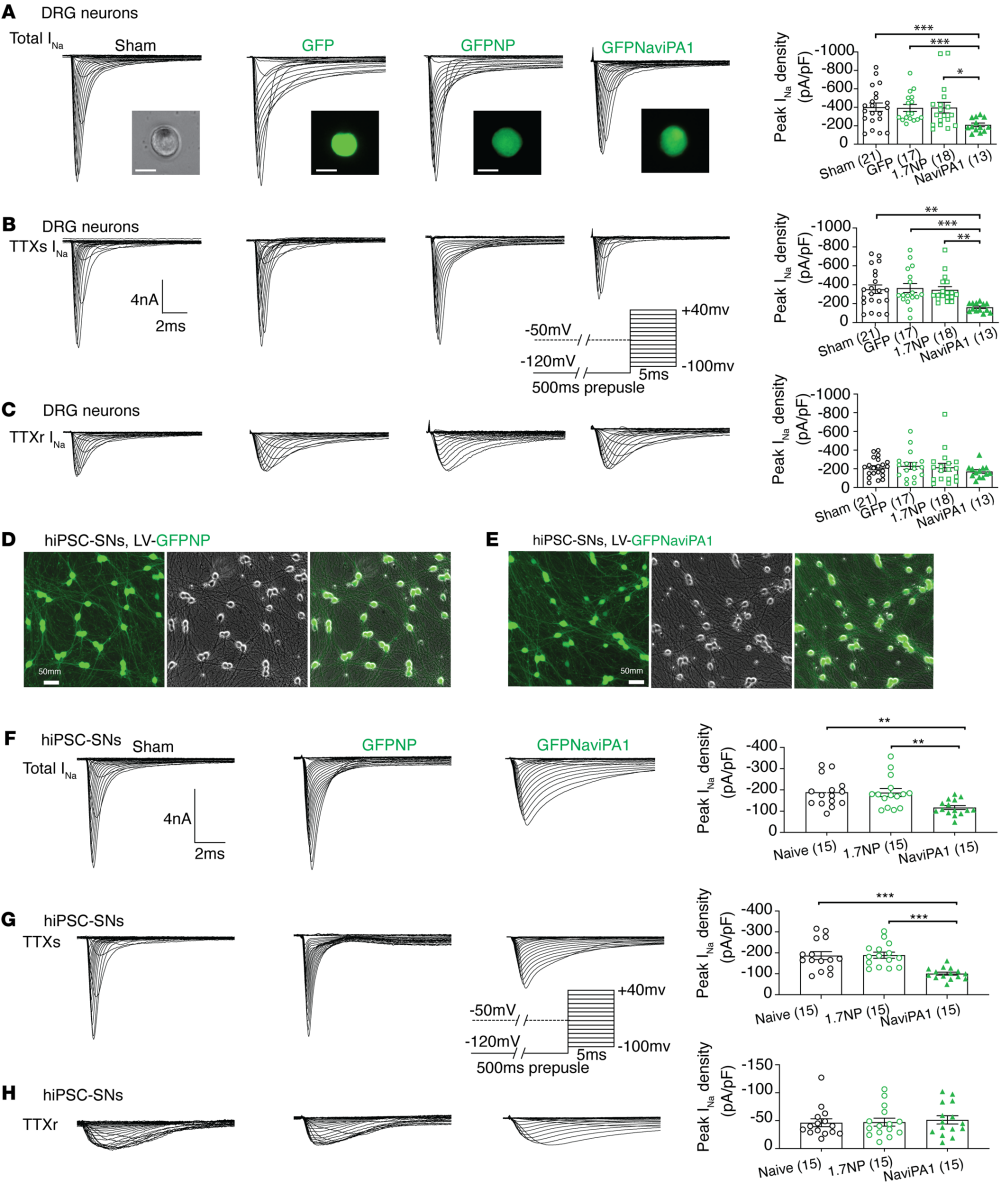
Na_V_iPA1 on I_Na_ of rat DRG neurons (male) and hiPSC-SNs (female). (**A**–**C**) Panels from top to bottom illustrate representative traces and averaged peak I_Na_ densities of total I_Na_ (**A**), TTXs I_Na_ (**B**), and TTXr I_Na_ (**C**) recorded from sensory neurons (diameter < 35 μm) dissociated from naive male rats subjected to (panels from left to right) sham (surgical exposure without injection), and 4wk after L4/L5 DRG injected with AAV6-encoded GFP, GFPNP, and GFPNa_V_iPA1. Inserts: representative PSN images (scale bars: 25 μm) of each group, current/time scales, and recording pulse protocol. (**D** and **E**) Representative montage ICC images illustrate hiPSC-SNs at DIV25 after transduction with LV-GFPNP (**D**) and LV-GFPNa_V_iPA1 (**E)** at equal MOI = 5. Panels **F**–**H** illustrate representative traces and averaged peak I_Na_ densities of total I_Na_ (**F**), TTXs I_Na_ (**G**), and TTXr I_Na_ (**H**) recorded from hiPSC-SNs (DIV 25) of sham, expressing NP, and Na_V_iPA1. Inserts: current/time scales and recording pulse protocol. *P < 0.05, **P < 0.01, and ***P < 0.001, 1-way ANOVA and Turkey’s post hoc.

Because no heterologous system or cell lines can fully mimic the in vivo conditions of sensory neurons, we further tested the functional inhibition of I_Na_ by Na_V_iPA1 in DRG-PSNs. AAV6 vectors encoding GFP-fused Na_V_iPA1 were generated and injected into lumbar vertebrae (L) 4 and/or 5 DRG of naive male rats, and acutely dissociated sensory neurons from DRG were tested 4 weeks after injection. AAV6 encoding GFPlinker and NP (1.7iPA3), which was derived from the N-terminus of Na_V_1.7 (Figure 1) and showed no effect on I_Na_ after being transfected into HEK1.7 (Figures 1 and 2), were used as the control. A voltage protocol was adopted that demonstrates successful separation of TTXr I_Na_ (Nav1.8-like) and TTXs I_Na_ in dissociated DRG neurons (27, 28), comparable to the recordings after addition of TTX (1.0 μM) in bath solution (Supplemental Figure 5, A and B). Whole-cell voltage-clamp recordings by the voltage protocol from small/medium-sized PSNs (less than 35 μm) showed that AAV-mediated expression of Na_V_iPA1 produced significant inhibition of total and TTXs I_Na_ whereas it produced no significant inhibition on TTXr I_Na_ (Figure 4, A–C).

#### Inhibition of TTXs I_Na_ by Na_V_iPA1 in human iPSC-derived sensory neurons.

To study the relevance of our findings in a human context, we used human induced pluripotent stem cell–derived (hiPSC-derived) sensory neurons (hiPSC-SNs, female, Anatomic) (29) to test whether inhibition of TTXs I_Na_ by Na_V_iPA1 represents a meaningful and quantitative index of the functional lead in human sensory neurons. This also allowed examination Na_V_iPA1 without potential overexpression effects in HEK-Na_V_ cells. The hiPSC-SNs were differentiated to small-sized PSN morphology with a soma diameter around 20–25 μm and developed extensive neurites after 4–7 days in vitro (DIV) in differentiation cultures, indicating that these cells were sufficiently committed to the neuronal lineage. We used lentivector (LV) GFP (Supplemental Figure 6) to test hiPSC-SN transduction efficiency. We have succeeded in expressing Na_V_iPA1 and 1.7NP (control) in the differentiated hiPSC-SNs by LV transduction at multiple of infection (MOI) equaling 5 (Figure 4, D and E). Electrophysiological recordings were performed on the hiPSC-SNs (DIV 25) with TTX (1 μΜ) in the bath solution, and TTXr/TTXs I_Na_ were separated by a subtraction protocol (27). To prevent the TTX effect, a voltage manipulation similar to DRG neuron recording was used. Additionally, a protocol was adopted to isolate somatic I_Na_ by a brief prepulse to voltage (40 mV) near to a spike inactivating voltage for hiPSC-SN axonal spikes, but not for somatic spikes (30). Results showed that Na_V_iPA1 significantly inhibited TTXs I_Na_ but not TTXr I_Na_ in differentiated hiPSC-SNs (DIV 25) (Figure 4, F–H), comparable to rat DRG-PSNs. No effects were observed for BK I_Kv_ and HAV I_Ca_ recorded (DIV 21) from hiPSC-SNs in the presence of Na_V_iPA1 (Supplemental Figure 7). Results indicate that inhibitory efficacy of Na_V_iPA1 on TTXs I_Na_ defined in cell lines and rat DRG-PSNs are translatable to human PSNs.

### Initial testing of molecular mechanisms of NaViPA1

**Figure 5:**
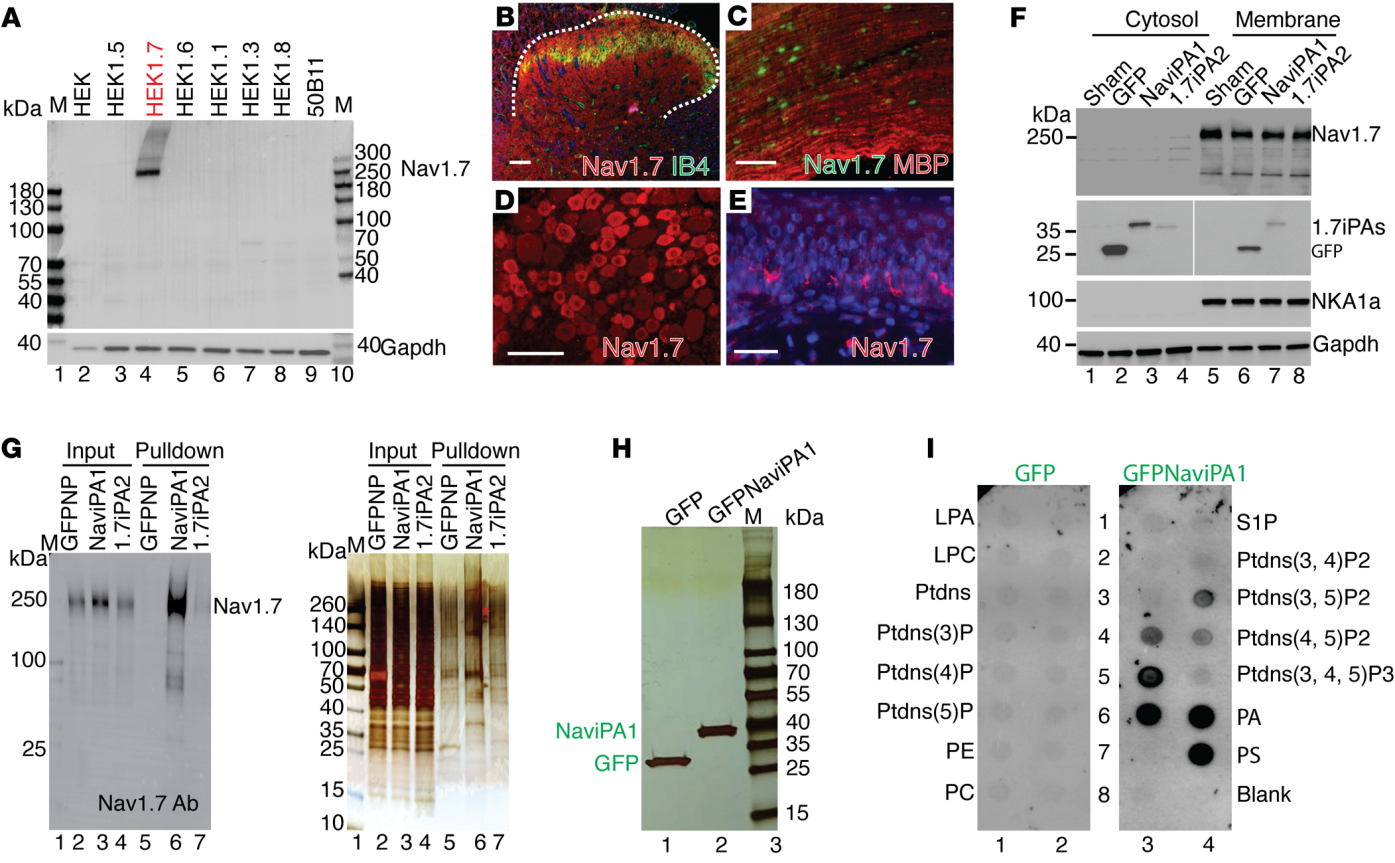
Na_V_iPA1 binds to full-length Na_V_1.7 protein and phosphoinositides. (**A**) IBs show selectivity of Na_V_1.7 antibody using cell lysates from naive HEK cells, HEK1.5, HEK1.7, HEK1.6, HEK1.1, HEK1.3, HEK1.8 cells, and 50B11 cells. (**B**–**E**) Representative IHC images show Na_V_1.7 detection (red) in SDH (red), sciatic nerve (green), DRG neurons (red), and cutaneous nerve fibers (red). Scale bars: 100 μm. (**F**) IBs of Na_V_1.7, GFP, NKA1α, and Gapdh in the cytosol and membrane samples extracted from HEK1.7 cells transfected with sham (transfection without plasmid), GFP, GFPNa_V_iPA1, and GFP1.7iPA2. A vertical white line in GFP panel denotes that the lanes were run on the same gel but were noncontiguous. (**G**) Na_V_1.7 IB (left) and silver stain (right) of inputs (cell lysates, 20 μg for each lane) and pulldown beads (10 μL for each lane) prepared by a nondenaturing lysis buffer from HEK1.7 cells transfected with GFP, GFPNa_V_iPA1, and GFP1.7iPA2. (**G,** right) Stained gel pieces ranging 100–300 kDa (**G**, red asterisk denotes Na_V_1.7 site) from GFPNP and GFPNa_V_iPA1 excised for mass spectrometry. (**H**) Silver stain on 1D SDS-PAGE gel of GFP-affinity pulldown beads in the NG108-15 cells transfected with GFPNa_V_iPA1 and GFP and cell lysates prepared using denaturing RIPA buffer (**I**) and the results of PIP strip analysis.

We first validated the specificity of Na_V_1.7 antibody by IB using the cell lysates prepared from naive HEK cells, stable cell lines expressing different Na_V_ isoforms, and 50B11 rat DRG neuronal cells. This Na_V_1.7 antibody (Alomone, ASC-008) was raised by an antigenic peptide corresponding to amino acid residues 446–460 of rat Na_V_1.7 and no significant sequence homologous with other Na_V_ isoforms. Results showed that the Nav1.7 antibody detected full-length Na_V_1.7 only in HEK1.7 cells, but not other Na_V_ isoforms and 50B11 cells that naturally do not express physical and functional Na_V_1.7 (31) (Figure 5A). By IHC on rat tissue sections, Na_V_1.7 expression was detected with high immunoreactive density in small/medium-sized PSNs using the Na_V_1.7 antibody, and Na_V_1.7 was also detected in spinal cord dorsal horn (SDH), sciatic nerve, and cutaneous terminals in hindpaws (Figure 5, B–E), with the patterns similar to the prior report (32). These results confirmed the specificity of the Na_V_1.7 antibody to detect Na_V_1.7 expression by IHC and IB.

Since Na_V_1.7 is an integral membrane protein, we therefore tested whether Na_V_iPA1 expression in the HEK1.7 cells would interrupt Na_V_1.7 intracellular trafficking. Our results do not support this mechanism since no clear reduction of membrane Na_V_1.7 protein was evident in the fractionized preparations, from HEK1.7 cells transfected with Na_V_iPA1 and controls (Figure 5F). Studies have shown that IDRs in the membrane proteins engage in interactions with the membrane (33). To test whether Na_V_iPA1 interference of Na_V_1.7 might be via direct block of Na_V_1.7, GFP affinity pull-down by ChromoTek GFP-Trap (ChromoTek) was performed after transfection of GFP-Na_V_iPA1 in HEK1.7 cells using GFPlinker and GFP-1.7iPA2 (Figure 1) as the controls. Cell lysates were prepared by a lysis buffer containing 0.5% Nonidet p40, a nondenaturing mild lysis detergent, for preventing interaction breaking and maximizing the retention of Na_V_iPA1-protein interactions (34). IBs verified full-length Na_V_1.7 protein trapped in the GFPNa_V_iPA1 pull-down sample but not in controls (Figure 5G, NaViPA1 pull-down with NaV1.7 was confirmed in an additional experiment), and nano liquid chromatography mass spectrometry (nLC-MS/MS) detection of unique hNa_V_1.7 peptides (Table 1) confirmed hNa_V_1.7 on the excised band from silver-stained sodium dodecyl sulfate–polyacrylamide electrophoresis (SDS-PAGE) gel (Figure 5G, right panel) of GFPNa_V_iPA1 affinity pull-down sample. These results indicate that Na_V_iPA1 block of Na_V_1.7 channel activation could be via binding to the Na_V_1.7 protein, i.e., an intramolecular domain-domain interaction (intraDDI) (35). It has been reported that polybasic IDRs in TM proteins preferably bind to negatively charged lipids (36, 37). We reasoned that Na_V_iPA1 might be able to bind phosphoinositides, and this hypothesis was tested by using phosphatidylinositol phosphate (PIP) strips (Echelon PIP Strip). GFPNa_V_iPA1 and GFP (control) were transfected into neuronal NG108-15 cells, and cell lysates were prepared by a RIPA buffer containing 0.1% SDS with 1% Triton X100, both strong detergents, and 1% deoxycholate, an anionic detergent, for maximal denaturing to break Na_V_iPA1 PPI complex formations. Silver stain after SDS-PAGE gel showed clean purification of GFP and GFPNa_V_iPA1 (Figure 5H) and samples were applied to the PIP strips. Results (Figure 5I, repeat twice) showed that GFP Na_V_iPA1 was efficiently bound to a number of anionic PIPs, PIP2, phosphatidic acid (PA), and phosphatidylserine (PS). In contrast, affinity pull-down GFP did not show clear binding to lipid spots, as previously reported (38). This is consistent with the reports that basic residues, often clustered in IDRs, can modulate membrane protein functions by binding via electrostatic interactions with lipids (39, 40).

**Figure 6:**
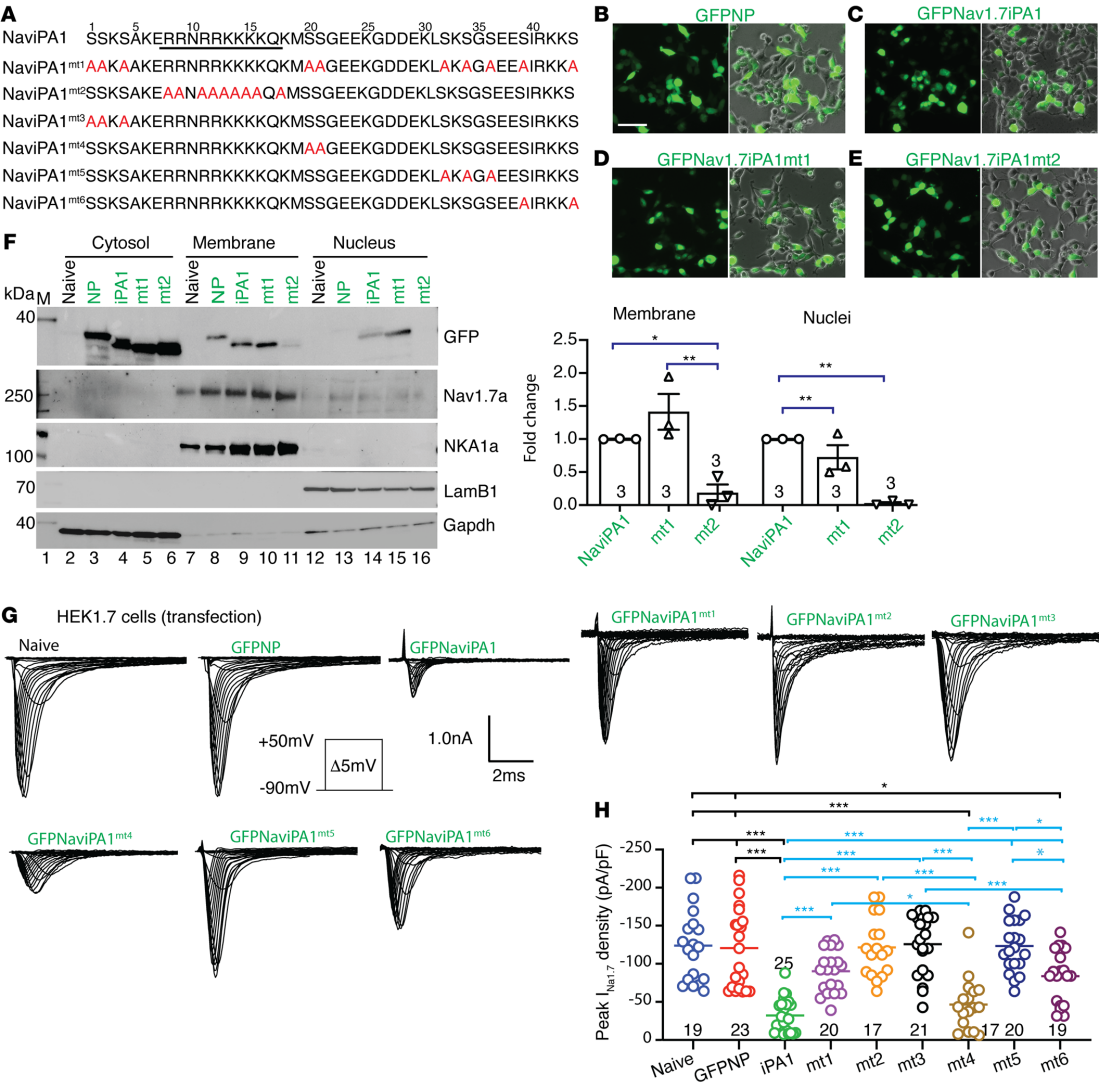
Define polybasic NLS and adjacent serine in NaviPA1. (**A**) Sequence alignments of NaviPA1, mutant 1 (mt) with alanine substitution of ten serine residues, mt2 by alanine substitution of arginine/lysine (R/K) (mt2) within predicted NLS domain, and mt3–6 with alanine substitution of bi- or triserine residues at different serine sites, as indicated. (**B**–**E**) ICC comparison of GFP signals 48 hours after plasmids coding NaviPA1, GFPNP, mt1, and mt2 transfected into HEK1.7 cells. Scale bar: 100 μm. (**F**, left) Representative IBs of endogenous Nav1.7, as well as GFPNP, NaviPA1, mt1, and mt2, in extracted cytosol, membrane, and nuclear samples after transfection into HEK1.7 cells. Cytosol, membrane, and nuclear loading were indicated by GAPDH, NKA1α, and LamB1, respectively. (**F**, right) Quantitative (ImageJ gel analysis) comparison of membrane binding and nuclear entry of NaviPA1, mt1, and mt2 after transfection, *P < 0.05, **P < 0.01, 1-way ANOVA and Tukey’s post hoc. (**G**) Representative I_Na1.7_ traces of HEK1.7 cells recorded from sham, GFPNP, NaviPA1, mt1–mt6 (3–4 days after transfection), as indicated. (**H**) Quantification summary of peak I_Na_ densities; *P < 0.05, **P < 0.01, and ***P < 0.001; 1-way ANOVA and Tukey’s post hoc.

Na_V_iPA1 is a polybasic arginine/lysine and serine-enriched peptide (Figure 2F). Protein-conserved polybasic domains with adjacent serine PTMs often play roles in protein function (41–43). We designed experiments to examine the role of polybasic NLS and multiple adjacent polyserine in the function of Na_V_iPA1. Initial tests were performed by generating Na_V_iPA1 mutant 1 **(**Na_V_iPA1mt1) (GFP-fused) in which alanine substitution for ten serine residues within Na_V_iPA1 was made, and Na_V_iPA1mt2 (GFP-fused) was generated by alanine substitution for 9 arginine or lysine within the predicted polybasic NLS of Na_V_iPA1 (Figure 6A). ICC showed that nuclear localization of Na_V_iPA1 (HEK1.7 cell transfection) was observed in Na_V_iPA1 and Na_V_iPA1mt1 but diminished in Na_V_iPA1mt2 (Figure 6, B–E). With a comparable transfection rate at approximately 40% for each construct, IBs revealed that Na_V_iPA1 was detected in the extracted cytosol, membrane, and nuclear samples, and that the membrane-binding and nuclear entry signals in Na_V_iPA1mt1 were comparable to Na_V_iPA1 but both vanished in Na_V_iPA1mt2. Full-length Na_V_1.7 was enriched in the membrane samples, as shown in Figure 5F, and the presence of Na_V_iPA1, mt1 and mt2 in HEK1.7 cells appeared not to impede Na_V_1.7 protein membrane integration (Figure 6F). Whole-cell voltage-clamp recording showed that I_Na1.7_ in the presence of Na_V_iPA1mt1 and mt2 was comparable to naive and GFP-NP transfected HEK1.7 cells, suggesting that both polybasic arginine/lysine and multiple adjacent serine residues were required for Na_V_iPA1 inhibitory effect on Na_V_1.7 current. To further map the critical serine sites, we generated additional Na_V_iPA1mt3–mt6 with alanine substitution for dual or triple serine residues (Figure 6A). Whole-cell voltage-clamp recordings showed that Na_V_iPA1mt3 and 5 with alanine substitution at different serine sites lost inhibitory effects on I_Na1.7_ after transfection to HEK1.7 cells while mt4 and mt6 showed a significant block of I_Na1.7_ (Figure 6, G and H). As expected, Na_V_iPA1mt1 and mt2 did not change I_Na1.8_ after being transfected to HEK1.8 cells, similar to Na_V_iPA1 (Supplemental Figure 8). These data suggest that conserved polybasic NLS and multiple adjacent serine residues within the Na_V_iPA1 are synergistic for I_Na1.7_ inhibition. The polybasic motif determines the polar association with the plasma membrane and nuclear entry of disordered Na_V_iPA1 peptide and multiple adjacent serine residues are required for Na_V_iPA1 inhibitory effect to I_Na1.7_. However, the full-length Na_V_1.7 membrane integration, which is determined by its TM domains but not intracellular sequences, was unaffected in the presence of Na_V_iPA1 (Figure 6F).

Future delineation of (a) the properties of serine and other residue PTMs within Na_V_iPA1 underlying inhibition of various TTXs I_Na_ in sensory neurons and (b) investigation of whether the presence of Na_V_iPA1 might undermine TTXs Nav channel activity via decoying interaction, diminishing PTMs in the full-length protein, and/or altering intradomain effects are of interest from both pathophysiological and therapeutic perspectives. Our goal in this study is to develop a strategy of peripherally targeted analgesia via AAV-mediated sensory neuron-specific inhibition of sodium channels. Therefore, in the following in vivo experiments, we focused on testing whether DRG-PSN–targeted expression of Na_V_iPA1 is effective in attenuating neuropathic pain behaviors.

### Analgesia after intraganglionic delivery of AAV-NaViPA1 in rats after TNI

We first conducted a pilot in vivo analgesia testing. High-titer and high-purity of AAV6-GFPNa_V_iPA1 (AAV6-Na_V_iPA1) and control AAV6-GFPNP (AAV6-NP) were generated and injected into the L4/5 DRG of adult male rats. Three weeks after DRG-AAV injection, tibial nerve injury (TNI) was induced and subsequent sensory behavior evaluation was performed weekly for an additional 5 weeks, after which tissues were harvested for IHC characterization of transgene expression. Results (Supplemental Figure 9) showed that AAV6-Na_V_iPA1 injection reduced TNI-induced mechanical and cold sensitization. IHC revealed efficient Na_V_iPA1 (fused to GFP) expression in DRG neurons and their peripheral (cutaneous) and central terminals (SDH). These data indicate that sustained expression of the Na_V_iPA1 selectively in the PSNs of the pathological DRG after TNI prevented development of pain behaviors.

### Treatment of established neuropathic pain by DRG-AAV6-NaViPA1 in male rats

**Figure 7:**
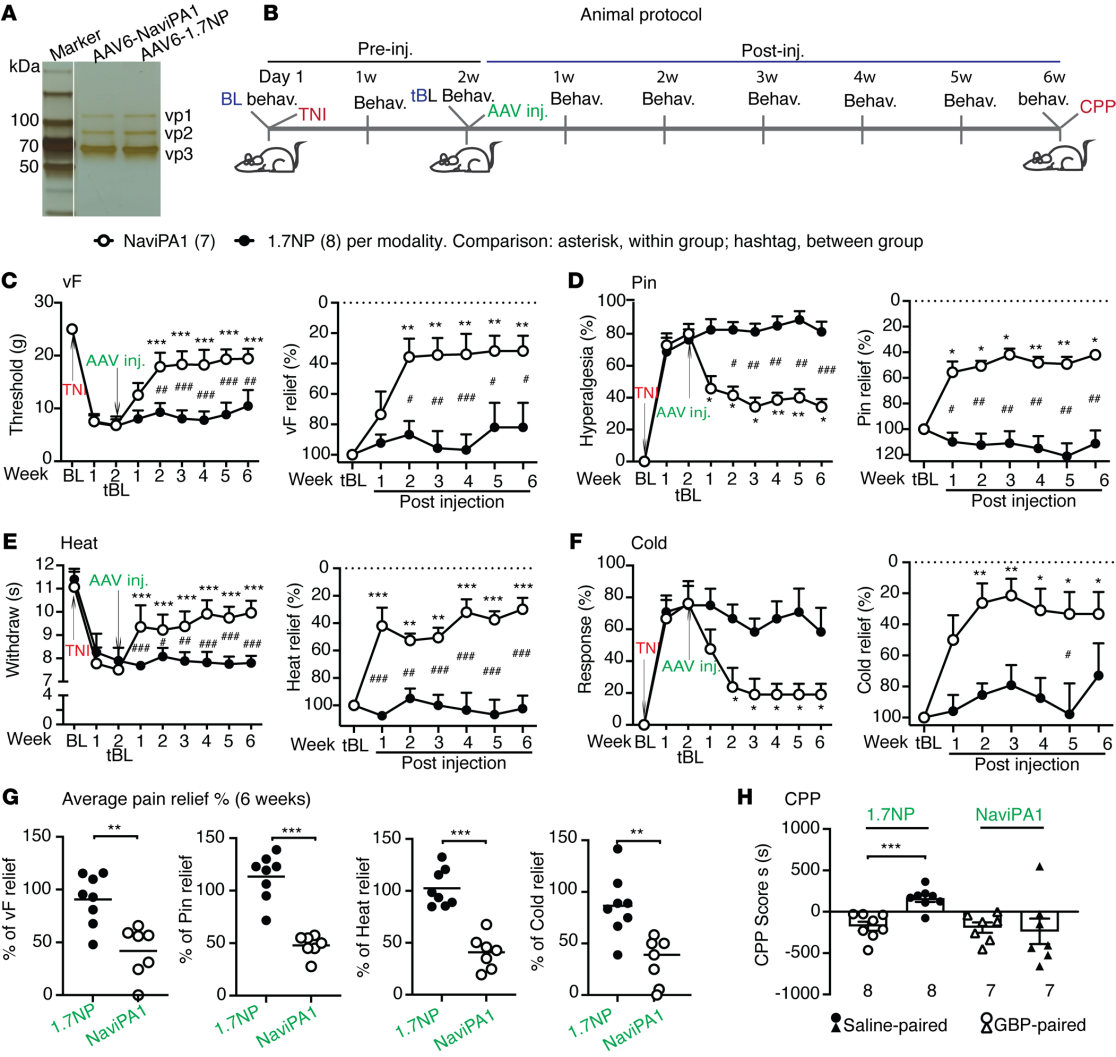
Treatment of established neuropathic pain by DRG AAV6-Na_V_iPA1 in male rats. (**A**) Silver stain of purified AAVs (a vertical white line denotes that the lanes were run on the same gel but were noncontiguous) were prepared for the experiment in an animal protocol schematically outlined in panel **B**. (**C**–**F**) The time courses (graphs on the left) of vF (**C**), Pin (**D)**, Heat (**E**), and (**F**)Cold before and after DRG injection of either AAV6-Na_V_iPA1 (*n =* 7) or AAV6-NP (control, *n =* 8). The measures on the 14th day after TNI and before AAV treatment (tBL) were converted as the peak pain intensity (100%), and the measures of each sensory modality after treatment were normalized to the measures at the tBL and the percentage of pain relief for each modality at multiple time points was calculated (graphs on the right). *P < 0.05, **P < 0.01 and ***P < 0.001 for comparisons to the tBL within group and ^#^*P* < 0.05, ^##^*P* < 0.01, and ^###^*P* < 0.001 between groups. Repeated measures 2-way ANOVA for vF and Heat, and Tukey’s (within group) and Bonferroni’s (between groups) post hoc; and nonparametric Friedman ANOVA for Pin and Cold tests and Dunn’s post hoc. Summed average pain relief in the 6-week treatment course showed 52%, 49%, 69%, and 67% reduction of vF-, Pin-, Cold-, and Heat**-**stimulated mechanical and thermal pain behaviors, respectively (**G**). ** *P* < 0.01 *** *P* < 0.001, unpaired, 2-tailed student**’**s *t* test. (**H**) Results of CPP scores (seconds, s) of preconditioning chamber and of the GBP-paired chamber between AAV-Na_V_iPA1 (*n =* 7) and AAV-NP (control, *n =* 8), ***P < 0.001 (unpaired, 2-tailed Student**’**s *t* test).

We next extended experiments to evaluate the effectiveness of DRG-AAV6-Na_V_iPA1 in a more clinically relevant design for reversal of established pain behaviors, including both evoked responses as well as spontaneous ongoing pain following TNI. In the experimental design, the sensitivity to mechanical and thermal cutaneous stimulation was assessed at baseline and weekly after TNI for 2 weeks before AAV injection. Thereafter, rats were randomized to receive DRG injection of either AAV6-Na_V_iPA1 or control AAV6-NP into the L4/L5 DRG ipsilateral to TNI, after which sensory behaviors were evaluated weekly for additional 6 weeks. As a terminal experiment, Gabapentin-induced (GBP-induced, 100 mg/kg, i.p.) conditioned place preference (CPP) test was performed in both groups to evaluate spontaneous pain (12, 44). Behavior measures before AAV injection on the 14th day after TNI were used as a treatment baseline (tBL) to evaluate effectiveness of vector treatments (Figure 7, A and B). Tissues were harvested for IHC characterization of transgene and target gene expression and for whole-cell current-clamp of neuronal excitability on dissociated DRG neurons.

All rats developed multiple modalities of pain behaviors 2 weeks after TNI, including lowered threshold for withdrawal from mild mechanical stimuli using calibrated monofilaments (von Frey test, vF), more frequent hyperalgesic-type responses after noxious mechanical stimulation, by applying a 22 g spinal anesthesia needle to the plantar surface of the hind paw with enough force to indent, but not puncture the skin (Pin test), and hypersensitivity to heat and acetone stimulation. These behaviors persisted after injection of the control AAV6-NP during the 6 weeks of observation course. In contrast, rats injected with AAV6-Na_V_iPA1 showed a gradual reversal of these changes, which were maintained throughout and predicted to outlast the observation period (Figure 7, C–F). For our protocol of treating existing pain, we converted the measures on the 14th day after TNI and before AAV treatment (tBL) as the peak pain intensity (100%), and the measures of each sensory modality after treatment were normalized to the measures at the tBL and the percentage of pain relief for each modality at multiple time points was calculated (Figure 7, C–F). Summed average pain relief in the 6-week treatment course showed 52%, 49%, 69%, and 67% reduction of vF-, Pin-, Cold-, and Heat-stimulated mechanical and thermal pain behaviors, respectively (Figure 7G). Using a biased CPP paradigm (45), the effect of AAV-Na_V_iPA1 treatment on the affective aspect of spontaneous pain was evaluated. None of the animals in either group were excluded from study because of their baseline preference/avoidance for a chamber (45). A significant GBP-induced CPP effect was observed in the TNI rats injected with AAV6-NP, indicating ongoing pain, while there was no significant difference in the time spent in the initially nonpreferred chamber during baseline versus testing periods in AAV-Na_V_iPA1–treated TNI animals, indicating that AAV-Na_V_iPA1 treatment significantly relieved on going spontaneous pain (Figure 7H).

**Figure 8:**
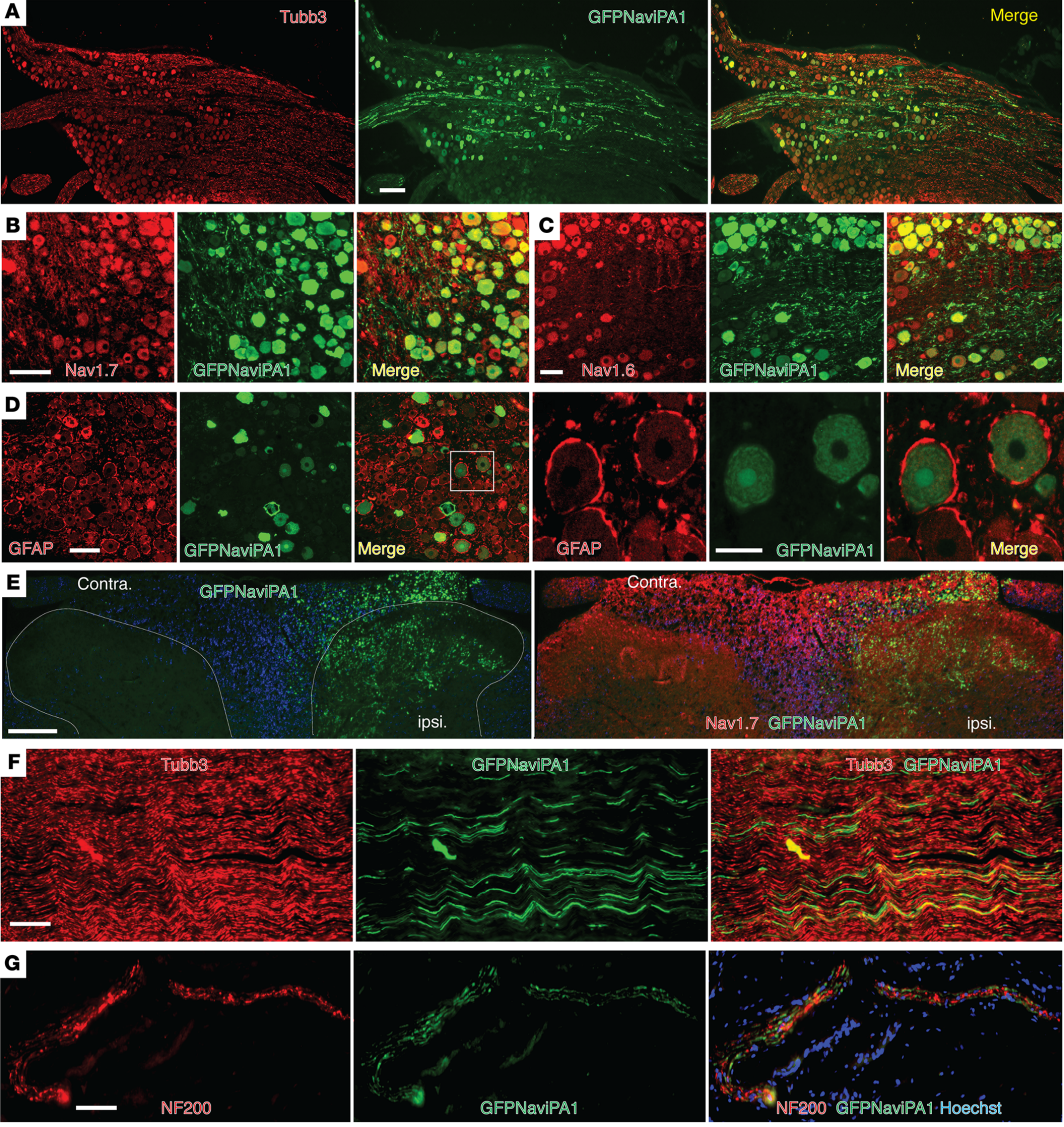
IHC of GFP-Na_V_iPA1 and target gene expression. (**A**–**D**) Representative IHC montage images (GFPNa_V_iPA1 with Tubb3) show neuronal expression profile 6 weeks after AAV- Na_V_iPA1 injection in TNI rats (**A**; Scale bar: 200 μm), colocalization of GFP-Na_V_iPA1 with Na_V_1.7 and Na_V_1.6-positive neurons (**B** and **C**; Scale bar: 100 μm), but not with GFAP positive perineuronal glia (**D**; Scale bar: 100 μm)**.** The square region was enlarged and montage images shown as the 4th image in row D). (**E**–**G**) Representative IHC montage images illustrate GFPNa_V_iPA1 (green) and Na_V_1.7 (red) in PSN central terminals of ipsilateral spinal dorsal horn (**E**; Scale bar: 200 μm), GFPNa_V_iPA1 (green) and Tubb3 (red) in sciatic nerve (**F**; Scale bar: 50 μm), and GFPNa_V_iPA1 (green) and NF200 (red) in PSN peripheral terminals of skin section (**G**; Scale bar: 50 μm).

Histological examination (Figure 8) determined the in vivo transduction rate for AAV6-Na_V_iPA1 in the 6th week after vector injection. The Na_V_iPA1-positive neurons (GFP) comprised 37% ± 4 % (1,283 out of 3,447 total neuronal profiles) identified by a panneuronal marker β3-tubulin (*n =* 4 DRG, 3–4 sections per DRG, selected as every 5th section from the consecutive serial sections). Transduced DRG neurons included the full-size range of the PSNs that also expressed Na_V_1.7 and Na_V_1.6, and expression showed multiple subcellular localizations, preferably in PSN cytosol. Positive GFP signals were not detected in GFAP-positive perineuronal glial cells. GFP signals were also detected in the ipsilateral dorsal horn, sciatic nerve, and cutaneous afferent terminals.

These findings together demonstrate that DRG injection of AAV6-encoded Na_V_iPA1 induced Na_V_iPA1 expression restricted to the PSNs of injected DRG and their peripheral and central processes. This strategy via AAV6-mediated expression of Na_V_iPA1 selective in the sensory neurons of the anatomically segmental DRG responsible for pain pathophysiology has clear analgesic effectiveness in normalizing the established peripheral hypersensitivity for both evoked and spontaneous pain behavior in the rat model of peripheral injury–induced neuropathy.

### Reversal of PSN hyperexcitability by AAV6-NaViPA1 treatment in male rats

**Figure 9:**
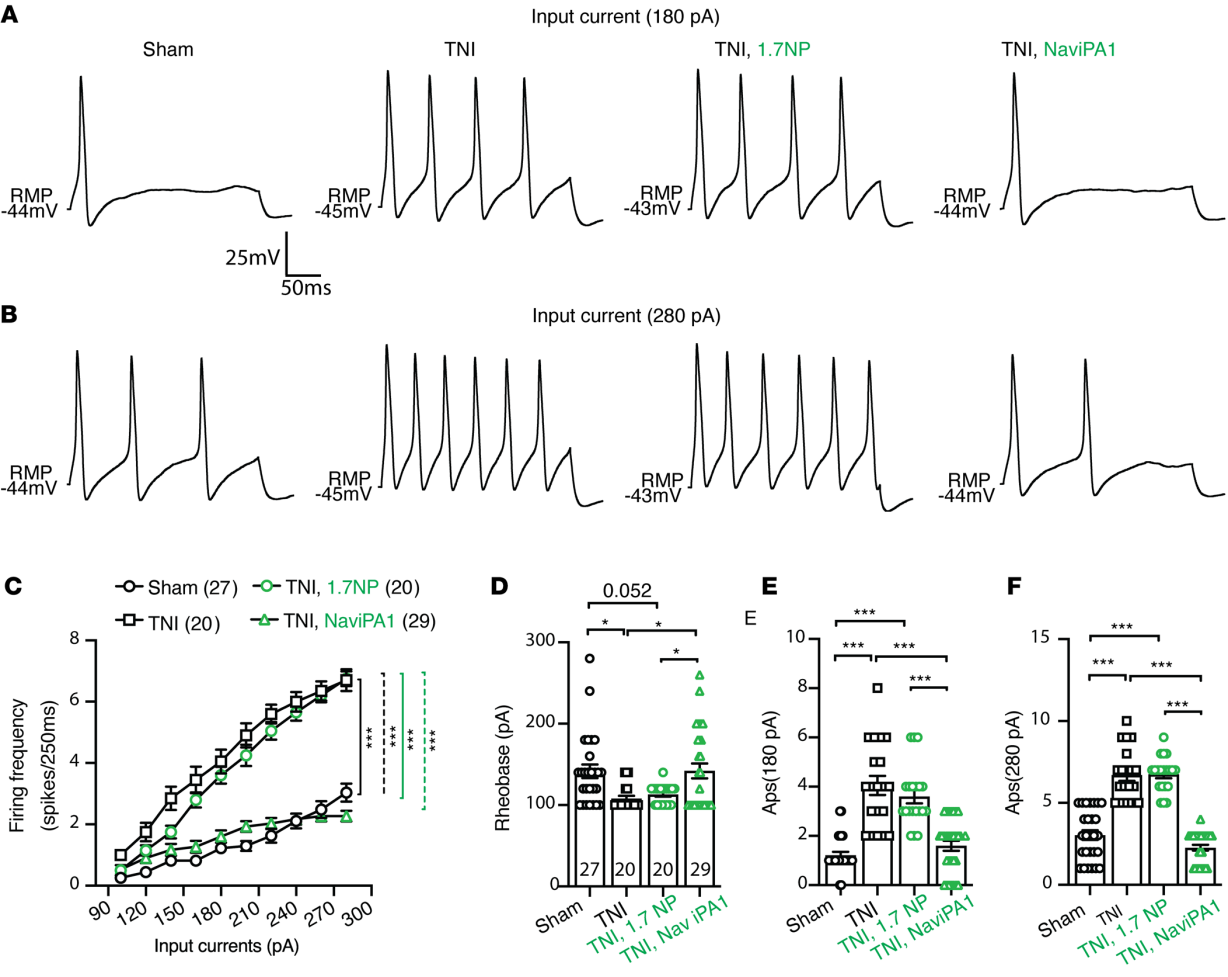
Na_V_iPA1 expression on neuronal excitability of male rat PSNs. (**A** and **B**) Representative AP traces elicited by 250 ms depolarizing current of 180 pA (**A**) and 280 pA (**B**) (same cells) from RMP were recorded from DRG neurons dissociated from the rats of sham, TNI only, and GFP-expressing neurons in TNI treated with AAV6-NP or AAV6-Na_V_iPA1, as indicated. (**C**) Comparison of responses (number of APs evoked by a 250 ms stimulus) for the populations of DRG neurons in different groups across a range of step current injections from 100 to 280 pA; ***P < 0.001, 2-way ANOVA of main effects of groups with Bonferroni’s post hoc. Scatter plots with bars show analysis of the (**D**) rheobases and (**E** and **F**) AP numbers evoked by input current at (**E**) 180 pA and (**F**) 280 pA from RMP. The number in each group is the number of analyzed neurons per group. *P < 0.05 and ***P < 0.001, 1-way ANOVA and Turkey’s post hoc.

Increased excitability of nociceptive PSNs is a fundamental process underlying neuropathic pain (46). We therefore examined whether AAV6-Na_V_iPA1 treatment reverses the enhanced neuronal excitability of nociceptive PSNs following TNI (12, 47) using the whole-cell current-clamp AP recording of DRG dissociated neurons from rats after the treatment protocol shown in Figure 7B. Although TNI results in DRG containing injured and uninjured neurons, nerve injury can induce an increase of voltage-gated ion channel activity in both axotomized neurons and adjacent intact neurons, leading to similar electrophysiological (EP) changes and increased discharge frequency in axotomized and neighboring intact DRG neurons (48, 49), possibly through interneuronal signaling and coupling (50). We therefore performed current-clamp recordings from randomly chosen small-to-medium-sized neurons (under 35 μm in diameter) (51) in the cultures from dissociated L4 and L5 DRG. Transduced neurons were identified by GFP fluorescence, and excitability was evaluated by measuring rheobase and repetitive action potential (AP) firing during 250 ms current pulses stepping from 100 pA and 280 pA current injection. Results showed that the averaged rheobase in the neurons from TNI rats was significantly decreased and, in response to a step stimulus, the frequency of APs evoked in neurons from TNI rats was significantly increased compared with sham controls. These were normalized in the transduced neurons after AAV6-Na_V_iPA1 treatment, whereas NP-transduced neurons had no significant effects (Figure 9). These findings indicate that reversal of nerve injury–induced sensory neuronal hyperexcitability by Na_V_iPA1 may contribute to its analgesic effects in attenuation of neuropathic pain behaviors, i.e., conduction block of TTXs Na_V_ ion channels selectively in PSNs leads to a decrease in neural excitability, resulting in mitigation of pain behaviors.

### Analgesia of DRG-AAV6-NaViPA1 treatment in female TNI rats

**Figure 10:**
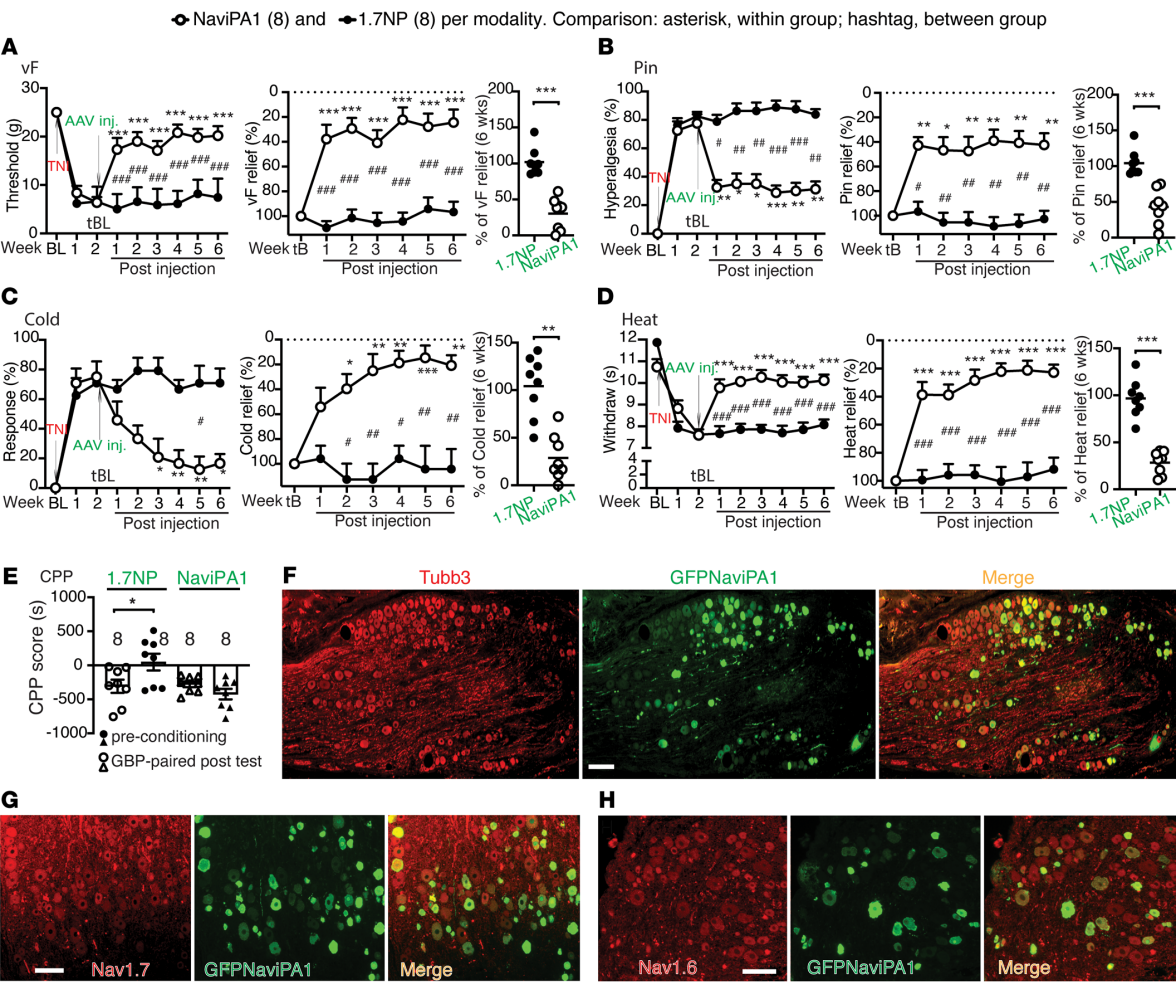
Analgesia of DRG-AAV6-Na_V_iPA1 treatment in female TNI rats. (**A**–**D**) Analogous figures to those shown in Figure 7 show significant analgesia (left graphs) and % of pain reduction (middle graphs) after DRG delivery of AAV6-Na_V_iPA1 in the established TNI pain behaviors of female rats. *P < 0.05, **P < 0.01 and ***P < 0.001 for comparisons to the treatment baseline (tBL) within group and ^#^*P* < 0.05, ^##^*P* < 0.01, and ^###^*P* < 0.001 for comparisons between groups. Repeated measures parametric 2-way ANOVA for vF and Heat followed by Tukey’s (within group) and Bonferroni’s (between groups) post hoc; and nonparametric Friedman ANOVA for Pin and Cold tests and Dunn’s post hoc. Right graphs of **A**–**D** show average pain relief of each modality in 3.5-month treatment, ** *P* < 0.01 and *** *P* < 0.001 comparisons between groups (unpaired, 2-tailed Student**’**s *t* tests). (**E**) CPP difference scores (s) of preconditioning chamber and of the GBP-paired chamber between AAV- Na_V_iPA1 (*n =* 8) and AAV-NP (control, *n =* 8), *P < 0.01 (unpaired, 2-tailed Student**’**s *t* test). (**F**–**H**) Representative montage IHC images colocalization of GFP-Na_V_iPA1 with Tubb3 (**F**), Na_V_1.7 (**G**), and Na_V_1.6 (**H**) 6 weeks after AAV-Na_V_iPA1 injection. Scale bar: 100 μm.

Sex differences exist in experimental and clinical pain and in responsivity to interventions (52). We therefore next tested whether DRG-AAV6-Na_V_iPA1 treatment is also effective in attenuating hypersensitivity induced by TNI in female animals, using the protocol similar to the tests in male animals (Figure 7). The same batch preparation of AAV6-Na_V_iPA1 and AAV6-NP tested in male rats was used for injection. Results showed that the female rats displayed similar phenotypic development of hypersensitivity after induction of TNI to male rats and that both evoked mechanical/thermal hypersensitivity and GBP-CPP responses were normalized after AAV6-Na_V_iPA1 treatment, demonstrating comparable analgesic effects (Figure 10, A–E) with the male animals. IHC on the DRG sections from female TNI rats 6 weeks after AAV6-Na_V_iPA1 injection also revealed GFP-Na_V_iPA1 expression profile comparable with male rats (Figure 10, F–H ), and the in vivo transduction rate was 39% ± 8 % (766 out of 1,983 total Tubb3-positive neuronal profiles). Thus, although not rigorously compared, treatment effects were comparably concordant between the sexes, suggesting that sexual dimorphism seems not apparent for both pain behavior phenotypes after TNI and in response to DRG-AAV6-Na_V_iPA1 treatment in our studies (12).

## Discussion

Sustained peripherally targeted analgesia without risk of addiction is a global unmet medical need (53–55). Na_V_1.7 is currently a leading target for analgesic pharmaceutics. However, ample evidence demonstrates that multiple sensory neuronal Na_V_s contribute to nociceptive electrogenesis and pain pathogenesis (15, 56). Here, we reported that targeting Na_V_-IDRs facilitated the discovery of Na_V_iPAs. A prototype, Na_V_iPA1, initially derived from Na_V_1.7, is highly conserved in sequences among TTXs Na_V_s. Accordingly, it demonstrated a similar multipronged inhibitory characteristic to TTXs I_Na_ conducted by Na_V_1.7, Na_V_1.6, Na_V_1.3, and Na_V_1.1, but no effect on TTXr I_Na_ conducted by Na_V_1.8 and Na_V_1.5. Na_V_iPA1 expression in DRG-PSNs produced selective inhibition of TTXs I_Na_ but not TTXr I_Na_. DRG delivery of AAV6-encoded Na_V_iPA1 significantly attenuated established nerve injury-induced pain behaviors in male and female animals for both evoked mechanical and thermal hypersensitivity and ongoing or spontaneous pain behaviors, the symptoms commonly found in patients suffering from multiple types of painful neuropathy (57). Additionally, blockade effects of TTXs I_Na_ by Na_V_iPA1 were replicated in the hiPSC-SNs, supporting a translational potential. Because several different types of Na_V_s in sensory neurons combine to trigger nociceptor electrogenesis required for AP trains (1), block of several of these specific in DRG-PSNs is conceived to be a therapeutical advantage for neuropathic pain.

Chronic pain in almost all cases is maintained by ongoing afferent hyperactivity originating from peripheral pathological sources (53, 58, 59). Thus, development of novel peripheral-acting strategies for pronociceptive Na_V_ inhibition in the PSNs would be an ideal approach for clinical pain treatment (2, 54). Our strategy described here includes an approach by which highly selective and nontoxic Na_V_iPA1 is designed and developed from Na_V_-IDRs, which is delivered by using AAV to the pathological DRG. PSN-restricted inhibition of multiple pronociceptive TTXs Na_VS_ is predicted to have advantages for DRG-targeted analgesia, as a recent expert commentary states that excitability of neurons is determined by several different Na_v_ channels, therefore targeting one alone may not be sufficient. They explain that this may correlate with the inadequate analgesic pharmaceutics that inhibit only Na_v_1.7 (60). It is known that individuals and animal models that are heterozygous for null mutations of Na_V_1.7 are normal in sensory phenotypes. Thus, AAV-mediated Na_V_iPA1 expression restricted in DRG-PSNs may induce analgesia via a combined partial inhibition of Na_V_1.7, Na_V_1.6, Na_V_1.3, and Na_V_1.1, while avoiding undesirable side effects otherwise due to global distribution of small molecule inhibitors. Although PSN somata in DRG are anatomically isolated from each other and are not synaptically interconnected, most DRG-PSNs are transiently depolarized when axons of neighboring neurons of the same ganglion are stimulated repetitively (61). This coupled activation occurs among various-sized neurons, including small-diameter nociceptors and large-diameter low-threshold mechanoreceptors (50). Therefore, although AAV produces incomplete sensory neuron transduction, transduced neurons can induce a reduction of pronociceptive ion channel activity in both transduced neurons and adjacent nontransduced neurons, leading to similar electrophysiological changes. Another possible advantage is that, unlike gene therapy strategies such as RNAi (62) and CRISPIR-dCas9 or ZEN epigenetic suppression (9) that irreversibly reduce the production of a target protein, which is potentially problematic (63); AAV-mediated Na_V_iPA1 expression selective in PSNs provides sustained and restricted blockade of electrogenesis on multiple TTXs Na_V_s without abrogating proteins per se, providing specific functional interference. A complete block of Na_V_1.7 activity is not intended since it may induce a state of total insensitivity to pain where unintended self injury would occur (64).

Pain-sensing PSNs can become hyperexcitable in response to peripheral nerve injury, which in turn leads to the development of neuropathic pain. Multiple lines of evidence from both preclinical and clinical studies demonstrate that block of peripheral nociceptive input can effectively relieve pain symptoms, including spontaneous pain (65, 66). Therefore, treatments targeting the peripheral PSNs both avoid CNS side effects and also are likely to succeed. Indeed, a recent expert commentary states that primary afferent neuronal activity is a promising target in the development of safe therapies for patients with chronic pain (53). Delivering drugs to the DRG is well developed and safe, for instance, as used by anesthesiologists for regional blockade and by pain physicians for diagnosis and treatment of radiculopathy (67). Injection into the DRG has minimal consequences in preclinical models (68). It has also been demonstrated that unintentional intraganglionic injection commonly accompanies clinical transforaminal epidural steroid injection (67), a very common procedure with minimal risk of nerve damage. Thus, the PSNs are particularly suitable for targeting new analgesic treatments, especially at the levels of associated pathological DRG (54, 69). A recent study reports that central nervous system gene therapy by intravenous high-dose AAV causes asymptomatic and self-limited DRG inflammation and mild PSN degeneration in primates (70). Since these changes are very minor in comparison with the those induced by painful and neuropathic conditions that AAV injection would treat, this is unlikely to become a barrier to the clinical application of our approach.

In preclinical models, direct DRG delivery of AAVs encoding analgesic biologics can provide relief in chronic pain, with high transduction efficiency, flexibility for selective segmental localization, and minimal behavior changes attributable to the surgical procedure (71). In parallel, injection techniques are being advanced to achieve minimal invasive delivery of biologics for future clinical pain therapy (72, 73). Small peptides derived from the target protein sequences can serve as decoy molecules to selectively interfere with the function of their target signaling proteins by preemptively binding to them (13). We have successfully employed this strategy in rat models to induce analgesia by block of T-type/Ca_V_3.2 channel functions (12) and by blocking membrane trafficking of Ca_V_2.2 channels via interruption of its interactions with the structural protein of collapsin response mediator protein 2 (CRMP2) (13). Here, we extend the applicability of DRG-AAV strategy to the analgesic effectiveness of multiple PSN TTXs Na_VS_ blockade for neuropathic pain. These encouraging results indicate efficacy and tolerability, if further validated for long-term efficacy and minimal side effects, and suggest the transformational potential of the approach for developing addiction-free peripheral pain therapeutic agents. Beyond peripheral nerve injury–induced pain, dysfunctional Na_V_s have been found in various pain conditions, such as osteoarthritis (OA), which is frequently highlighted as an unmet medical need. Thus, for pain conditions like OA, targeting the TTXs Na_V_s might be therapeutically useful (74, 75).

While our studies illustrate the power of rational analgesic peptide drug design strategy and provide encouraging results, we acknowledge several limitations in the current study. Different sodium channels traffic to distinct subcellular locations of PSNs (membrane, terminals, nodes of Ranvier, among others), and the regulation of this process may provide several options to control neuronal excitability in different pathophysiological contexts. Injury-induced peripheral hypersensitization associated with Na_V_ malfunction affects multiple sites of the peripheral sensory nervous system, including augmented pain perception in the peripheral terminals, enhanced nociceptive signal transduction in PSN soma and T-junction, and increased neurotransmission in the spinal dorsal horn. At this early stage, our studies did not investigate differential actions by block of TTXs Na_VS_ along the pathway of peripheral nociceptors, nor did the results rule out the possibility that block of TTXs Na_V_s reduces pain by inhibiting afferent hyperexcitable input (76), thus indirectly modulating spinal cord and brain antinociceptive control circuits. Another limitation is that the molecular mechanism of Na_V_iPA1 functioning remains incompletely delineated. Our study has verified lack of pleiotropic effects on big potassium (BK) and calcium channels, but we cannot rule out the possibility of peptide interaction with other unknown targets that mediate protein binding. Theoretically, if the peptide binds to membrane via a lipid mechanism, it might mediate the PM, targeting of a wide array of proteins carrying specialized domains enriched with positive charges. Delineation of the mechanisms in sensory neurons in future investigation is critical for the assessment of therapeutic efficacy and potential side effects.

Although we have shown that polybasic NLS and multiple adjacent serine residues are required for Na_V_iPA1 function, phosphorylation-dependent binding of NaviPA1 to the membrane appears unlikely to be essential because serine phosphorylation will neutralize the positive charge of NaviPA1. It has been reported that polybasic peptide with nonphosphorylatable serine shows strong membrane binding (77), and highly polar neutral-serine bearing a hydroxyl group at the terminal carbon offers a stronger interaction with the lipid bilayer membranes (78). Other types of PTMs in the residues of Na_V_iPA1 sequence may also play roles. It is reported that serine PTMs can occur by diverse mechanisms, including phosphorylation, sulfation, acetylation, palmitoylation, myristoylation, and glycosylation (79–81). Different PTMs can alter the charge and hydrophobicity (electrostatics), which, in turn, induce physicochemical properties, structure, and functional changes of the peptide. Ion channel protein arginine methylation and lysine acetylation can enhance current density by increasing the channel cell surface expression (82, 83). A recent paper reported that alanine substitution of polybasic arginine/lysine in Na_V_1.7iPA region in Halo-tagged human full-length Na_V_1.7 does not alter the membrane integration and channel function of Halo-Na_V_1.7 after transfection (84). It would be interesting to test whether combined mutations of polybasic arginine/lysine and multiple adjacent serine or other conserved residues would change the natural full-length Na_V_1.7 polar association that will influence channel function. Additionally, the highly disordered Na_V_iPA1 liberated by engineering from full-length Na_V_1.7 protein likely renders the Na_V_iPA1 different biological properties, such as binding to membrane, probably via electrostatic interactions, and showing an ability for cell nuclear trafficking (12). A possibility that cannot be dismissed is that the nuclear-entry Na_V_iPA1 functions as a transcriptional factor that affects the genes that are critical in regulating Na_V_1.7 functions, reminiscent of the fragmented L-type calcium channel functioning as a transcription factor (85, 86). It is also possible that Na_V_iPA1 may function as a decoy peptide that interrupts Nav1.7 interactions with partners, since Na_V_iPA1, which is partially aligned to a putative Na_V_1.7 dimerization sequence, may affect channel functions by uncoupling Na_V_1.7 dimerization assembly (87, 88), albeit experimental evidence of such a mechanism remains to be shown. The potential signaling pathways that the Na_V_iPA1 affected could be many, since Na_V_1.7 PPI molecule networks involve multiple pathways and Na_V_1.7 (and other TTXs Na_V_s) intracellular segments serve as essential interfaces for many regulatory signaling molecules, including protein-lipids interactions (35, 36). Alterations of these molecules following nerve injury are essential for ectopic PSN hyperactivity and pain. Future work will address these questions.

## Methods

### Sex as a biological variable.

Since sex differences exist in experimental and clinical pain and in the responsivity to interventions (52), pain hypersensitivity after TNI and pain reversal responses to treatment were examined in both male and female rats for this study. All Materials and Methods are presented in the Supplemental Methods.

### Statistics.

Statistical analysis was performed with GraphPad PRISM 9 (GraphPad Software). The methods were detailed in the figure legends and results were reported as the mean and SEM. Differences were significant for values at *P* < 0.05. For comparisons between groups, in the pilot in vivo testing of TNI operation at 3 weeks after AAV intraganglionic injection, the effects of vector injection were characterized by treatment area under the curve (tAUC) analysis; in the treatment protocol of established pain, the measures immediately before AAV injection at the 14th day after TNI were used as the tBL for calculating tAUC. In the treatment of established male TNI pain, the data points in a rat who died on the second day after treatment AAV injection (no diagnostic report and likely due to surgical injury) were excluded from the analysis.

### Study approval.

All animal experiments were performed with the approval of the Medical College of Wisconsin Institutional Animal Care and Use Committee (AUA00007371) in accordance with the National Institutes of Health Guidelines for the Care and Use of Laboratory Animals. The uses of AAV and human iPSC sensory neurons were approved by the Medical College of Wisconsin Institutional Biosafety Committee, with approval numbers IBC20140322 and IBC20220103.

### Data availability.

The raw data, analytic methods, and study materials are described in full in the Supplemental Methods. Values for all data points in graphs of the manuscript and supplemental materials are reported in the Supporting Data Values file. All gel data and IBs in this study are reported in the full unedited gel file available in the supplement with full annotations. Data for the manuscript and supplemental materials including plasmid nucleotide sequences (text) of pAAV-CBA-GFPNaviPA1 and pCMV-cDNA3.1(+)-hSCN10A-FurinP2A-hSCN2B with annotations of key components of the constructs are findable for the research community through Dataverse at dataverse.harvard.edu using identifier UXVAPW, or through the link https://dataverse.harvard.edu/dataset.xhtml?persistentId=doi:10.7910/DVN/UXVAPW

## Author contributions

HY designed the study and wrote the manuscript. HY, QHH, and TRC reviewed and revised the manuscript. SMS, BIZ, FF, YX, CQ, and HY performed experiments, analyzed data, and organized all figures. QHH supervised DRG injection. HY and QHH obtained funding. TRC provided HEK1.7 stable cell line and consulted EP experiments. All authors approved the final version.

## Supplementary Material

Supplemental data

Unedited blot and gel images

Supporting data values

## Figures and Tables

**Table 1:**
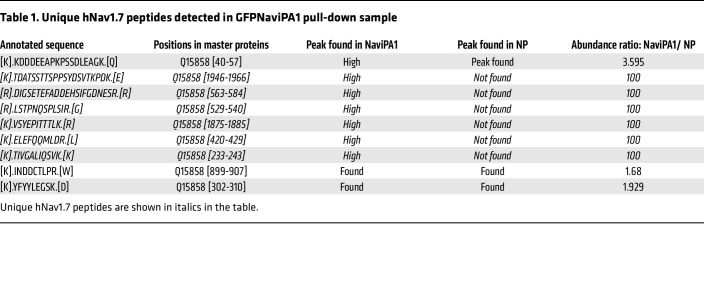
Unique hNav1.7 peptides detected in GFPNaviPA1 pull-down sample

## Version 1 05/09/2024

In-Press Preview

## Version 2 07/01/2024

Electronic publication
