# Supplementary material for: Peripherally targeted analgesia via AAV-mediated sensory neuron–specific inhibition of multiple pronociceptive sodium channels
Source: J Clin Invest. 2024 May 9;134(13):e170813. doi: 10.1172/JCI170813 (PMC11213509; doi:10.1172/JCI170813)
Supplement: Supplemental data [file jci-134-170813-s157.pdf]

Supplementary data

METHODS

Sex as a biological variable: Since sex differences exist in experimental and clinical pain and in the responsivity to interventions (1), pain hypersensitivity after TNI and pain reversal responses to treatment were examined in both male and female rats for this study.

Animals

Adult male and female Sprague Dawley (SD) rats weighing 100-125g body weight (Charles River Laboratories, Wilmington, MA) were used. Animals were housed individually in a room maintained at constant temperature (22±0.5°C) and relative humidity (60±15%) with an alternating 12h light-dark cycle and were access to water and food ad libitum throughout the experiment. All efforts were made to minimize suffering, and all survival surgeries were completed in a sterile environment under a surgical microscope in animals anesthetized with isoflurane (2–5%). For tissue harvest euthanasia, animals were deeply anesthetized by isoflurane followed by decapitation with a well-maintained guillotine. The estimated numbers of animals needed were derived from our previous experience with similar experiments (2) and a power analysis was not performed. The numbers of rats used were detailed in the relevant sections or figure legends of the experiments.

Computational (in silico) designs

Rat full-length $Na_V1.7$ aa sequence was retrieved from the UniProt KB knowledge database (UniProt Knowledgebase release 2018_11). $Na_V1.7$ protein TM domains and intracellular termini and loops were predicted by Phobius (3). The $Na_V1.7$ protein IDRs were predicted by analyzing the full-length $Na_V1.7$ sequence using DEPICTER (4). Potential phosphorylation sites in the

Na_V1.7 full aa sequence were identified using Disorder Enhanced Phosphorylation Predictor (DEPP) (5). Potentially functional peptides within the IDRs were further analyzed SLiMPrints, which predict short linear motifs (SLiMs) based on strongly conserved SLiMs within IDRs (6). Peptide structure determination was analyzed by I-TASSER (7). MacVector ClustaIW (MacVector, Apex, NC) was used for vector designs and sequence alignments.

Molecular cloning and AAV constructs

The construct pAAV-CBA-GFP-1.7iPAs encode the GFP-Na_V1.7iPA fusion protein downstream a chimeric intron for enhancing transcription, driven by a hybrid CMV enhancer/chicken β-actin (CBA) promoter, and a mRNA stabilizing Woodchuck Posttranscriptional Regulatory Element (WPRE) sequence was inserted downstream of stop code of GFP-Nav1.7iPAs and upstream of human growth hormone poly A signals. Plasmids were subsequently used in transfection experiments and in AAV vector generation. To package AAV6-GFP-1.7iPA1, AAV6-GFPlinker, and AAV2/6-GFP-NP (a Na_V1.7 N-terminal inert peptide) (referred to as AAV6-Na_ViPA1, AAV6-GFP, and AAV6-NP, respectively) for in vivo injection. AAV vectors were produced and purified in our laboratory by previously established methods (8). The titers (GC/mL) of AAV6-GFP), AAV6-Na_ViPA1, and AAV6-NP vectors were 2.45 $x10^{13}$, 3.05 $x10^{13}$, 2.64 $x10^{13}$, respectively. Same batches of AAVs were used in all in vivo experiments.

Site-directed mutagenesis

Alanine substitution for arginine/lysine and serine on pAAV-CBA-GFPNaviPA1 to generate NaviPA1mt1-6 was made using the QuikChange mutagenesis method (Stratagene). All constructs were verified by DNA sequencing before use.

Supplementary data

Cell culture

Cell lines. HEK293 cell line stably expressing human wide-type $Na_V1.7$ (HEK1.7) was provided by Dr. Theodore Cummins (Indiana University - Purdue University, Indianapolis). HEK293 cell lines stably expressing human wide-type $Na_V1.6$ (HEK1.6), $Na_V1.3$ (HEK1.3), $Na_V1.1$ (HEK1.1), $Na_V1.5$ (HEK1.5), and CHO cells stable expression of human $Na_V1.8$ (CHO1.8) were obtained from Charles River. Neuronal NG108-15 (NG108) neuronal-like cells and F11 cells (hybrid cells of mouse neuroblastoma cells with embryonic rat DRG neurons) were purchased from ATCC (Manassas, VA). ND7/23 neuronal cells were obtained from Sigma-Aldrich. Rat DRG-neuronal 50B11 cells (50B11) were used as reported previously (9). These cells were cultured and transfected (PEI 40) using standard techniques, as described previously (2).

Generation of human $Na_V1.8$ stable HEK293 cells. To generate $Na_V1.8$ stable expression HEK293 cells (HEK1.8 cells), an expression plasmid of pcDNA3.1(+)-SCN10A-Furin-P2A-SCN2B was constructed (Genscript) in which a CMV promoter transcribes human $Na_V1.8\alpha$ and $Na\beta2$ from a single open reading frame (ORF) expressing human SCN10A (NM_001293306.2) and SCN2B (NM_004588.5) separately linked by a 2A self-processing sequence derived from porcine teschovirus-1 (P2A) and a furin cleavage site (**Supplemental Figure 2**) (10). Final construct was sequence confirmed and transfected in HEK293 cells, and the cells were frequently selected with G-418 (800 μg/mL), followed by establishment of single cell colonies using BIOCHIPS Single-cell Isolation Chip (ThermoFisher) according to the manufacturer recommended protocol. $Na_V1.8\alpha$ and $Nav\beta2$ expression were determined by immunoblots using

cell lysates and extracted cell cytosols and membranes, and prioritized by functional sodium current amplitude using conventional whole-cell voltage-clamp (see further).

Dissociated DRG neuronal culture. Dissociated DRG neuronal cultures for EP were performed, as described previously (11) and were studied in 6~8 h after harvest in EP experiments.

Human induced pluripotent stem cells (iPSC)-derived sensory neurons (hiPSC-SNs). HiPSC-SNs, which were derived from female human ectoderm-neural crest stem cells, and ChronoTM Senso-MM complete growth medium were purchased from Anatomic (Minneapolis, MN) (12). HiPSC-SNs maturing differentiation culture was performed per manufacturers' recommendation.

Generation of lentivector expressing Na_ViPA1 and NP for hiPSC-SNs transduction

Lentiviral (LV) expression plasmid pWPT-GFP (9) was used to express Na_ViPA1 and NP (control) (**Supplemental Figure 6**). LV were packaged using pWPT-GFPNa_ViPA1 and pWPT-GFPNP with packaging plasmid pCMVdR8.74 and envelop plasmid pVSV-g, concentrated, and products titrated in the range of 1x10^8 to 2x10^8 transduction unit/mL. Cultured hiPSC-SNs were infected by LV-GFP, GFPNa_ViPA1 or LV-GFPNP in the presence of 8□g of polybrene (Sigma-Aldrich, St. Louis, MO) per mL at an optimized multiplicity of infection (MOI) ≈5.

Electrophysiology (EP)

EP recordings were performed, as we described previously with minor modifications at room temperature (22~25°C) (11, 13), in a blind manner where the electrophysiologist was not aware of the treatment. Patch pipettes 0.9-2.5MΩ resistance were formed from borosilicate glass (King

Precision Glass Co., Claremont, CA) and fire polished. Recordings were made with an Axopatch 700B amplifier (Molecular Devices, Downingtown, PA). Signals were filtered at 5 kHz and sampled at 20 and 50 kHz (adopted from literature) (14-23) with a Digidata 1440A digitizer and pClamp10 software (Molecular Devices, San Jose, CA). Series resistance (3–5MΩ) was monitored before and after the recordings, and data were discarded if the resistance changed by 20%. After achieving the whole-cell recording, capacitance (Cm) and series resistance (Rs) were compensated accordingly.

Sodium channel current (I_{Na}) recording in cultured cell lines. Whole-cell voltage-clamp to recording I_{Na} was performed in HEK1.7, HEK1.1, HEK1.3, HEK1.6, HEK1.5, HEK1.8, CHO1.8, NG108-15 cells, and F11cells in current-density (I-V) and fast-inactivation voltage protocols. External solution consists of the following (in mM): 110 NaCl, 20 tetraethylammonium-Cl, 0.01 $CaCl_2$, 0.1 $CaCl_2$, 5 $MgCl_2$, 10 HEPES and 5.56 mM glucose (pH 7.4, 310–315 mosM/L). The internal pipette solution consisted of (in mM): 10 NaCl, 130 CsCl, 5 $MgCl_2$, 5 EGTA, 2.5 Na^{2+}ATP and 10 HEPES (pH 7.2). After formation of a tight seal (maximal leak amplitude <150 pA), membrane resistance and capacitance were determined. The voltage dependence of activation was assessed from holding potential using 50 ms pulses (test-pulse) to a range of test potentials from −100 mV to +50 mV in 5 mV or 10 mV incremental steps with an interval of 5 s. Current density was calculated by normalizing maximal peak currents with cell capacitance. The voltage dependence of steady-state fast inactivation was measured using a two-step protocol. A 500 ms pre-pulse with various potentials ranging from V_{hold} to +10 mV in 5 or 10 mV incremental steps was used to inactivate the channels. This pre-pulse was immediately followed by a 40 ms test-pulse at 0 mV to determine the remaining fraction of available channels. Inward current measured

during the test-pulse to 0 mV was normalized to the cell's maximum test-pulse inward current. To determine the conductance-voltage (I–V) relationships of voltage-dependent activation, the peak current densities during each voltage command step were fitted to a smooth curve with a Boltzmann equation: $I = G_{max}(V - E_{rev})/(1 + \exp([V - V_{50}]/k))$, which provided the maximum conductance (G_{max}). Normalized activation curves were fitted with a Boltzmann equation $G/G_{max}=1/(1+\exp([V-V_{50}]/k))$, where G was calculated as follows: $G=I/(V-E_{rev})$. The steady-state inactivation curves were fitted with $I/I_{max} = 1/(1 + \exp([V - V_{50}]/k))$. In all the equations, V_{50} denotes the half-activation and half inactivation potentials, Vm is the membrane potential, Erev is the reversal potential, k is the slope factor, G is the conductance, and I is the current at a given Vm; G_{max} and I_{max} are the maximum conductance and current, respectively. Current density was obtained by dividing the maximum peak current (pA) by the cell capacitance (pF). Voltage errors defined by Rs x Imax were minimized by using 80-85% series resistance compensation, and the compensation was readjusted before each voltage-clamp protocol. Cells were excluded if the voltage error exceeded 5mV. The cDNA construct encoding the human Nav1.8 was subcloned into a pcDNA3.1 expression vector and co-transfected with plasmids coding GFPNaviPA1 or GFP1.7NP (control) into ND7/23 cells and $I_{Na1.8}$ was recorded as described previously (24).

TTXs and TTXr I_{Na} recording in DRG dissociated neurons (male rats) and hiPSC-SNs. Isolated I_{Na} was recorded from single small/medium DRG neurons (≤35 μm in diameter, 4wk after AAV-DRG injection in naïve rats) and differentiated hiPSC-SNs in bath solution that contained the following (in mM): 80 NaCl, 50 choline-Cl, 30 TEA-Cl, 2 CaCl2, 0.2 CdCl2, 10 HEPES, and 5 glucose, pH 7.3 with NaOH. Internal solution containing the following (in mM): 70 CsCl, 30 NaCl, 30 TEA-Cl, 10 EGTA, 1 CaCl2, 2 MgCl2, 2 Na2ATP, 0.05 GTP, 10 HEPES, and 5 glucose,

pH 7.3 with CsOH. A voltage protocol was adopted to separate TTXr I_{Na} and TTXs I_{Na} (15, 25). In brief, a 500 ms prepulse to -120 or -50 mV was applied before a 50 ms test pulse from -100 to 40 mV with steps of 5 or 10 mV by test pulses from -50 to 0 mV. Both TTXs and TTXr I_{Na} were apparent after the -120 mV prepulse; only TTX-R I_{Na} was obtained after the -50 mV prepulse, and the TTXs component was obtained by digitally subtracting the TTXr I_{Na} from the total I_{Na}. EP recording was performed in differentiated hiPSC-SNs with extensive neurite growth at DIV21-28. To isolate somatic I_{Na}, a brief prepulse to voltage (-40mV) near spikes inactivating hiPSC-SN axonal spikes but not somatic spikes was performed, as described previously (26). Total, TTXs and TTXr I_{Na} in dissociated PSNs and differentiated hiPSC-SNs were also recorded with □□M of TTX in the bath solution.

High-voltage activated (HVA) I_{Ca} and Voltage-gated potassium channel current (I_{Kv}). HVA I_{Ca} was recorded in rat DRG dissociated neurons (sham-operated, AAV6-GFP, AAV6-NP, and AAV6-Na$_V$iPA1 transduced neurons, 4wk after AAV-DRG injection), and BK I_{Kv} recording were conducted in non-differentiated NG108-15 cells, as described previously (2).

Whole-cell current-clamp recording on dissociated DRG neurons (male rats). Whole-cell current-clamp recording of dissociated DRG neurons was performed, as described previously (11, 27, 28). Dissociated small- and medium-sized DRG neurons (≤40□m in diameter) from sham-operated animals, rats with TNI only, and dissociated DRG neurons with clear GFP expression from TNI rats injected with AAV6-GFPNP or AAV6-NaViPA1 at 8-week after TNI and 6-week after vector injection were used for recording (n=5 rats per group). The membrane input resistance was calculated by dividing the ending amplitude of steady-state hyperpolarizing voltage deflection

by the injected current (29). APs were generated by injection of a series of current pulses (180 to 280 pA in steps of 20 pA, 250 ms). The baseline potentials were recorded for 20 ms before the stimulus pulses were injected into the neurons. Resting membrane potential (RMP) was defined as the mean value of the 20 ms pre-stimulus potential in the first trial and the AP rheobase as the minimum current required to evoke the first AP. The neurons with stable resting membrane potentials (RMP) more negative than -40 mV and overshooting APs (>80 mV RMP to peak) were used for additional data collection. AP frequency was determined by quantifying the number of APs elicited in response to depolarizing current injections (250 ms).

Microinjection of AAV vectors into DRG

AAV vector solution was microinjected into right L4 and L5 DRG using previously described techniques (2, 30). Rats received L4 and L5 DRG injections of either AAV6-Na_ViPA1 or AAV6-NP (one vector per rat), consisting of 2 □L with adjusted titers containing a total of 2.0 x10^{10} genome viral particles. Sham in animals denotes that no injection after exposure surgery.

Animal pain model and behavior testing

TNI. Animals were anesthetized using isoflurane at 4% for induction and 2% for maintenance. TNI surgery was performed as we described previously (2). Sham-operated rats were subjected to all preceding procedures without nerve ligation and transection.

Stimulated behavior testing. Behavioral tests were conducted between 9:00 AM and 12:00 AM, as we described previously (2). The experimenters were blinded to the treatment during all data acquisition. Stimuli were applied to the lateral margin of the plantar aspect of the foot in the sural

area of innervation. Sensory tests included eliciting reflexive behaviors induced by von Frey test, Pin test, cold stimulation (acetone), and heat stimulation (Hargreaves test), and were carried out as previously described (30).

Gabapentin injection. Gabapentin (GBP, Sigma-Aldrich) was dissolved in saline immediately before injections and administered intraperitoneally (i.p.) at a volume of 0.5-1.0 ml (final dose=100 mg/kg body weight).

Conditioned place preference (CPP). A 3-chamber CPP apparatus was used (Med Associates, St. Albans, VT) in which 2 sliding doors separate the central chamber from the 2 side chambers that have distinct wall stripes and flooring. The CPP procedure: 1) On day 1, rats were acclimated to the CPP boxes for 30 min, with open access to each of the three chambers. On the preconditioning day, rats were placed in the grey middle chamber and allowed to explore both sides of chambers for 15 minutes and the time spent in each side was recorded, and the preferred and nonpreferred chambers were identified. 2) On the conditioning days, place conditioning was conducted using a biased assignment approach to drug pairing: saline was paired with the preferred chamber in the morning, and GBP was paired with the non-preferred chamber in the afternoon with a 6 hr interval (injections were never paired with the middle grey chamber). Conditioning consisted of the following sequential steps: intraperitoneal injection and restriction of the animal within the preferred chamber (saline) or non-preferred chamber (GBP) for 45 min. We used a 45 min conditioning time based on tests that gabapentin maximally reduced mechanical hypersensitivity at 30–60 min after i.p. injection (2). Animals were conditioned for 2 days since 2-day GBP has been reported sufficient to produce CPP in rodent pain models (31, 32); 3) For

postconditioning testing, the animals were placed back into the middle grey chamber of the CPP chambers with free access to all chambers for 15 minutes. The difference score for each animal was calculated, by subtracting the time spent in the saline-paired or GBP-paired chamber before pairing (during preconditioning) from the time spent in each chamber after pairing (postconditioning), and then averaged within each group. Each rat had only a single CPP test six weeks after AAV injection. A CPP is defined if the animals spend significantly more time in the GBP-paired chamber versus the saline-paired compartment.

Immunofluorescent staining

The previously described protocol was adopted (9). Primary antibodies: mouse GFP (1:500, Santa Cruz Biotechnology, SCB, CA. sc9996), rabbit GFP (1:500, Cell signaling, Danvers, MA. 2555), rabbit $Na_V1.7\alpha$ (1:400, Alomone, ASC008), rabbit $Na_V1.6\alpha$ (1:400, Alomone, ASC009), rabbit glial fibrillary acidic protein (GFAP, 1:1000, Dako, CA, Z0334), goat myelin basic protein (MBP, 1:500, SCB, sc13912), mouse neurofilament (NF200, 1:1000, Sigma-Aldrich, N6389), and mouse β3Tubulin (Tubb3, 1:500, SCB, sc-80016). The fluorophore-conjugated (Alexa 488 or Alexa 594, 1:2000) secondary antibodies (Jackson ImmunoResearch, West Grove, PA) were used to reveal immune complexes. The immunostaining was examined, and images were captured using a Nikon TE2000-S fluorescence microscope (El Segundo, CA) with filters suitable for selectively detecting the green and red fluorescence using a QuantiFire digital camera (Optronics, Ontario, NY). For measurement and quantification of immunostaining, positive marker antibody immunostainings were defined as the cells with the fluorescence intensity greater than average background fluorescence plus 2 standard deviations of the cells in an adjacent area in the same IHC slide of

negative control (the first antibody omitted) under identical acquisition parameters (n=10 for different markers). NIH ImageJ software was used for analysis.

Immunoblot

Immunoblots were performed as described previously (2). To examine the subcellular localization of $Na_V1.7$ in HEK1.7 cells and NaviPA1 and NaviPA1 mutants in HEK1.7 cells, the cell pellets were homogenized and then the plasma membrane, cytosolic, and nuclear fractions were extracted using the ProteoExtract Subcellular Proteome Extraction Kit (Millipore, Billerica, MA). In some experiments, the transferred PVDF membranes were cut into two halves along protein size around 70-100kDa and were subsequently incubated overnight at 4°C with appropriate antibodies. Antibodies: mouse GFP (1:1000), rabbit $Na_V1.7\alpha$ (1:1000), rabbit $Na_V1.8\alpha$ (1:1000, Alomone, ASC-016), rabbit Navβ2 (1:1000, Alomone, ASC-007), mouse Na^+/K^+ ATPase1α [illegible](NKA1α[illegible]SCB, sc514614), mouse Lamin B1 (1:1000, Proteintech, 66095), and mouse Gapdh (1:5000, Sigma-Aldrich, SAB1403850). Immunoreactive proteins were detected by Pierce enhanced chemiluminescence (ThermoFisher) on a ChemiDoc Imaging system (Bio-Rad) after incubation for 1 hr with HRP-conjugated second antibodies (1:5000, Bio-Rad).

GFPNa$_V$iPA1 affinity pull-down followed by immunoblots, silver staining, mass spectrometry, and PIP strip assay

GFP affinity pull-down. This was performed using ChromoTek GFP-Trap kit. Briefly, HEK1.7 cells were transiently transfected to express GFPNa$_V$iPA1 or GFPlinker and/or GFP1.7iPA2 as controls. After 48 h, cells were lysed with ice-cold nondenaturing lysis buffer containing (in mM): 10 Tris/Cl pH 7.5, 150 NaCl, 0.5 EDTA, with 0.5 % Nonidet P40 substitute (a non-ionic and non-

denaturing detergent), and protease inhibitor cocktail. The extracted cell lysates were diluted with 300 mL of dilution buffer containing (in mM) 10 Tris/Cl pH 7.5, 150 NaCl, 0.5 EDTA. For GFP-affinity pulldown, GFP-Trap Agarose Beads were equilibrated by adding 25 mL to 1.5 mL reaction tube with 500 mL of dilution buffer, cell lysates (2.5 mg total protein per sample) were incubated with GFP-Trap agarose by tube rotated end-over-end overnight at 4°C, followed by precipitation of beads by centrifugation at 12,000 rpm.

Silver stain, Nav1.7 immunoblot, and mass spectrometry. The extracted cell lysates (input) and GFP-affinity pulldown beads (pulldown) were performed for 4-20% SDS-PAGE gel, followed by immunoblots using $Na_V1.7$ antibody and silver stain on an additional SDS-PAGE gel using Pierce silver stain kit (ThermoFisher). The stained gel regions of interests were excised, and in-gel trypsin digested, as described previously (33). Extracted tryptic peptides were analyzed by nano reversed-phase liquid chromatography tandem mass spectrometry (nLC-MS/MS) using a nanoACQUITY (Waters Corporation, Milford, MA) online coupled with an Orbitrap Velos Pro hybrid ion trap mass spectrometer (ThermoFisher). The precursor ions were selected automatically by the instrument. Resultant MS/MS data were analyzed using the Mascot search engine (Matrix Science version 2.4) against the SWISSPROT human database.

PIP strip assay. NG108-15 cells transfected with $GFPNa_ViPA1$ and GFPlinker were prepared for cell lysate using a denaturing RIPA buffer containing (in mM): 10 Tris-HCl pH 7.5, 150 NaCl, 0.5 EDTA, with 0.1% SDS, 1% Triton X100, 1% deoxycholate, and protease inhibitor cocktail. The GFP-affinity pulldown beads from NG108-15 cells were size separated using 4-20% SDS-PAGE gels followed by silver stain. PIP strip assay (Echelon Biosciences, Salt Lake City, UT) was

performed to analyze interactions between purified GFPNa_ViPA1 from NG108-15 cells and membrane lipids, compared to purified GFP from NG108 as control. In brief, PIP (phosphatidylinositol phosphate) strip membranes were blocked in 3% (w/v) fatty acid-free BSA (Sigma-Aldrich) in TBST (50 mM Tris-HCl, pH 7.5, 150 mM NaCl, and 0.1% Tween 20) for 1 h. The membranes were then incubated in the same solution with the purified GFPNa_ViPA1 or GFP (equal 1.5 □g/mL) overnight at 4□C with gentle agitation. The membranes were washed 3 times over 30 min in fatty acid-free BSA-TBST. The membranes were incubated for 1h with 1:2000 dilution of HRP conjugated anti-GFP monoclonal antibody (Proteintech, HRP66002) at room temperature, then washed 6 times over 1 h in TBST, and the protein that was bound to the membrane by interaction with phospholipids was detected by enhanced chemiluminescence on a ChemiDoc Imaging system. PIP2 lipid spots: LPA, Lysophosphatidic acid; LPC, Lysophosphocholine; PtdIns, Phosphatidylinositol; PtdIns(3)P, Phosphatidylinositol (3) phosphate; PtdIns(4)P, Phosphatidylinositol (4) phosphate; PtdIns(5)P, Phosphatidylinositol (5) phosphate; PE Phosphatidylethanolamine; PC, Phosphatidylcholine; S1P, Sphingosine 1-Phosphate; PtdIns(3,4)P_2, Phosphatidylinositol (3,4) bisphosphate; PtdIns(3,5)P_2, Phosphatidylinositol (3,5) bisphosphate; PtdIns(4,5)P_2, Phosphatidylinositol (4,5) bisphosphate; PtdIns(3,4,5)P_3, Phosphatidylinositol (3,4,5) trisphosphate; PA, Phosphatidic acid; PS, Phosphatidylserine.

Supplementary data

SUPPLEMENTARY RESULTS

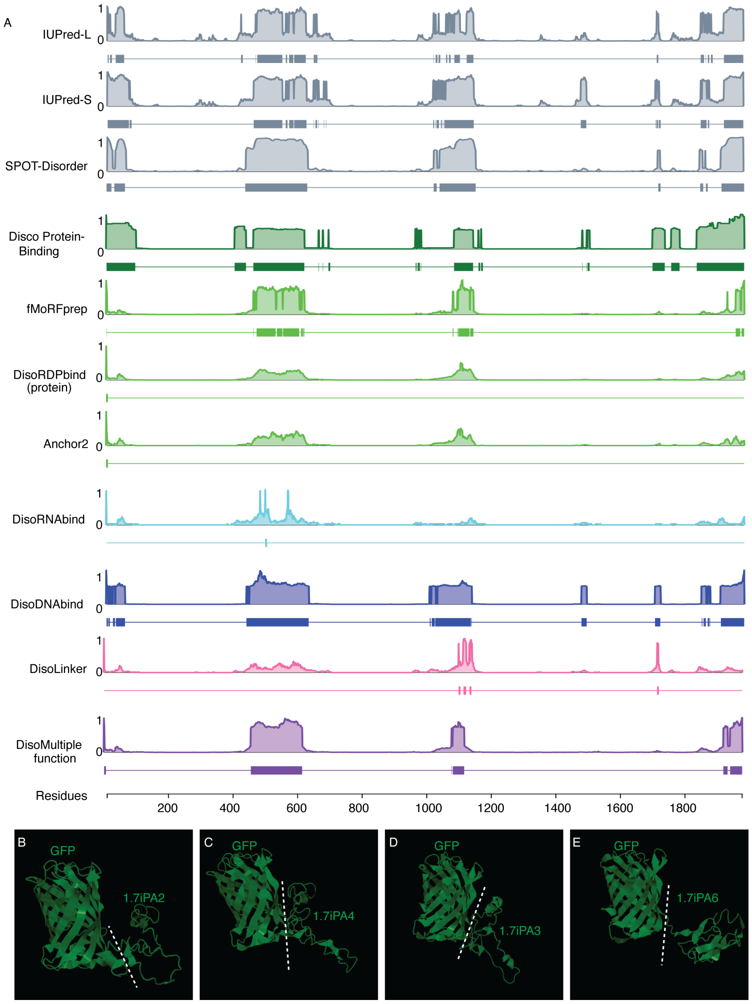

Supplemental Figure 1. In silico DEPICTER and I-TASSER. (**A**) IDRs are predicted by DEPICTER (4), a prediction algorithm that aggregates results from the multiple servers (IUPred-L and -S: Prediction of Intrinsically Unstructured Proteins. SPOT-Disorder-Single: Accurate Single-Sequence Prediction of Protein Intrinsic Disorder. Disco Protein Binding: Prediction of IDR Protein Binding. fMoRFpred: Fast Molecular Recognition Feature predictor. DisoRDPbind: Predictor of disorder-mediated RNA, DNA, and protein binding regions. Anchor 2: Potential binding sites in disordered regions. DiscoRNA(DNA) Bind: Prediction of Potential RNA (DNA) binding regions. Disordered: Flexible Linker predictor. DiscoMultipleFunction: Annotations of disordered multifunctional residues), with each algorithm indicated on the left side. (**B-E**) The crystal structure analysis of GFP1.7iPA2, 3, 4, and 6 by I-TASSER, as indicated.

Supplementary data

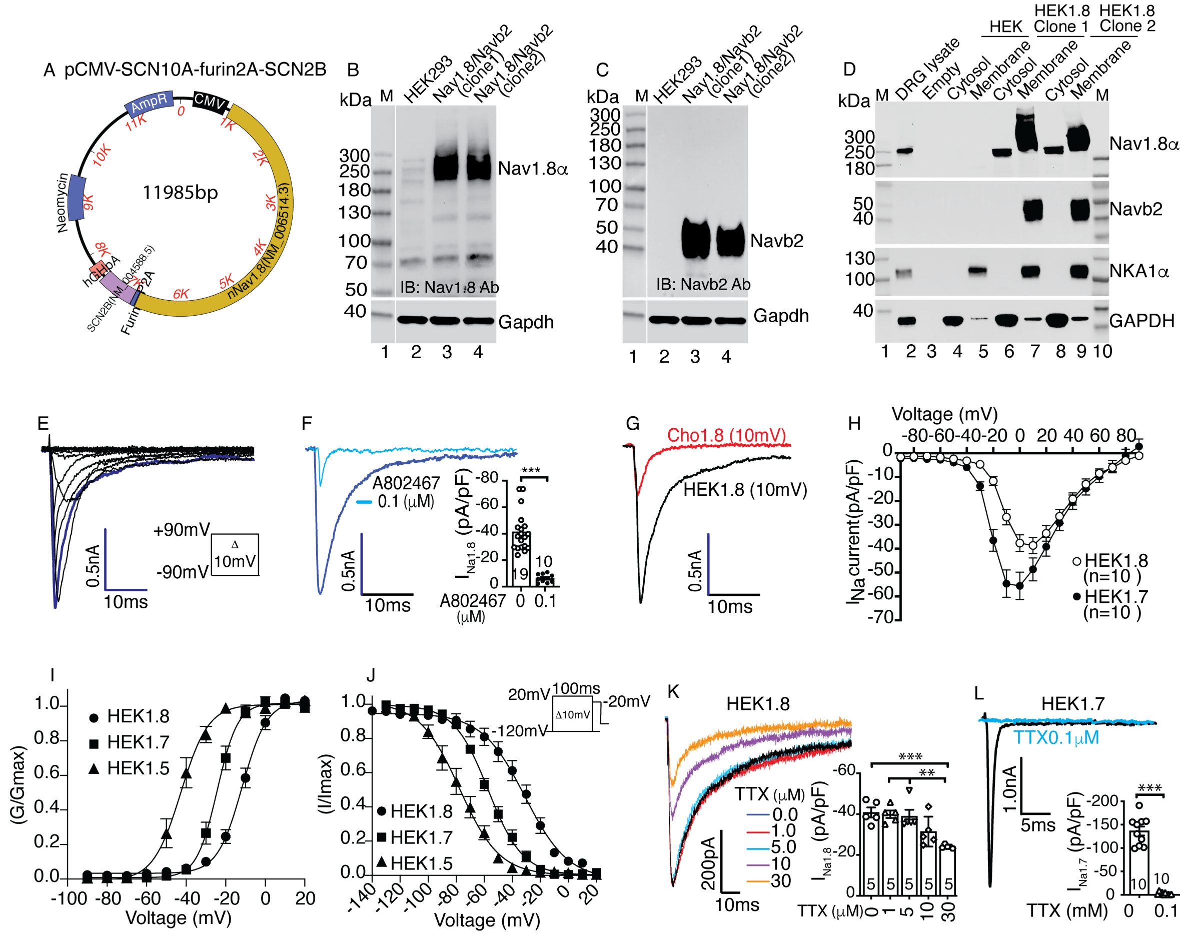

Supplemental Figure 2. Establishment of Nav1.8 stable expression system based on HEK cells. (A) An expression plasmid of pcDNA3.1(+)-SCN10A-Furin-P2A-SCN2B is constructed. (**B-C**) Immunoblots of cell lysates verify stable expression of Nav1.8α (**B**) and Navβ2 (**C**) (Vertical white lines in panels **B** and **C** denote that the lanes were run on the same gel but were noncontiguous), both are highly enriched in the plasma membrane (**D**). Traces of voltage-gated inward $I_{Na1.8}$ by whole-cell voltage-clamp recording (**E**), showing >85% inhibition by A802467 (0.1□M) (**F**) and comparison of single traces (+10mV) recorded from HEK1.8 cells and CHO1.8 cells (**G**). (**H**) Comparison of I/V curves between HEK1.7 and HEK1.8 cells. (**I** and **J**) Voltage-dependent activation and steady-state fast inactivation curves recorded from HEK1.8 cells, HEK1.7 cells, and HEK1.5 cells. Insert: recoding protocol. (**K**) Comparison of $I_{Na1.8}$ inhibition by TTX (1 to 30 □M□of HEK1.8 cells and (**L**) $I_{Na1.7}$ inhibition by TTX 0.□in HEK1.7 cells. ** and *** denote p<0.01 and 0.001, student t test (F and L), and one-way ANOVA and Tucky post hoc (K).

Supplementary data

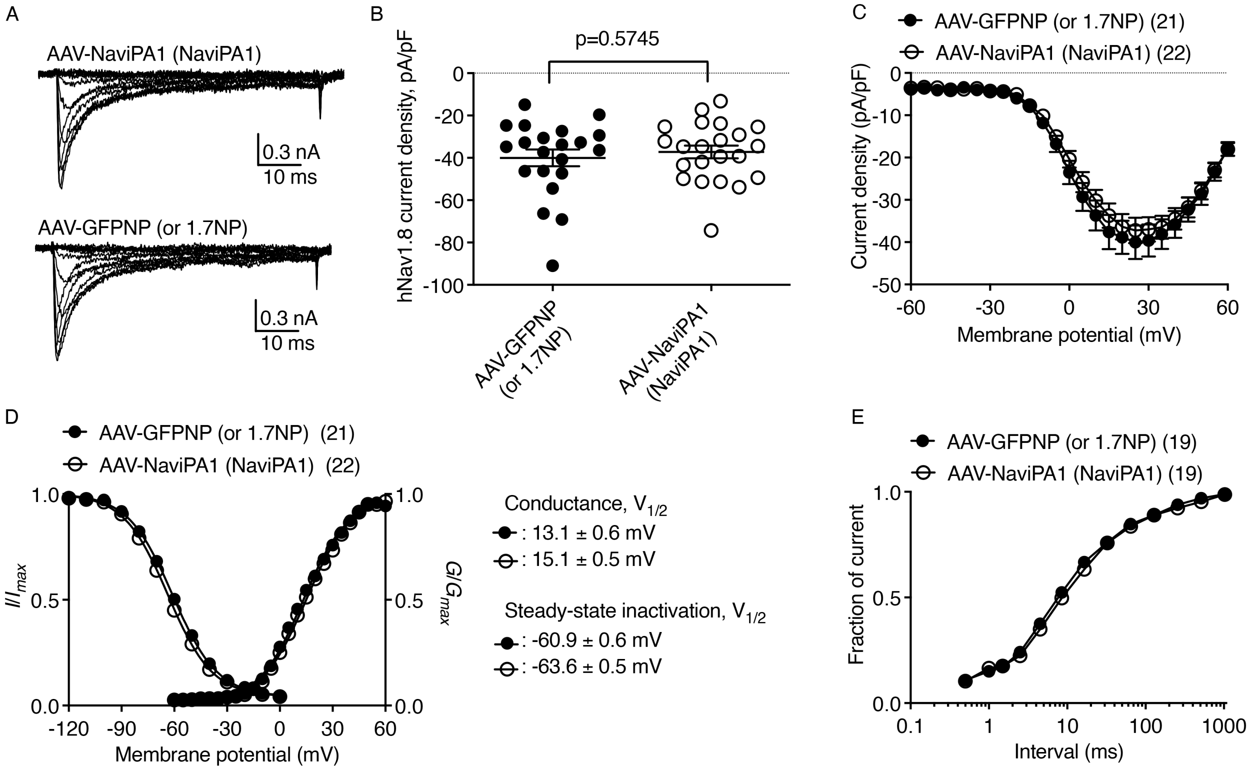

Supplemental Figure 3. Lack of Na$_V$iPA1 effect on $I_{Na1.8}$ transiently expressed in ND7/23 neuronal cells.

Representative $I_{Na1.8}$ currents recorded from Na$_V$1.8+ GFPNP and Na$_V$1.8 + Na$_V$iPA1 transfected ND7/23 cells (Sigma-Aldrich) (**A**). Scatter plot of peak current density of Na$_V$1.8+ GFPNP (n=21) and Na$_V$1.8 + Na$_V$iPA1 (n=22) transfected cells (**B**). I/V curves from Na$_V$1.8+ GFPNP and Na$_V$1.8 + Na$_V$iPA1 transfected cells (**C**). Comparison of the voltage-dependence of activation and inactivation for Na$_V$1.8+ GFPNP and Na$_V$1.8 + Na$_V$iPA1 transfected cells (**D**). Comparison of recovery from inactivation for Na$_V$1.8+ GFPNP and Nav1.8 + Na$_V$iPA1 transfected cells (**E**). For this set of experiments, the pipette solution contained (mM): CsCl 140, NaCl 10, EGTA 1.1, HEPES 10, pH7.30 and the bath solution contained (mM): NaCl 140, TEA-Cl 20, KCl 3, CaCl2 1, MgCl2 1, HEPES 10, pH7.30, with 1μM TTX.

Supplementary data

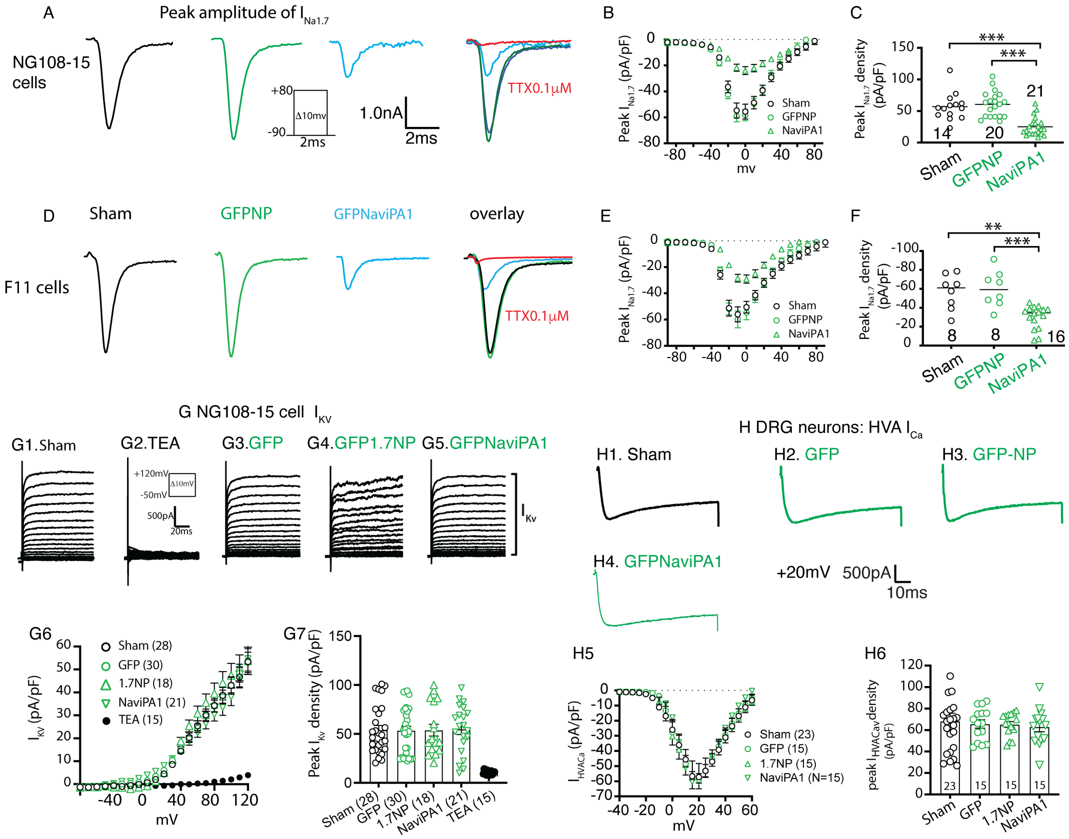

Supplemental Figure 4. NaviPA1 on $I_{Na1.7}$ of NG108 and F11 cells, I_{Kv} of NG108 cells, and HVA I_{Ca} of DRG neurons. Representative $I_{Na1.7}$ single traces at 0 mV recorded from sham-, GFPNP-, NaviPA1-transfected cells, merged $I_{Na1.7}$, I/V curves with curve of TTX (0.□□M), and peak $I_{Na1.7}$ density from NG108-15 cell (**A-C**) and F11 cells (**D-F**). Insets: protocol and current/time scales. **p<0.01 and ***p<0.001, one-way ANOVA and turkey post hoc. (**G**) Representative I_{Kv} of sham-NG108 cells showing I_{Kv} defined by outward currents blocked by Tetraethylammonium (TEA, 5mM) or NG108 cells transfected with GFP, 1.7NP, and NaviPA1 **(G1-G5)**. insets: recording protocol and current/time scales. I_{Kv} density-voltage (I/V) curves (**G6**) and quantitative analysis of peak I_{Kv} density (**G7**), p>0.05, one-way ANOVA and Tukey post hoc. (**H**) DRG neuron HVA I_{Ca} recording. Typical HVA I_{Ca} trace in a small-sized neuron from a naïve rat shows a threshold for activation around −30 mV and a maximum current amplitude activation at -10mV, displaying small inactivation (**H**). Typical traces of HVA I_{Ca} recorded at -10mV of neurons from a sham-operated rat (**H1**) and naïve rats injected with AAV6-GFP (**H2**), -1.7NP (**H3**), and -NaviPA1 (**H4**). HVA I_{Ca} density-voltage (I/V) curves (**H5**) and averaged peak HVA I_{Ca} density (**H6**), p>0.05, one-way ANOVA and Tukey post hoc.

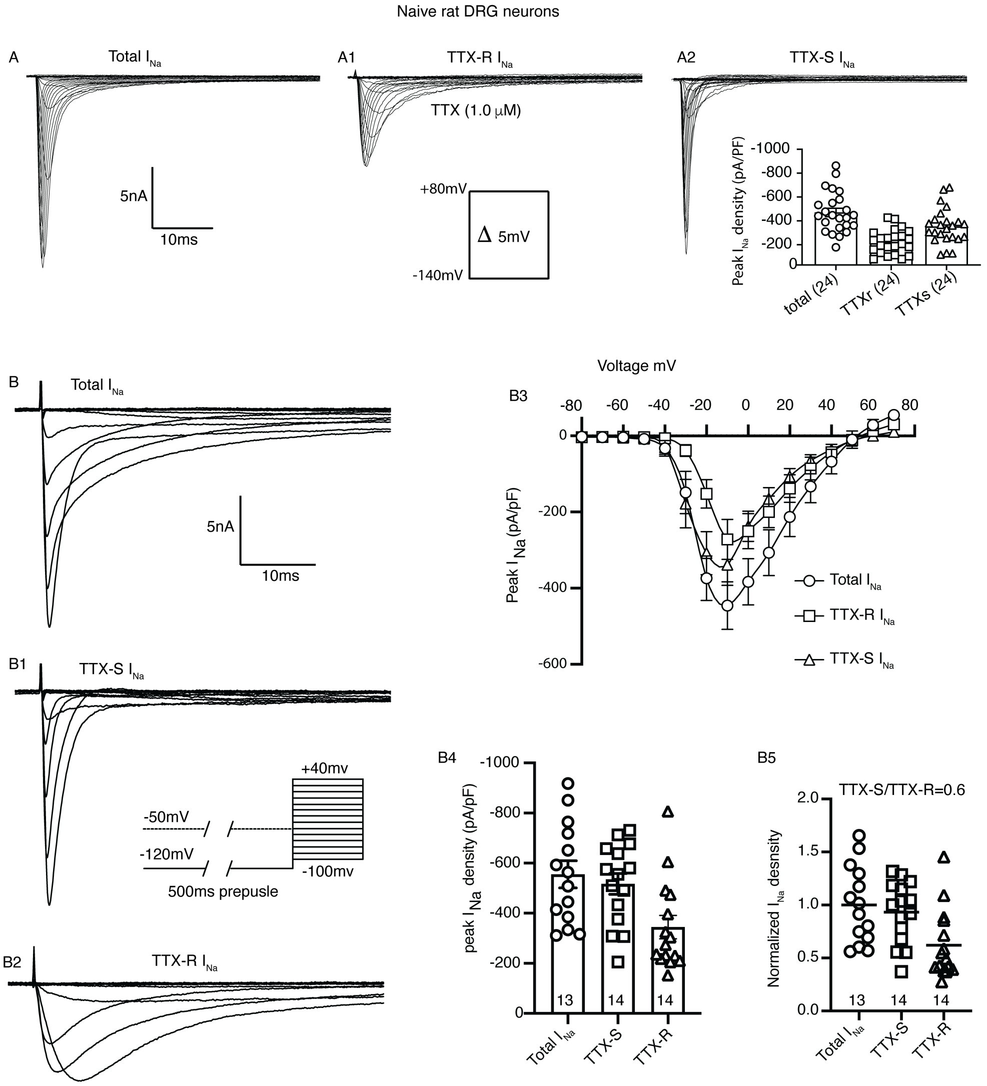

Supplemental Figure 5. **TTXs and TTXr I_{Na} recording in DRG neurons from naïve rats**. (**A-A2**) Representative traces of voltage-gated total, TTXr (bath TTX 1.0□M in the same cells), and TTXs (total I_{Na} subtracts TTXr I_{Na}) I_{Na} recorded from small-sized DRG neurons. Inserts: scales, voltage protocol, and quantification of peak I_{Na} densities. (**B-B2**) Representative traces of voltage-gated total, TTXs, and TTXr $I_{Na1.8\text{-like}}$ (manipulated by a voltage protocol) recorded from small-sized DRG neurons. Protocol for separation of TTXr and TTXs I_{Na}: A 500 ms prepulse to -120 or -50 mV was applied before a 50ms test pulse from-100 to +40mV with steps of 10 mV(inset). Both TTXs and TTXr I_{Na} were apparent after the -120 mV prepulse (top traces); only TTXr I_{Na} were obtained after the -50 mV prepulse (bottom traces), and the TTXs component was obtained (middle traces) by digitally subtracting the TTXr I_{Na} from the total I_{Na}. (**B3**) Average peak I_{Na} density–voltage relationships for total, TTXs, and TTXr I_{Na} of small DRG neurons. Smooth lines are I–V

curves generated using the Boltzmann fit parameters of the respective activation curves. Averaged peak I_{Na} densities (**B4**) and normalized peak I_{Na} densities (**B5**) of total, TTXs, and TTXr I_{Na}.

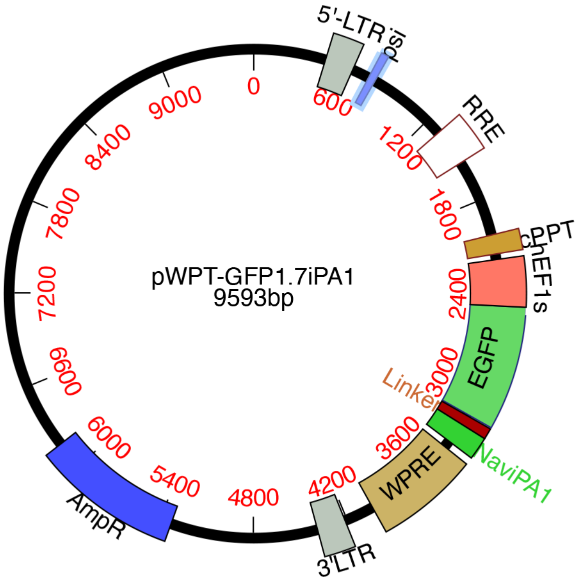

Supplemental Figure 6. Lentivector construct to express NaviPA1 and NP

Supplementary data

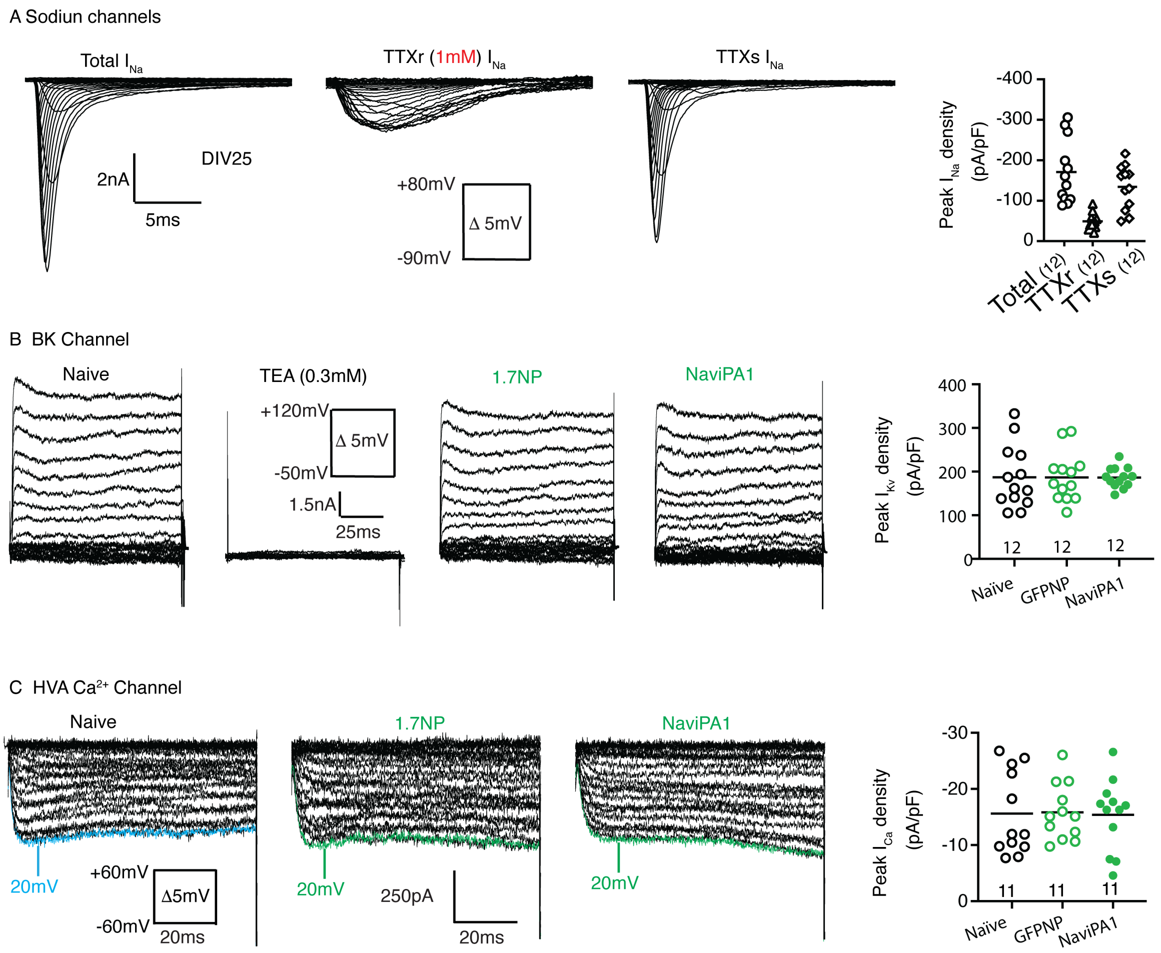

Supplemental Figure 7. I_{Na}, BK I_{Kv}, and HVA I_{Ca} recording from hiPSC-SNs. (**A**) Representative traces of total I_{Na}, TTXr I_{Na} (1□M TTX in bath solution), TTXs I_{Na}, recorded from naïve hiPSC-SNs (DIV25, Inserts: protocol and scales), and quantitative analysis of peak currents. A subtraction protocol is used to separate TTXr and TTXs INa. (**B**) Representative traces of BK I_{Kv} recorded from hiPSC-SNs (DIV21) in naïve, TEA in bath solution, Na$_V$iPA1- and NP-expressing hiPSC-SNs, and quantitative analysis of peak BK I_{Kv} density (panels from left to right). No difference, one-way ANOVA and post hoc. (**C**) Representative traces of HVA I_{Ca} recorded from hiPSC-SNs (DIV21) in naïve, Na$_V$iPA1- and NP-expressing hiPSC-SNs, and quantitative analysis of peak HVA I_{Ca} density (panels from left to right). No difference, one-way ANOVA and post hoc.

Supplementary data

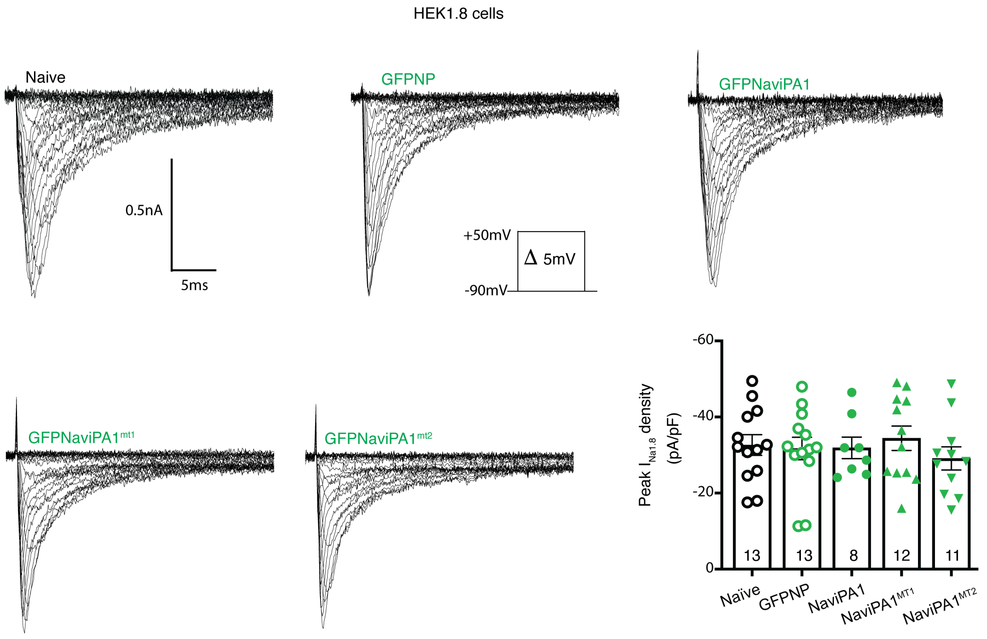

Supplemental Figure 8. **No effects of NaviPA1mt1 and 2 on I_{Na} in HEK1.8 cells**. Representative I_{Na} traces recorded from naïve and transfected HEK1.8 cells and peak current densities, as indicated.

Supplementary data

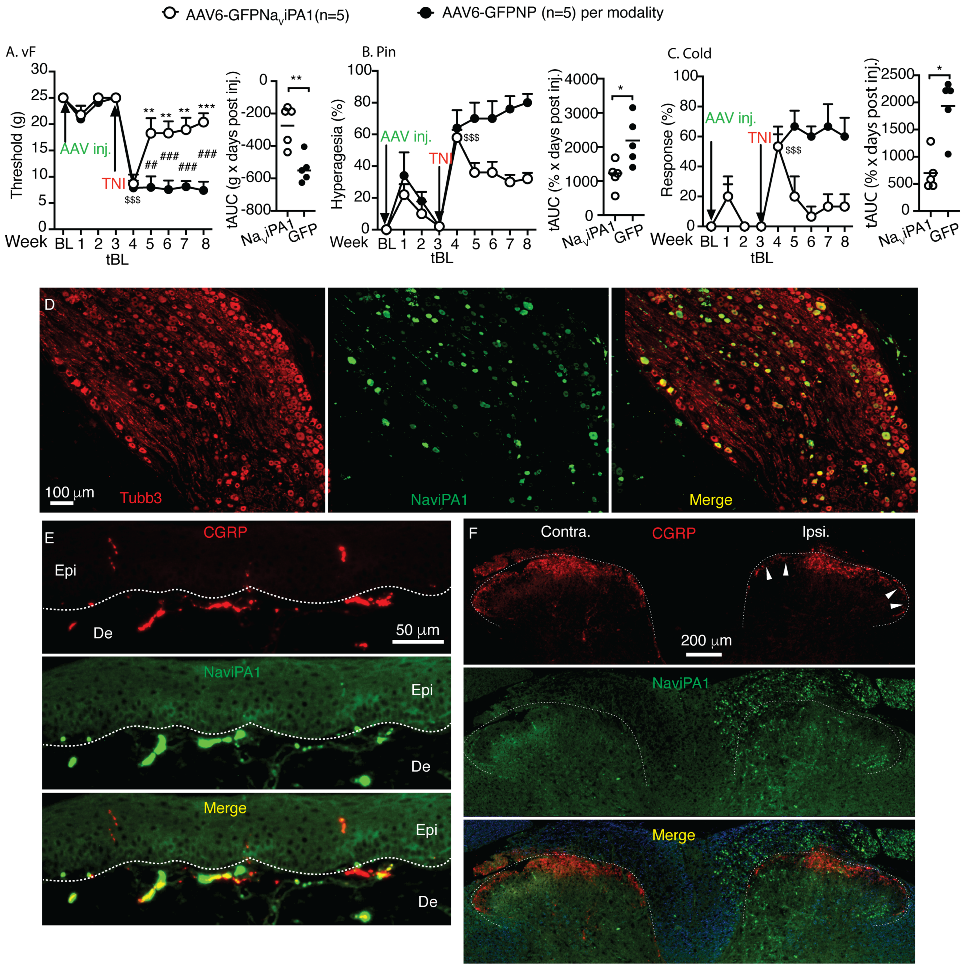

Supplemental Figure 9. In vivo pilot analgesic testing of NaviPA1. (**A-C**) Time courses for the group averages of sensitivity to vF, Pin, and Cold after DRG injection of either AAV6-NaviPA1 (n=5) or AAV6-NP (control, n=5) and subsequent TNI induction 3-wk after AAV injection. \$\$\$ denotes p<0.001, compared between tBL and 1 week after injection. **p<0.05 and ***p<0.001 (A) indicate comparisons to 1-week after AAV injection within groups, and $^{\#\#}$p<0.01 and $^{\#\#\#}$p<0.001 for comparisons between groups. Repeated measures parametric two-way ANOVA for vF and Heat followed by Tukey (within group) and Bonferroni (between groups) post hoc; and non-parametric Friedman ANOVA for Pin and Cold tests and Dunn's post hoc. Right panels of A-C show TNI tAUC calculated using measures 35-day post AAV; * p<0.05 and **p<0.01, unpaired, two-tailed Student's t-test for vF, and Mann–Whitney U tests for Pin and cold). Representative montage IHC images of DRG section (**D**, co-labeled GFP with Tubb3, showing colocalization in merged image), ipsilateral (ipsi.) hindpaw skin section **(E)** with dashed lines demarcating the dermis (De) and epidermis (Epi) boundaries, and spinal cord section (**F**) co-labeled GFP with CGRP (red), showing colocalization in merged image (arrowheads point to reduced CGRP innervation in ipsi. DH).

Supplementary data

References

1. Mogil JS. Sex differences in pain and pain inhibition: multiple explanations of a controversial phenomenon. *Nat Rev Neurosci.* 2012;13(12):859-66.
2. Shin SM, Lauzadis J, Itson-Zoske B, Cai Y, Fan F, Natarajan GK, et al. Targeting intrinsically disordered regions facilitates discovery of calcium channels 3.2 inhibitory peptides for adeno-associated virus-mediated peripheral analgesia. *Pain.* 2022;163(12):2466-84.
3. Madeira F, Park YM, Lee J, Buso N, Gur T, Madhusoodanan N, et al. The EMBL-EBI search and sequence analysis tools APIs in 2019. *Nucleic Acids Res.* 2019;47(W1):W636-W41.
4. Barik A, Katuwawala A, Hanson J, Paliwal K, Zhou Y, and Kurgan L. DEPICTER: Intrinsic Disorder and Disorder Function Prediction Server. *J Mol Biol.* 2020;432(11):3379-87.
5. Xue B, Dunbrack RL, Williams RW, Dunker AK, and Uversky VN. PONDR-FIT: a meta-predictor of intrinsically disordered amino acids. *Biochim Biophys Acta.* 2010;1804(4):996-1010.
6. Davey NE, Cowan JL, Shields DC, Gibson TJ, Coldwell MJ, and Edwards RJ. SLiMPrints: conservation-based discovery of functional motif fingerprints in intrinsically disordered protein regions. *Nucleic Acids Res.* 2012;40(21):10628-41.
7. Roy A, Kucukural A, and Zhang Y. I-TASSER: a unified platform for automated protein structure and function prediction. *Nat Protoc.* 2010;5(4):725-38.
8. Yu H, Fischer G, Ferhatovic L, Fan F, Light AR, Weihrauch D, et al. Intraganglionic AAV6 results in efficient and long-term gene transfer to peripheral sensory nervous system in adult rats. *PLoS One.* 2013;8(4):e61266.
9. Yu H, Fischer G, Jia G, Reiser J, Park F, and Hogan QH. Lentiviral gene transfer into the dorsal root ganglion of adult rats. *Mol Pain.* 2011;7:63.
10. Fang J, Yi S, Simmons A, Tu GH, Nguyen M, Harding TC, et al. An antibody delivery system for regulated expression of therapeutic levels of monoclonal antibodies in vivo. *Mol Ther.* 2007;15(6):1153-9.
11. Pan B, Guo Y, Wu HE, Park J, Trinh VN, Luo ZD, et al. Thrombospondin-4 divergently regulates voltage-gated Ca2+ channel subtypes in sensory neurons after nerve injury. *Pain.* 2016;157(9):2068-80.
12. Li R, Walsh P, Truong V, Petersen A, Dutton JR, and Hubel A. Differentiation of Human iPS Cells Into Sensory Neurons Exhibits Developmental Stage-Specific Cryopreservation Challenges. *Front Cell Dev Biol.* 2021;9:796960.
13. Fischer G, Pan B, Vilceanu D, Hogan QH, and Yu H. Sustained relief of neuropathic pain by AAV-targeted expression of CBD3 peptide in rat dorsal root ganglion. *Gene Ther.* 2014;21(1):44-51.
14. Herzog RI, Cummins TR, Ghassemi F, Dib-Hajj SD, and Waxman SG. Distinct repriming and closed-state inactivation kinetics of Nav1.6 and Nav1.7 sodium channels in mouse spinal sensory neurons. *J Physiol.* 2003;551(Pt 3):741-50.
15. Cummins TR, and Waxman SG. Downregulation of tetrodotoxin-resistant sodium currents and upregulation of a rapidly repriming tetrodotoxin-sensitive sodium current in small spinal sensory neurons after nerve injury. *J Neurosci.* 1997;17(10):3503-14.

16. Cummins TR, Dib-Hajj SD, and Waxman SG. Electrophysiological properties of mutant Nav1.7 sodium channels in a painful inherited neuropathy. *J Neurosci.* 2004;24(38):8232-6.
17. Theile JW, Jarecki BW, Piekarz AD, and Cummins TR. Nav1.7 mutations associated with paroxysmal extreme pain disorder, but not erythromelalgia, enhance Navbeta4 peptide-mediated resurgent sodium currents. *J Physiol.* 2011;589(Pt 3):597-608.
18. Dib-Hajj SD, Rush AM, Cummins TR, Hisama FM, Novella S, Tyrrell L, et al. Gain-of-function mutation in Nav1.7 in familial erythromelalgia induces bursting of sensory neurons. *Brain.* 2005;128(Pt 8):1847-54.
19. McDermott LA, Weir GA, Themistocleous AC, Segerdahl AR, Blesneac I, Baskozos G, et al. Defining the Functional Role of NaV1.7 in Human Nociception. *Neuron.* 2019;101(5):905-19 e8.
20. McCollum MM, Larmore M, Ishihara S, Ng LCT, Kimura LF, Guadarrama E, et al. Targeting the tamoxifen receptor within sodium channels to block osteoarthritic pain. *Cell Rep.* 2022;40(8):111248.
21. Tyagi S, Higerd-Rusli GP, Ghovanloo MR, Dib-Hajj F, Zhao P, Liu S, et al. Compartment-specific regulation of Na(V)1.7 in sensory neurons after acute exposure to TNF-alpha. *Cell Rep.* 2024;43(2):113685.
22. Minett MS, Nassar MA, Clark AK, Passmore G, Dickenson AH, Wang F, et al. Distinct Nav1.7-dependent pain sensations require different sets of sensory and sympathetic neurons. *Nat Commun.* 2012;3:791.
23. Huang J, Vanoye CG, Cutts A, Goldberg YP, Dib-Hajj SD, Cohen CJ, et al. Sodium channel NaV1.9 mutations associated with insensitivity to pain dampen neuronal excitability. *J Clin Invest.* 2017;127(7):2805-14.
24. Xiao Y, Theile JW, Zybura A, Pan Y, Lin Z, and Cummins TR. A-type FHFs mediate resurgent currents through TTX-resistant voltage-gated sodium channels. *Elife.* 2022;11.
25. Lopez-Santiago LF, Pertin M, Morisod X, Chen C, Hong S, Wiley J, et al. Sodium channel beta2 subunits regulate tetrodotoxin-sensitive sodium channels in small dorsal root ganglion neurons and modulate the response to pain. *J Neurosci.* 2006;26(30):7984-94.
26. Milescu LS, Bean BP, and Smith JC. Isolation of somatic Na+ currents by selective inactivation of axonal channels with a voltage prepulse. *J Neurosci.* 2010;30(22):7740-8.
27. Shin SM, Wang F, Qiu C, Itson-Zoske B, Hogan QH, and Yu H. Sigma-1 receptor activity in primary sensory neurons is a critical driver of neuropathic pain. *Gene Ther.* 2020.
28. Yu H, Pan B, Weyer A, Wu HE, Meng J, Fischer G, et al. CaMKII Controls Whether Touch Is Painful. *J Neurosci.* 2015;35(42):14086-102.
29. Joksimovic SL, Joksimovic SM, Tesic V, Garcia-Caballero A, Feseha S, Zamponi GW, et al. Selective inhibition of CaV3.2 channels reverses hyperexcitability of peripheral nociceptors and alleviates postsurgical pain. *Sci Signal.* 2018;11(545).
30. Fischer G, Kostic S, Nakai H, Park F, Sapunar D, Yu H, et al. Direct injection into the dorsal root ganglion: technical, behavioral, and histological observations. *J Neurosci Methods.* 2011;199(1):43-55.
31. Park HJ, Sandor K, McQueen J, Woller SA, Svensson CI, Corr M, et al. The effect of gabapentin and ketorolac on allodynia and conditioned place preference in antibody-induced inflammation. *Eur J Pain.* 2016;20(6):917-25.

32. Juarez-Salinas DL, Braz JM, Etlin A, Gee S, Sohal V, and Basbaum AI. GABAergic cell transplants in the anterior cingulate cortex reduce neuropathic pain aversiveness. *Brain.* 2019;142(9):2655-69.
33. Yu H, Wakim B, Li M, Halligan B, Tint GS, and Patel SB. Quantifying raft proteins in neonatal mouse brain by 'tube-gel' protein digestion label-free shotgun proteomics. *Proteome Sci.* 2007;5:17.
