# Supplementary material for: Peripherally targeted analgesia via AAV-mediated sensory neuron–specific inhibition of multiple pronociceptive sodium channels
Source: J Clin Invest. 2024 May 9;134(13):e170813. doi: 10.1172/JCI170813 (PMC11213509; doi:10.1172/JCI170813)
Supplement: Unedited blot and gel images [file jci-134-170813-s158.pdf]

Full unedited gels for **Fig. 1. Western blot**

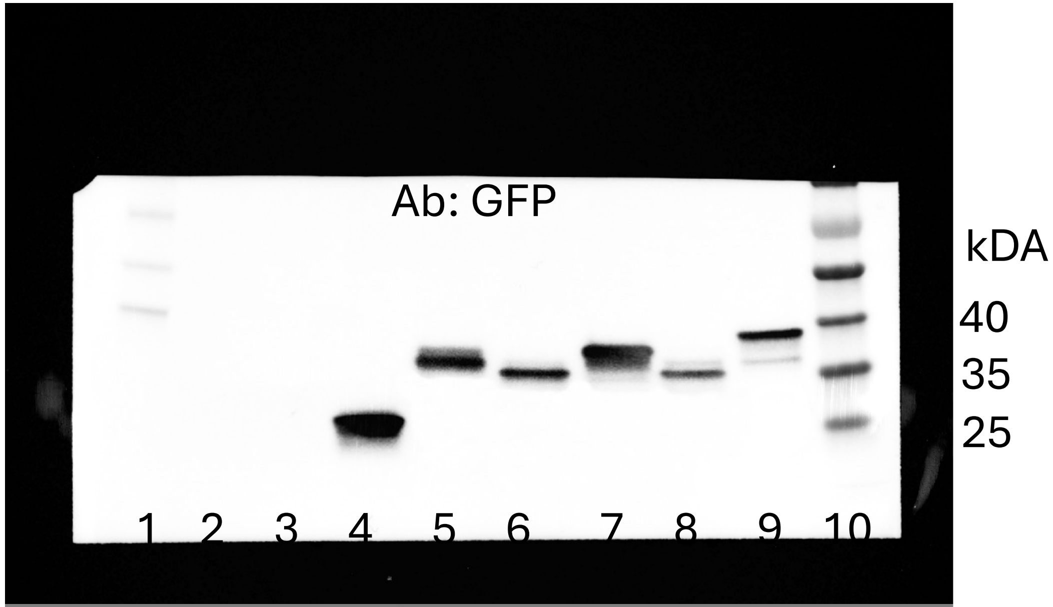

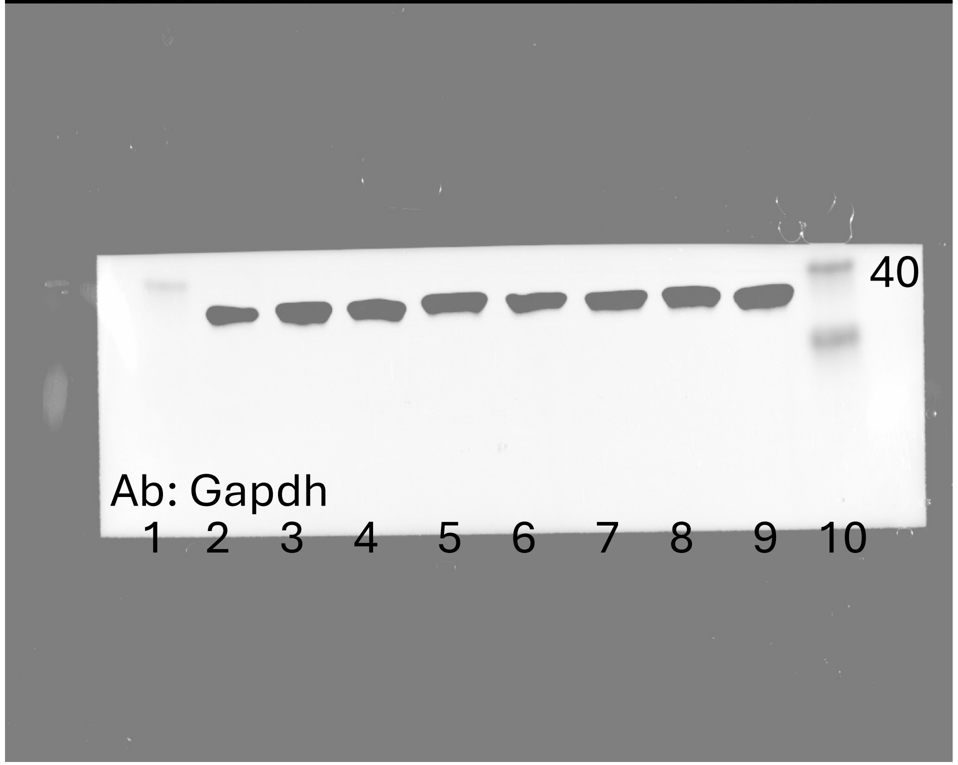

Antibody: Top, GFP; Bottom, Gapdh

Legend (HEK293 cell transfection)

Lane 1: protein ladder ((ThermoFisher 26616)
Lane 2, 3: Naive
Lane 4: GFP
Lane 5: GFPNav1.7iPA1
Lane 6: GFPNav1.7iPA3
Lane 7: GFPNav1.7iPA2
Lane 8: GFPNav1.7iPA4
Lane 9: GFPNav1.7iPA6
Lane 10: protein ladder (ThermoFisher: 26616)

Full unedited gels for **Fig. 5A. Western blot (cell lysates)**

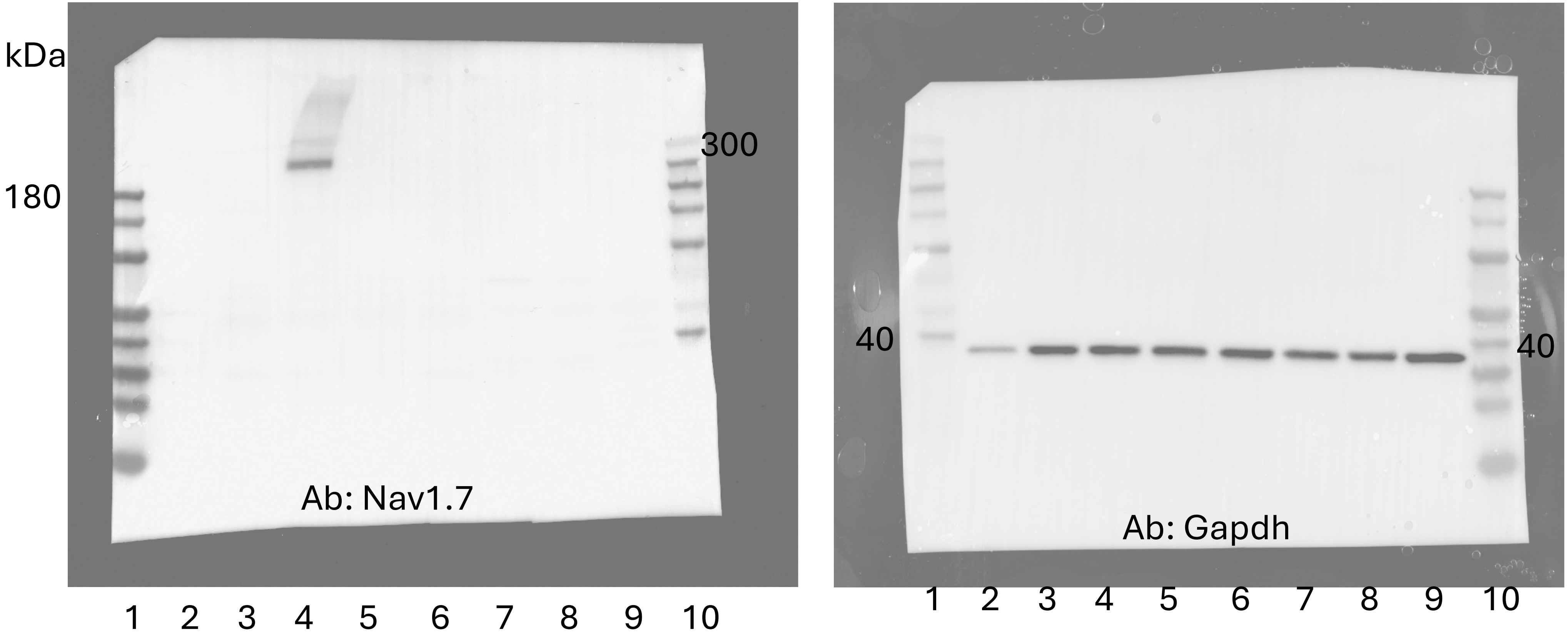

For both panels

Lane 1 and 10; protein ladders (thermoFisher 26625 and 26616)
Lane 2: Naïve HEK
Lane 3: HEK1.5 cells
Lane 4: HEK1.7 cells
Lane 5: HEK1.6 cells, Lane 6: HEK1.1 cells, Lane 7: HEK1.3 cells
Lane 6: HEK1.8 cells, Lane 9: 50B11 cells

Full unedited gels for **Figure 5F**. IBs of Na_V1.7, GFP, NKA1α, Gapdh (HEK1.7 cells transfected with GFP, NaviPA1 and 1.7iPA2)

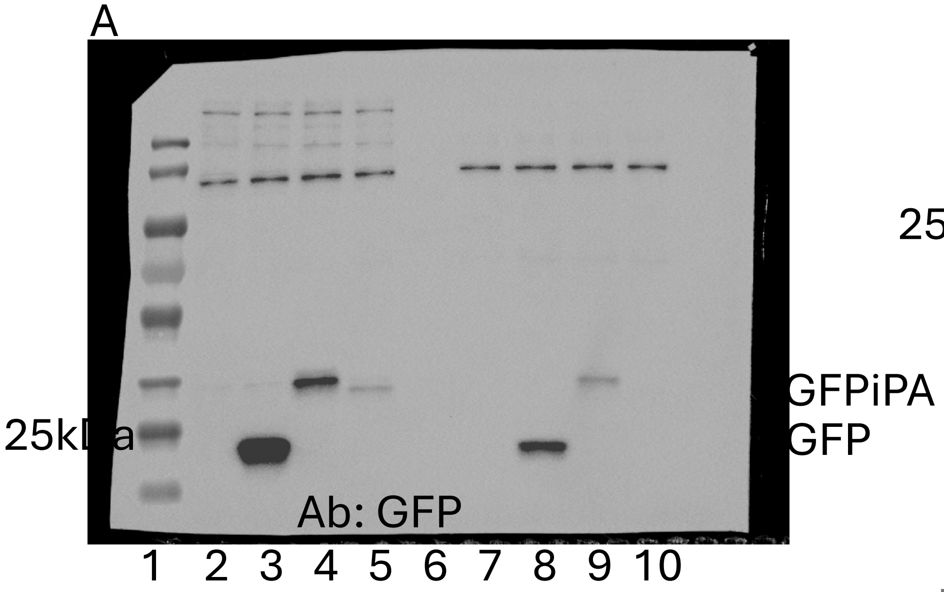

Panel A

Lane 1: Protein ladder, BioRad: 0373

Lane 2: Naïve cytosol

Lane 3: GFP, cytosol

Lane 4: NaviPA1, cytosol

Lane 5: 1.7iPA2, cytosol

Lane 6: empty

Lane 7: Naïve membrane

Lane 8: GFP, membrane

Lane 9: NaviPA1, membrane

Lane10: 1.7iPA2, membrane

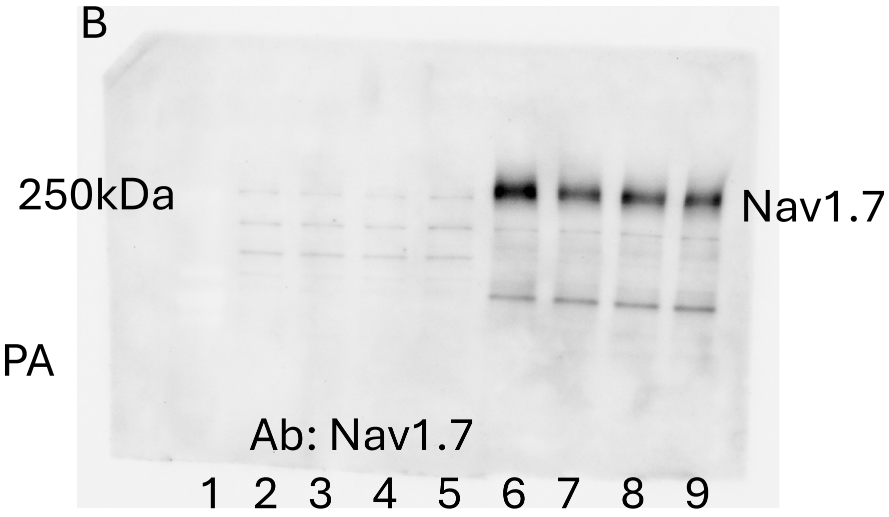

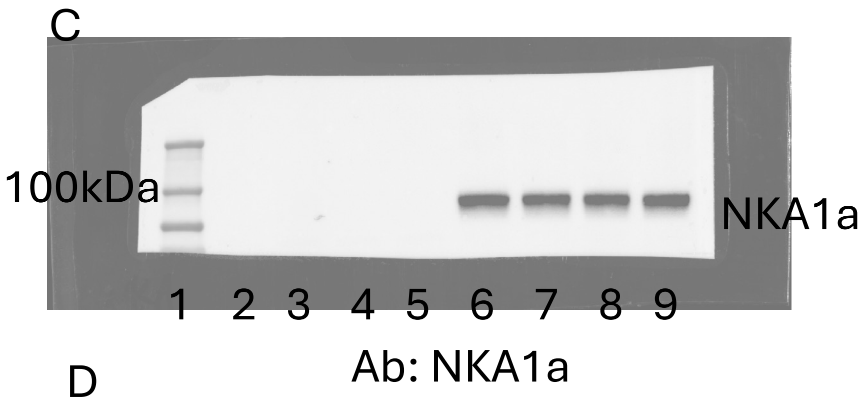

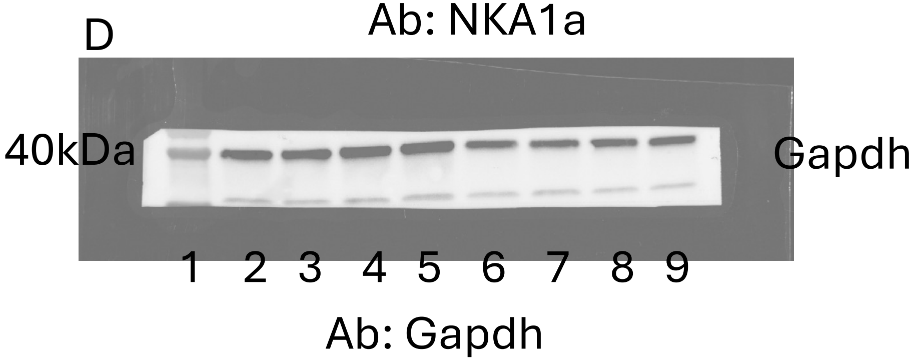

Panel B-C

Lane 1: Protein ladder, BioRad: 0373

Lane 2: Naïve cytosol

Lane 3: GFP, cytosol

Lane 4: NaviPA1, cytosol

Lane 5: 1.7iPA2, cytosol

Lane 6: Naïve membrane

Lane 7: GFP, membrane

Lane 8: NaviPA1, membrane

Lane 9: 1.7iPA2, membrane

Full unedited gel Fig. 5G repeat1. NaviPA1 GFP-affinity pull-down (HEK1.7 cells)

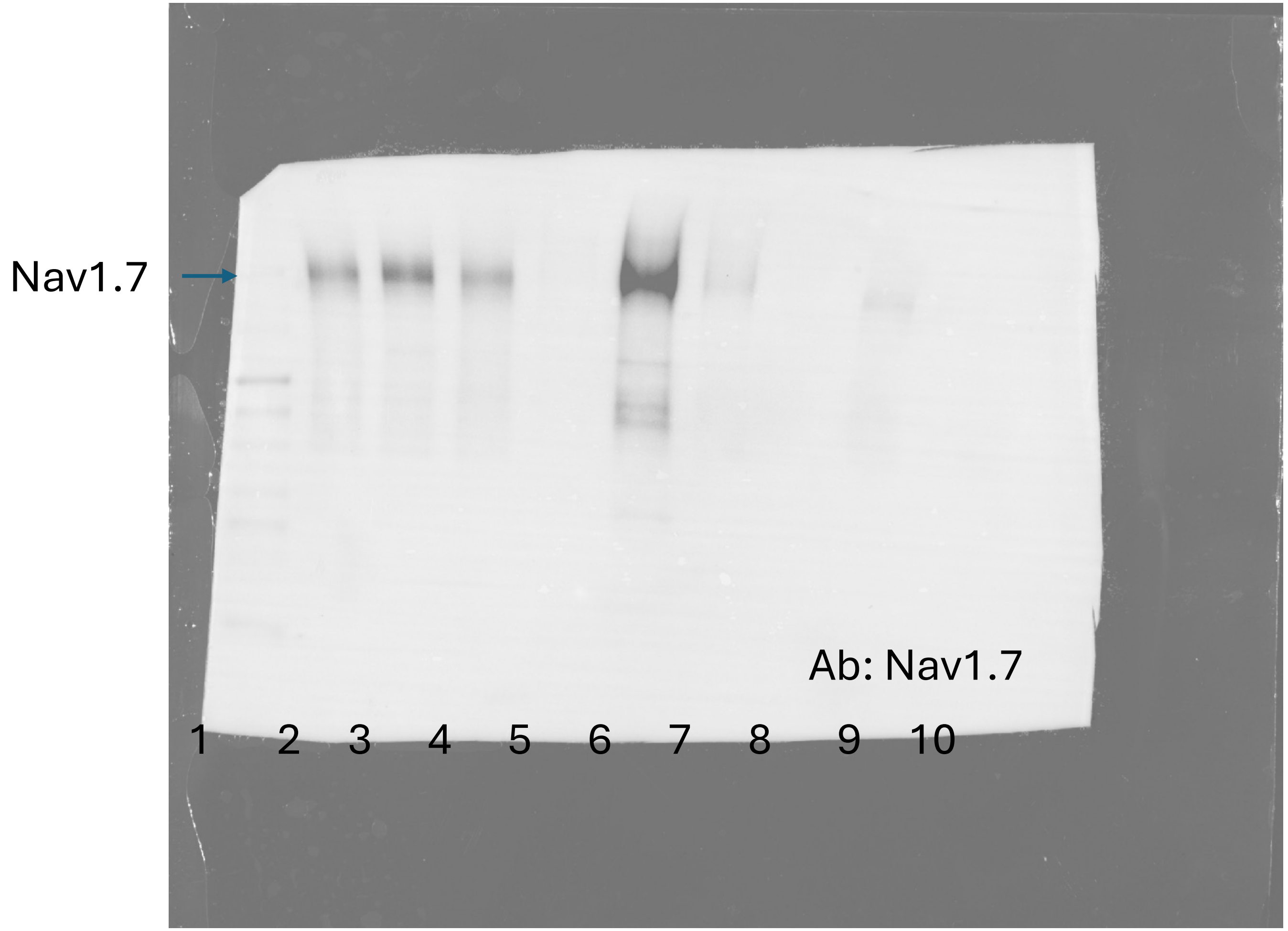

Lane 1: protein ladder Thermo 26625)
Lane 2: Input: GFP
Lane 3: Input: GFPNaviPA1
Lane 4: Input: GFPNav1.7iPA2
Lane 5: Pull-down beads: GFP
Lane 6: Pull-down beads: GFPNaviPA1
Lane 7: Pull-down beads: GFPNav1.7iPA2
Lane 8: Elution: GFP
Lane 9: Elution : GFPNaviPA1
Lane 10: Elution : GFPNav1.7iPA2

Full unedited gel Fig. 5G repeat2. NaviPA1 GFP-affinity pull-down (HEK1.7 cells)

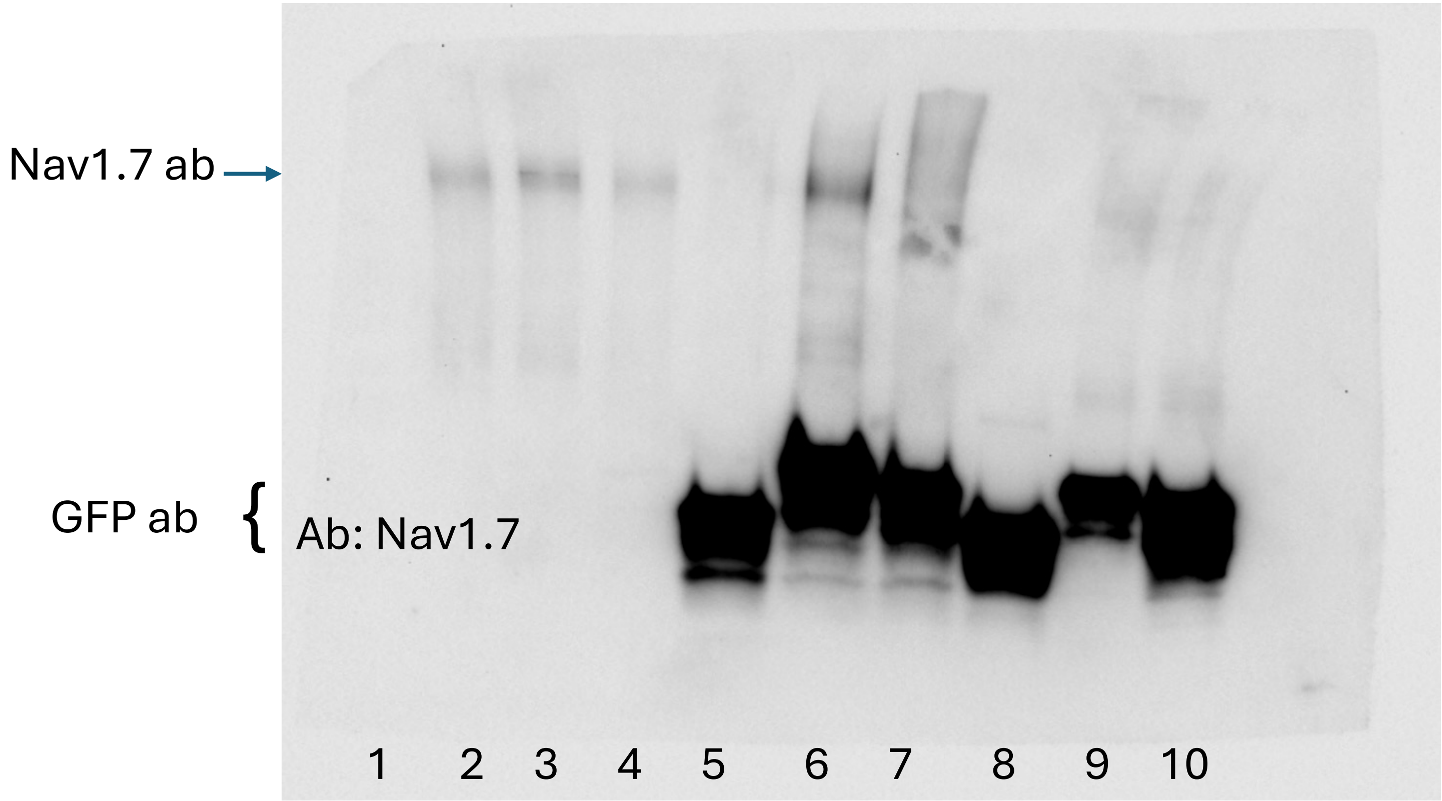

Lane 1: protein ladder Thermo 26625)
Lane 2: Input: GFP
Lane 3: Input: GFPNaviPA1
Lane 4: Input: GFPNav1.7iPA2
Lane 5: Pull-down beads: GFP
Lane 6: Pull-down beads: GFPNaviPA1
Lane 7: Pull-down beads: GFPNav1.7iPA2
Lane 8: Elution: GFP
Lane 9: Elution : GFPNaviPA1
Lane 10: Elution : GFPNav1.7iPA2

Full unedited gel Fig. 5G. Right panel: silver stain (HEK1.7 cells)

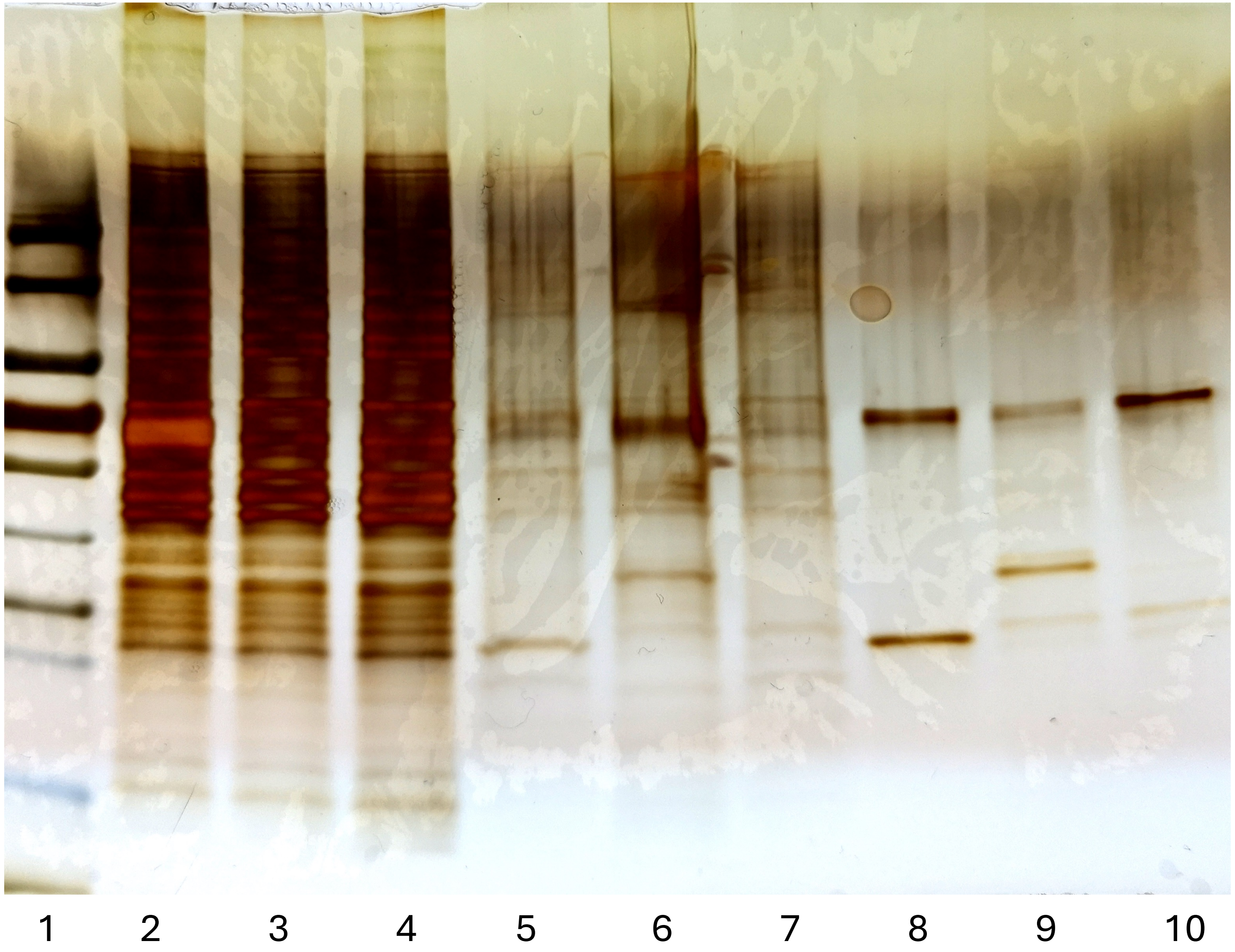

Lane 1: Protein ladder (Thermo 26625)
Lane 2: Input: GFPNP
Lane 3: Input: GFPNaviPA1
Lane 4: Input: GFPNav1.7iPA2
Lane 5: Affinity binding beads: GFPNP
Lane 6: Affinity binding beads: GFPNaviPA1
Lane 7: Affinity binding beads: GFPNav1.7iPA2
Lane 8: Elution: GFPNP
Lane 9: Elution: GFPNaviPA1
Lane 10: Elution: GFPNav1.7iPA2

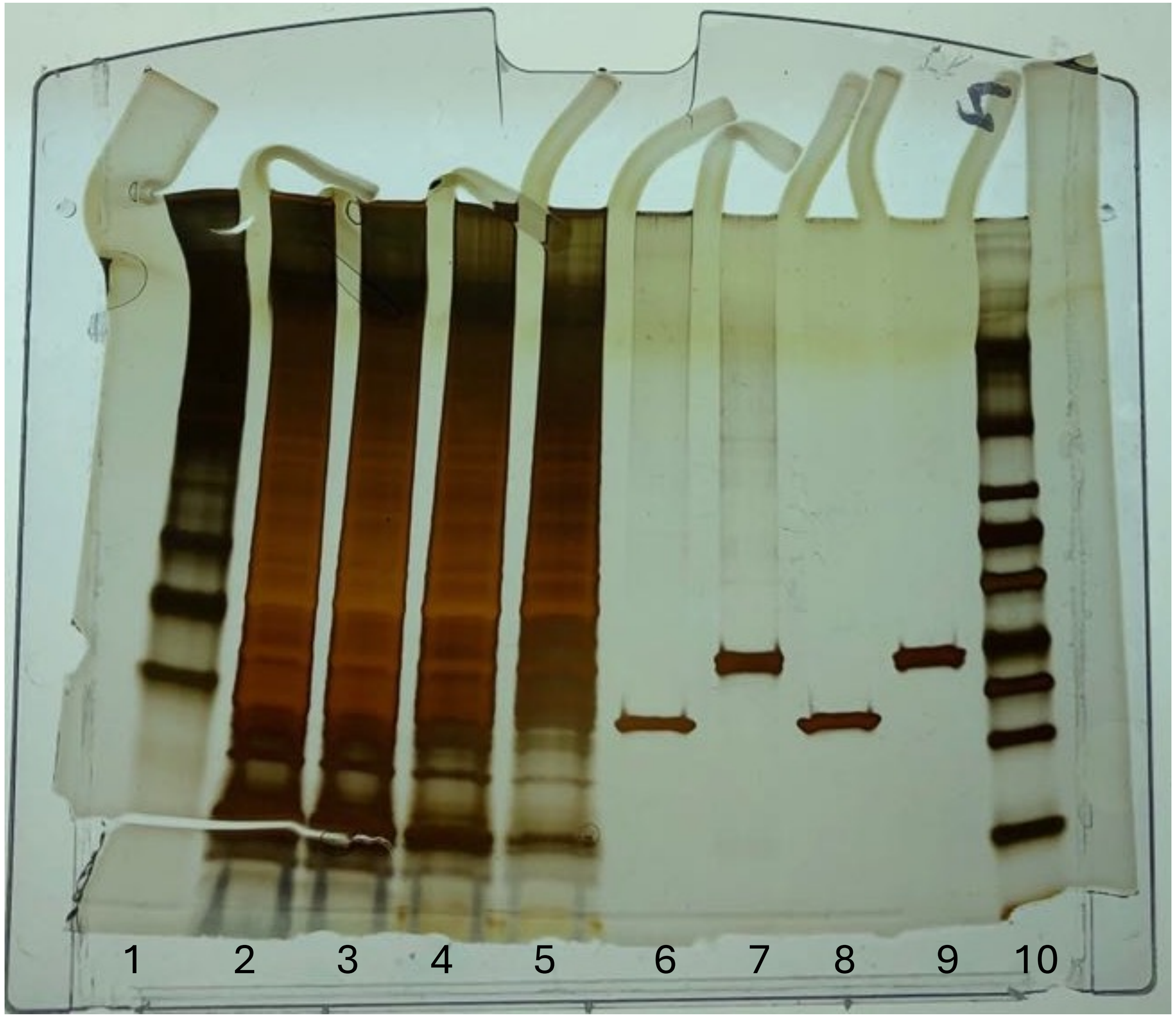

Full unedited gel **Fig. 5I**. Affinity pull-down silver stain (NG108 cell transfection)

Lane 1: Naive cell lysate control
Lane 2: Input: GFP
Lane 3: Input: GFPNaviPA1
Lane 4: Input: GFP
Lane 5: Input: GFPNaviPA1
Lane 6: Affinity pull-down: GFP
Lane 7: Affinity pull-down: GFPNaviPA1
Lane 8: Affinity pull-down: GFP
Lane 9: Affinity pull-down: GFPNaviPA1
Lane 10: Protein ladder (Thermo 26625)

Full unedited gels Fig. 5J repeat 1. PIP2 Strip (NG108 cells)

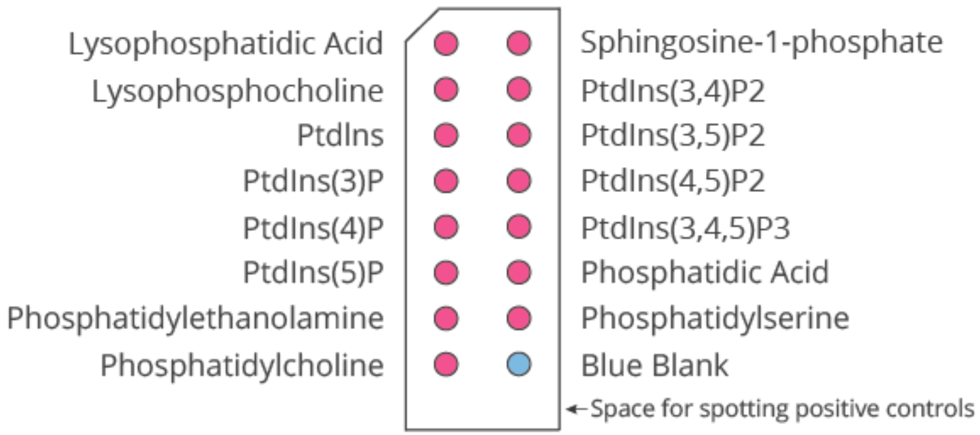

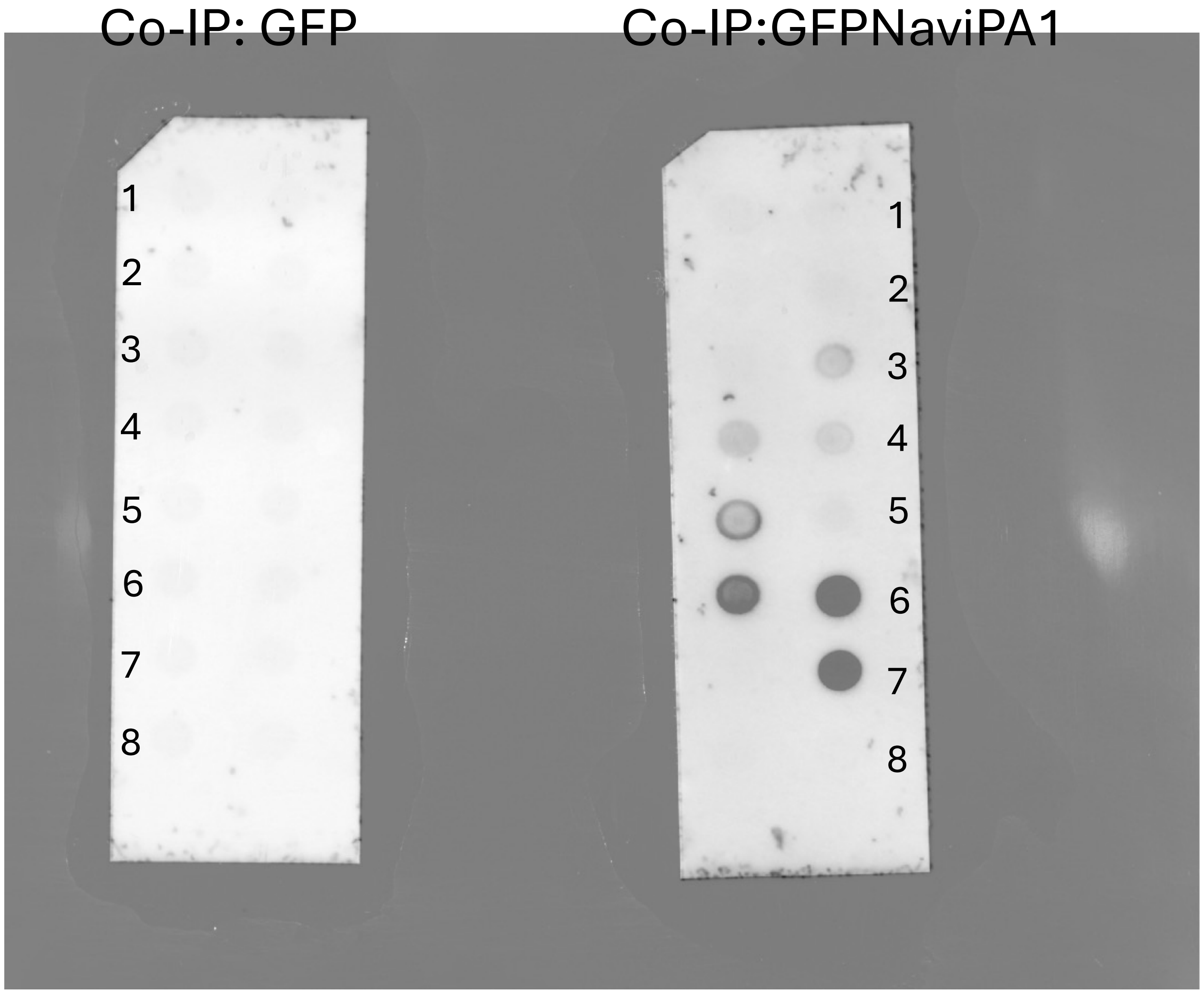

Full unedited gel Fig. 5J repeat 2. PIP2 Strip (NG108 cells)

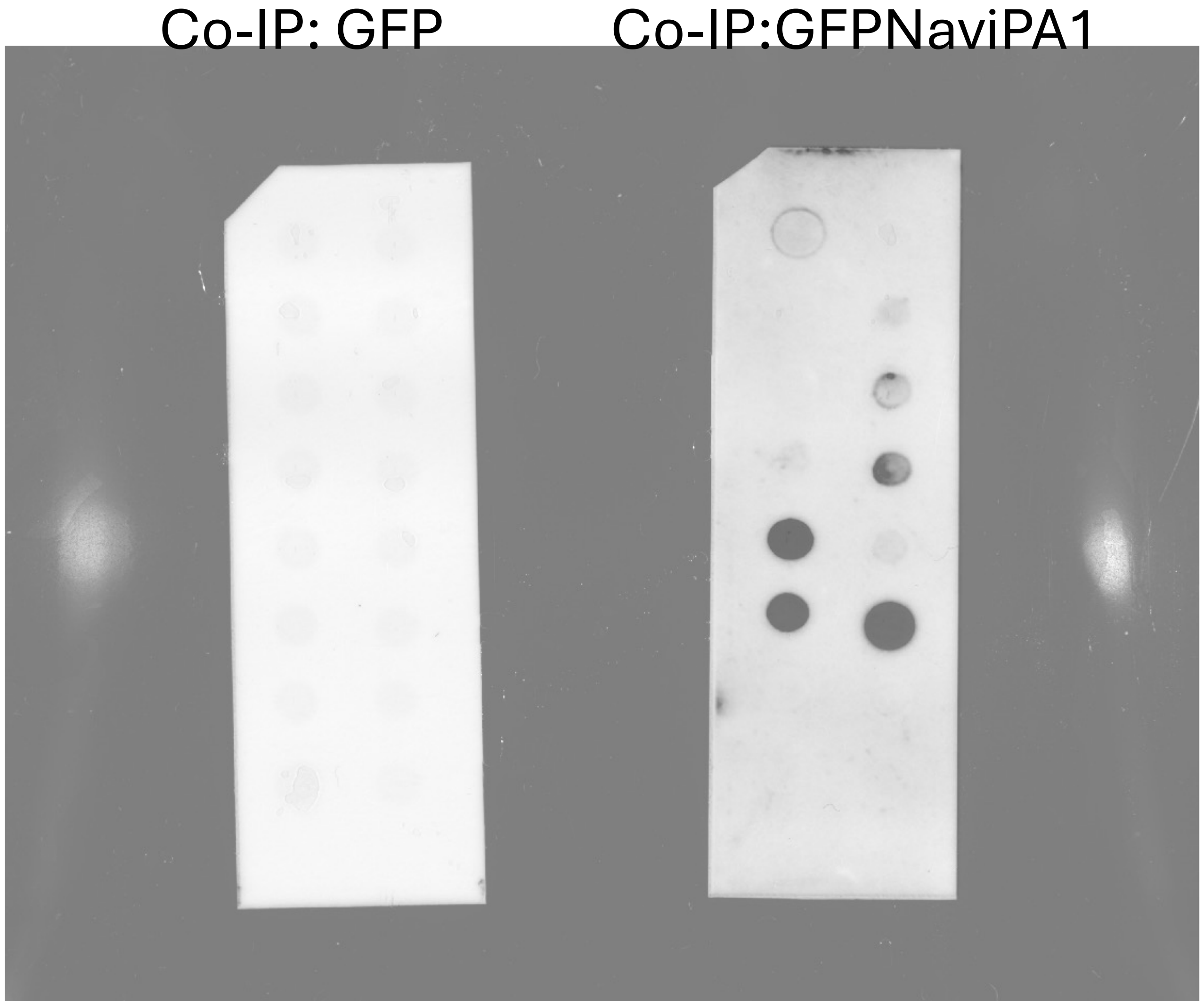

Full unedited gels for **Fig. 6F** repeat 1. HEK1.7 cells: GFPNP, NaviPA1, NaviPA1mt1, and NaviPA1mt2 cellular localization

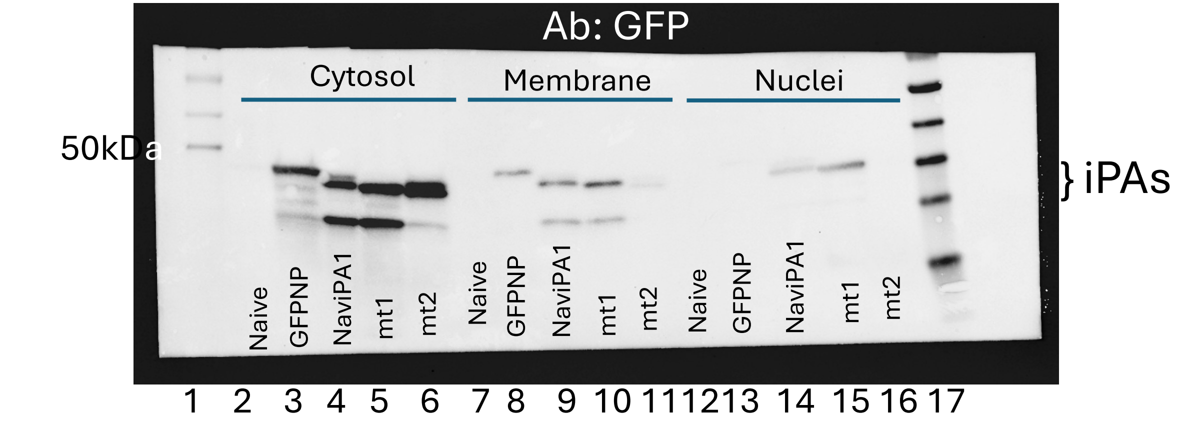

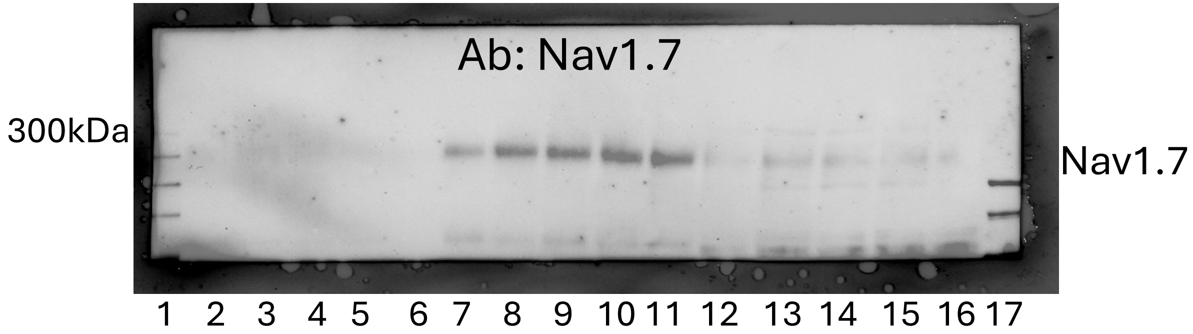

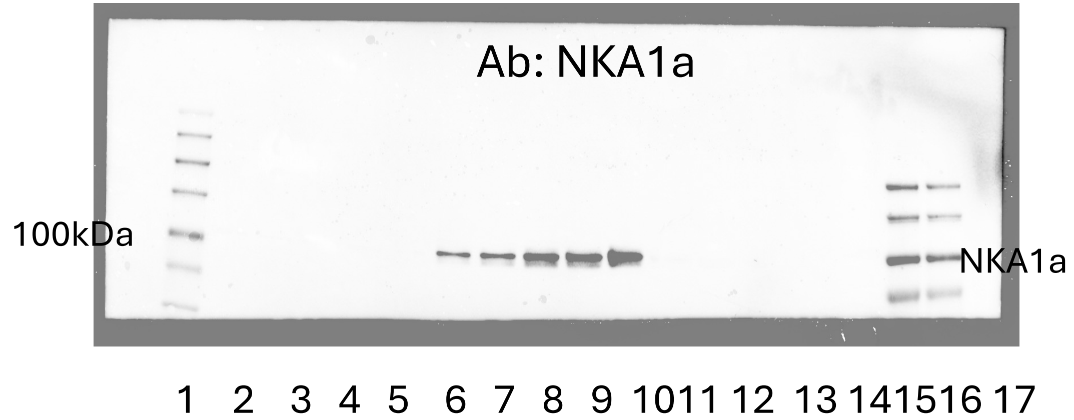

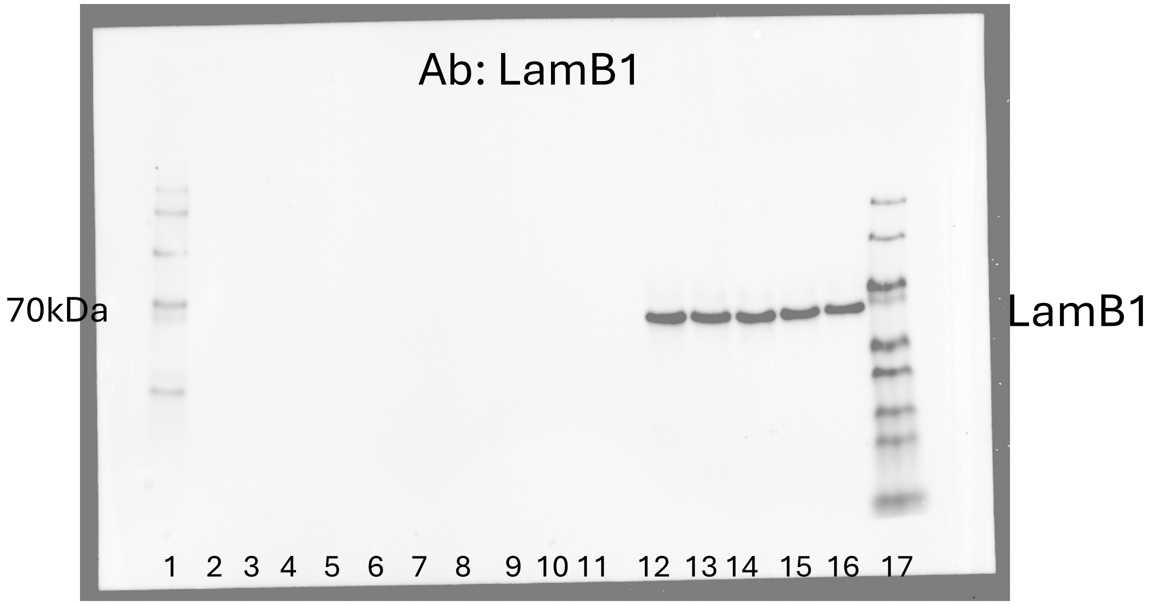

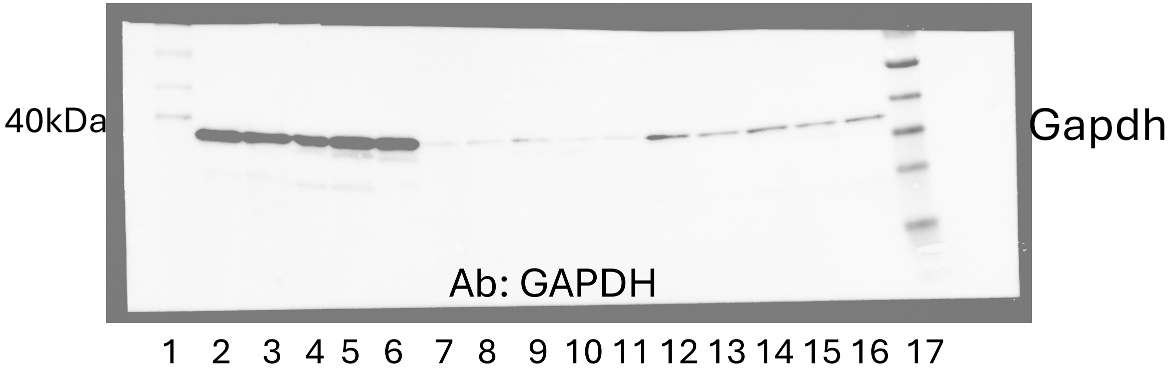

All panels: Lane 1: protein ladder (thermos 26625)and Lane 17: Protein ladder (thermos 26616)

Lanes 2-6: cytosol: Naïve, GFPNP, NaviPA1, NaviPA1mt1, and NaviPA1mt2

Lanes 7-11: Membrane: Naïve, GFPNP, NaviPA1, NaviPA1mt1, and NaviPA1mt2

Lanes 12-16: Nuclei: Naïve, GFPNP, NaviPA1, NaviPA1mt1, and NaviPA1mt2

Full unedited gels for **Fig. 6F** repeat 2. HEK1.7 cells: GFPNP, NaviPA1, NaviPA1mt1, and NaviPA1mt2 cellular localization

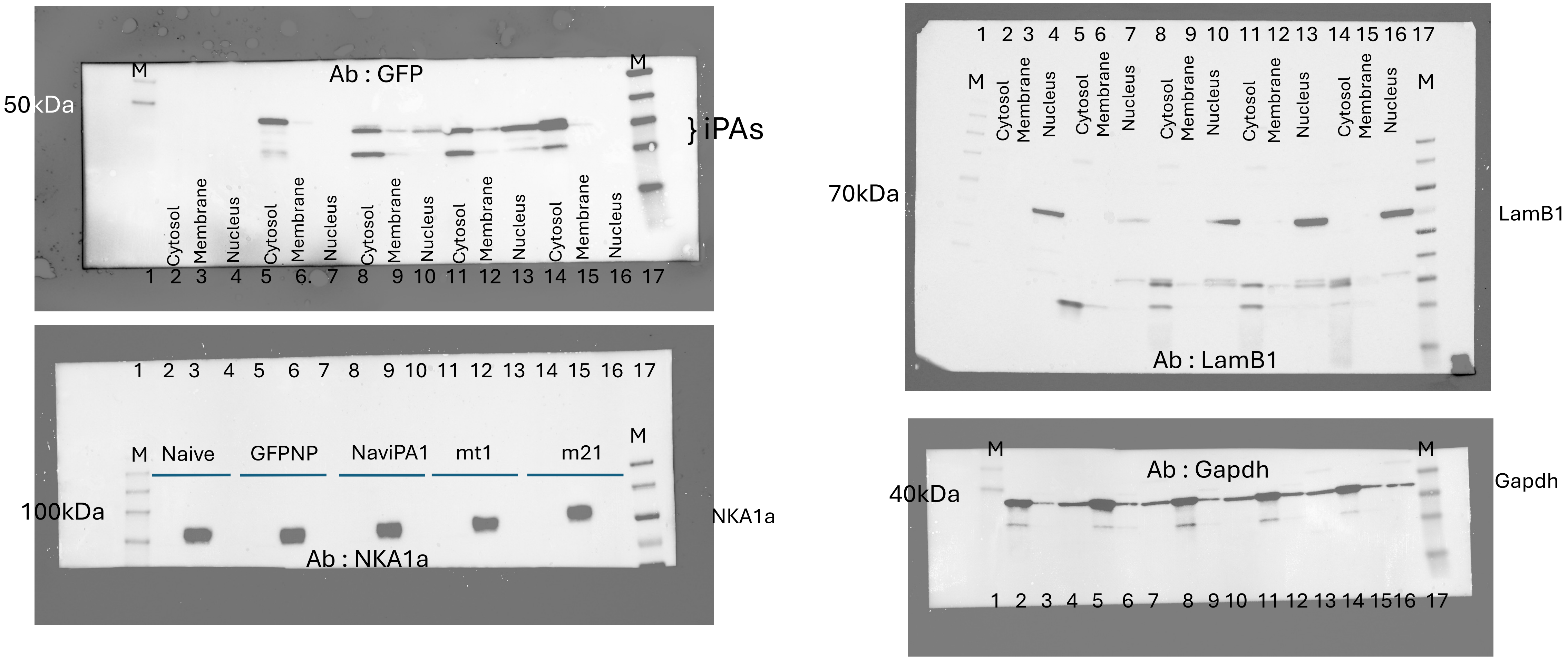

All panels: Lane 1: protein ladder (thermos 26625)and Lane 17: Protein ladder (thermos 26616)
Lanes 2-4: Naïve: cytosol, membrane, and nuclei
Lanes 5-7: GFPNP: cytosol, membrane, and nuclei
Lanes 8-10: NaviPA1: cytosol, membrane, and nuclei
Lanes 1-13: mt1: cytosol, membrane, and nuclei
Lanes 1-13: mt2: cytosol, membrane, and nuclei

Full unedited gels for **Fig. 6F** repeat 3. HEK1.7 cells: GFPNP, NaviPA1, NaviPA1mt1, and NaviPA1mt2 cellular localization

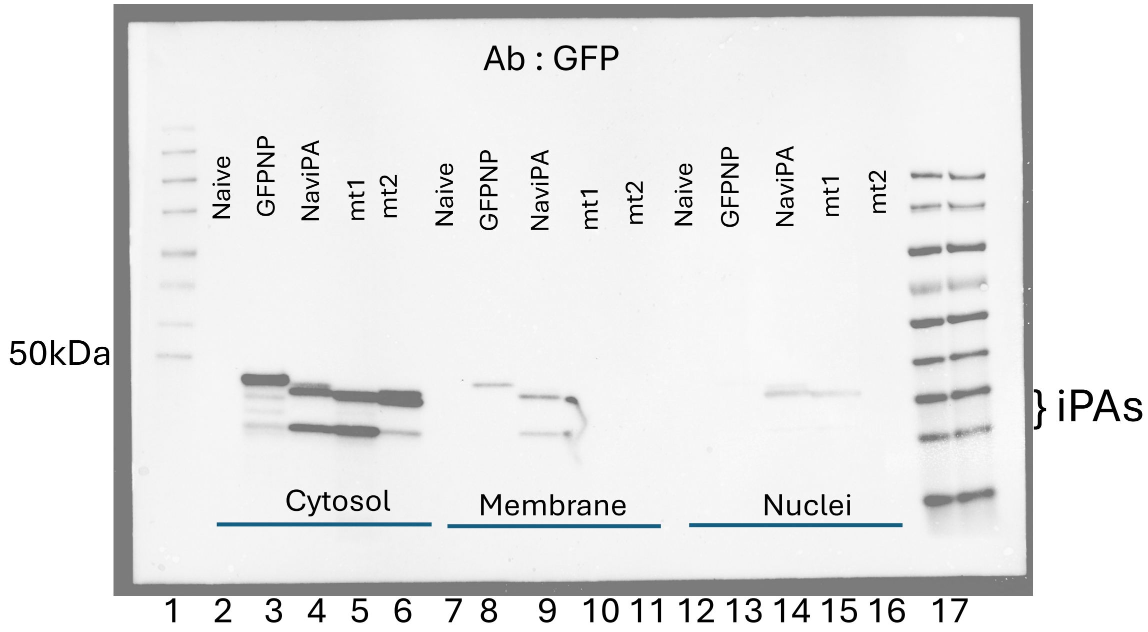

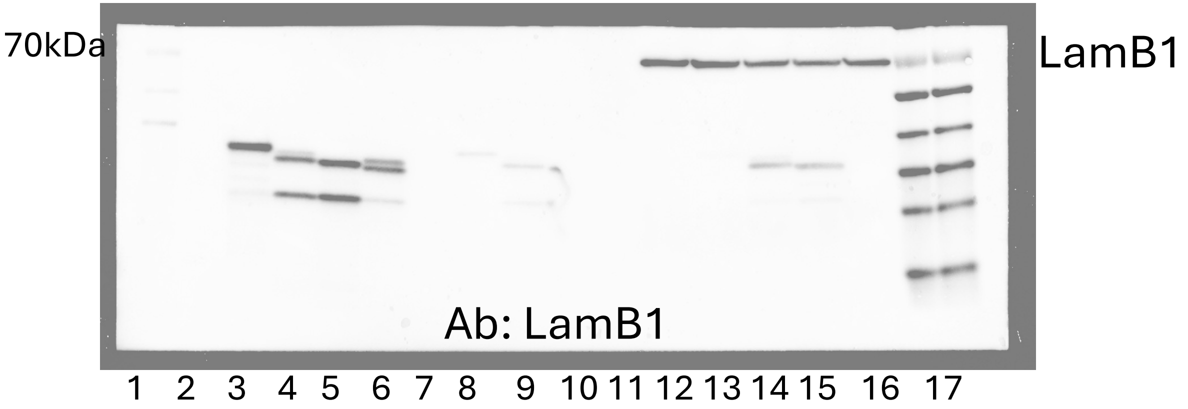

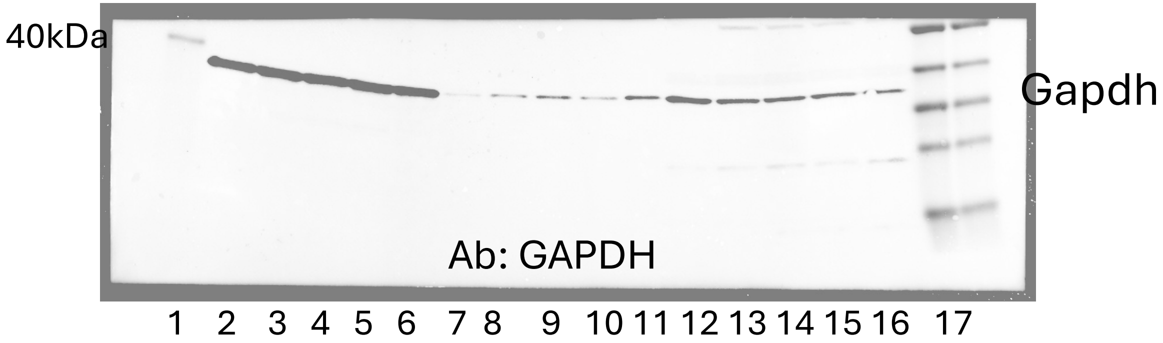

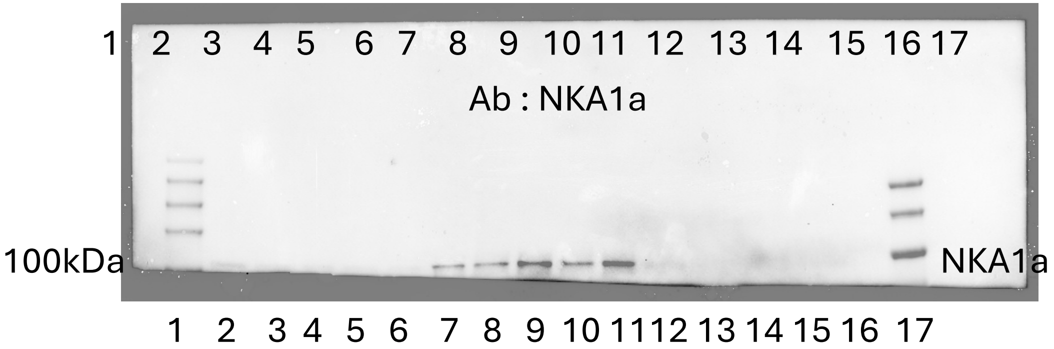

All panels: Lane 1: protein ladder (thermos 26625)and Lane 17: Protein ladder (thermos 26616)

Lanes 2-6: cytosol: Naïve, GFPNP, NaviPA1, NaviPA1mt1, and NaviPA1mt2

Lanes 7-11: Membrane: Naïve, GFPNP, NaviPA1, NaviPA1mt1, and NaviPA1mt2

Lanes 12-16: Nuclei: Naïve, GFPNP, NaviPA1, NaviPA1mt1, and NaviPA1mt2

Full unedited gel for **Fig. 7A**: Purified AAV silver stain

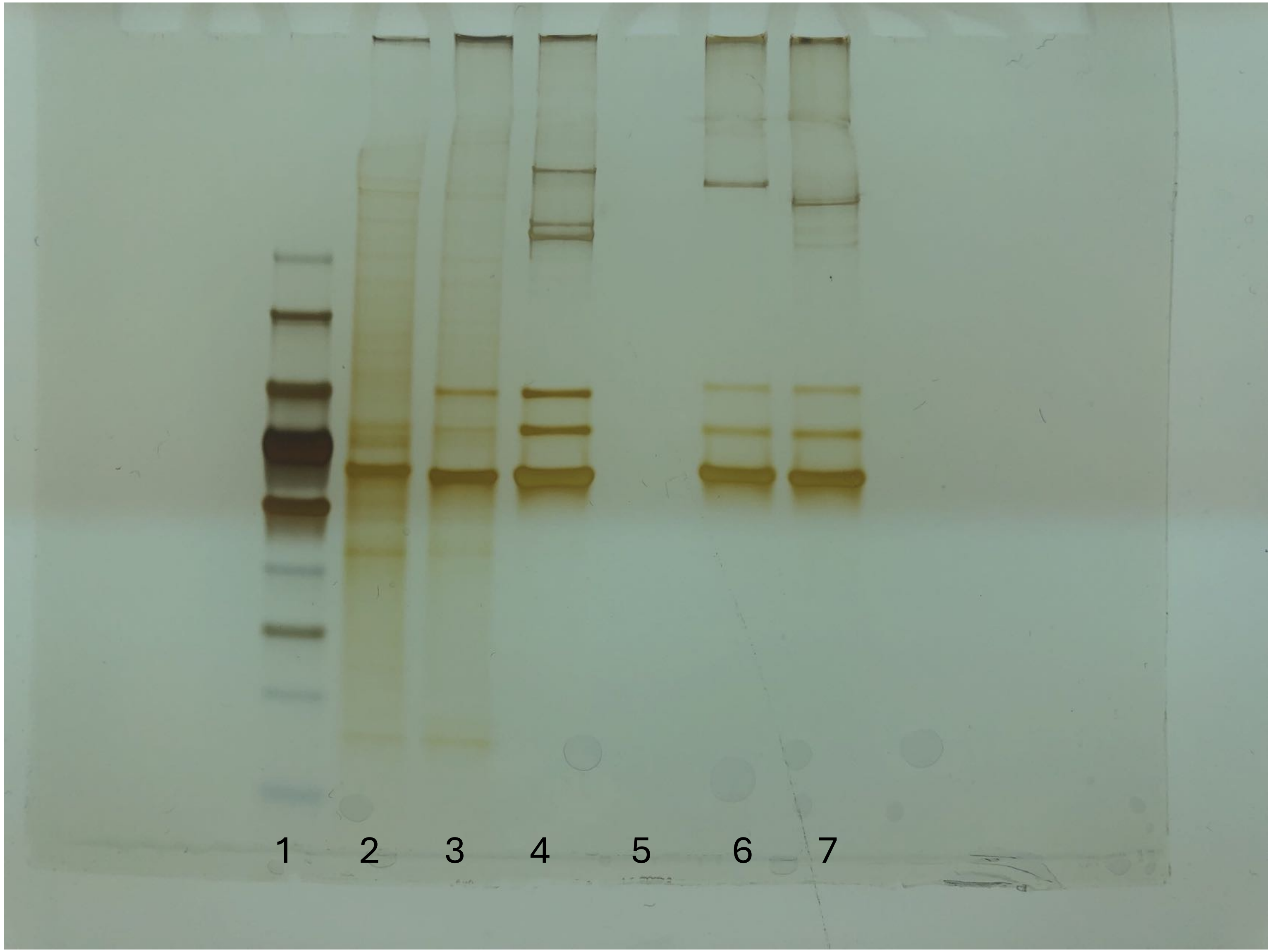

Lane 1: protein ladder: Bio-Rad: 161-0373
Lane 2-5: AAVs
Lane 5: empty

Lane 6: AAV6-GFPNaviPA1
Lane 7: AAV6-GFPNP

Full unedited gels for Fig. S2B and C. Western blots of Nav1.8 and NavB2 on HKE18 cell lysates

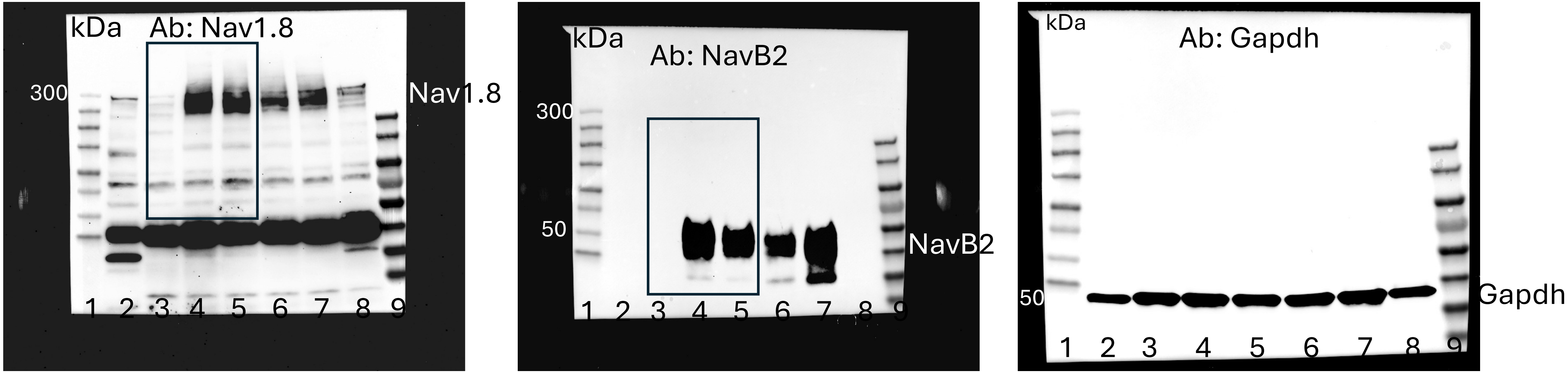

All blots:
Lane 1: protein ladder (thermos 26625)
Lane 9: Protein ladder (thermos 26616)
Lane 4: Naïve HEK cell lysates
Lane 5: Naïve HEK cell lysates
Lane 6: HEK1.8 cell lysates (clone 1)
Lane 7: HEK1.8 cell lysates (clone 2)
Lane 8: HEK1.8 cell lysates (clone 3)
Lane 9: HEK1.8 cell lysates (clone 4)

Square areas:
Presentation in Fig. S2B and C

Full unedited gels for **Fig. S2D**; Western blots of Nav1.8 and NavB2 (HEK1.8 cells)

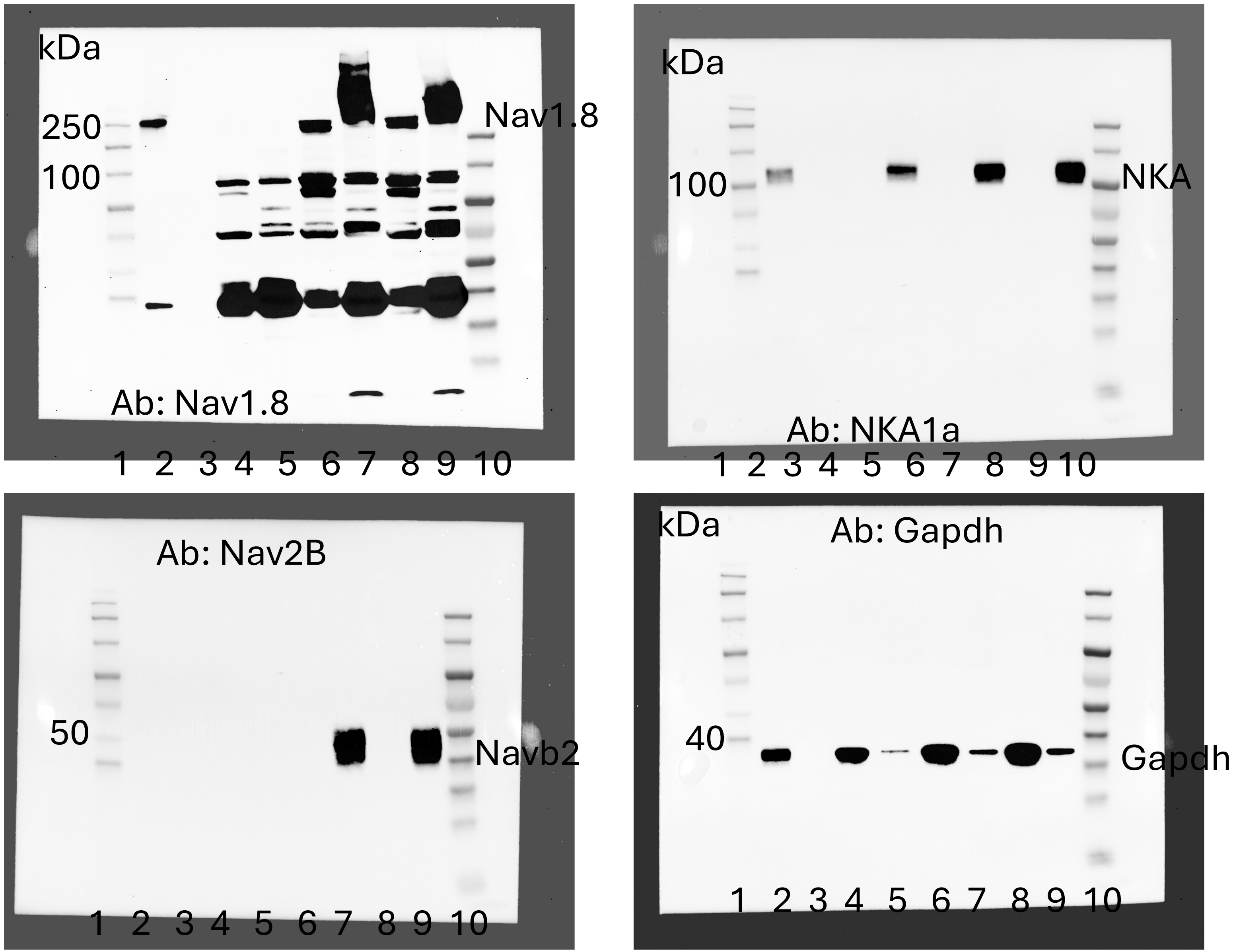

All blots:

Lane 1: protein ladder (thermos 26625)
Lane 10: Protein ladder (thermos 26616)
Lanes 2: Rat DRG homogenate
Lane 3: empty
Lane 4: Naïve HEK cell cytosol
Lane 5: Naïve HEK cell membrane
Lane 6: HEK1.8 cells (clone 1) cytosol
Lane 7: HEK1.8 cells (clone 1) membrane
Lane 8: HEK1.8 cells (clone 2) cytosol
Lane 9: HEK1.8 cells (clone 2) membrane
